# Supplementary material for: Bromodomain Protein BRD4 Is a Transcriptional Repressor of Autophagy and Lysosomal Function
Source: Mol Cell. 2017 May 18;66(4):517–532.e9. doi: 10.1016/j.molcel.2017.04.027 (PMC5446411; doi:10.1016/j.molcel.2017.04.027)
Supplement: Document S1. Figures S1–S7 [file mmc1.pdf]

Molecular Cell, Volume *66*

Supplemental Information

Bromodomain Protein BRD4 Is a Transcriptional Repressor of Autophagy and Lysosomal Function

Jun-ichi Sakamaki, Simon Wilkinson, Marcel Hahn, Nilgun Tasdemir, Jim O'Prey, William Clark, Ann Hedley, Colin Nixon, Jaclyn S. Long, Maria New, Tim Van Acker, Sharon A. Tooze, Scott W. Lowe, Ivan Dikic, and Kevin M. Ryan

Figure S1

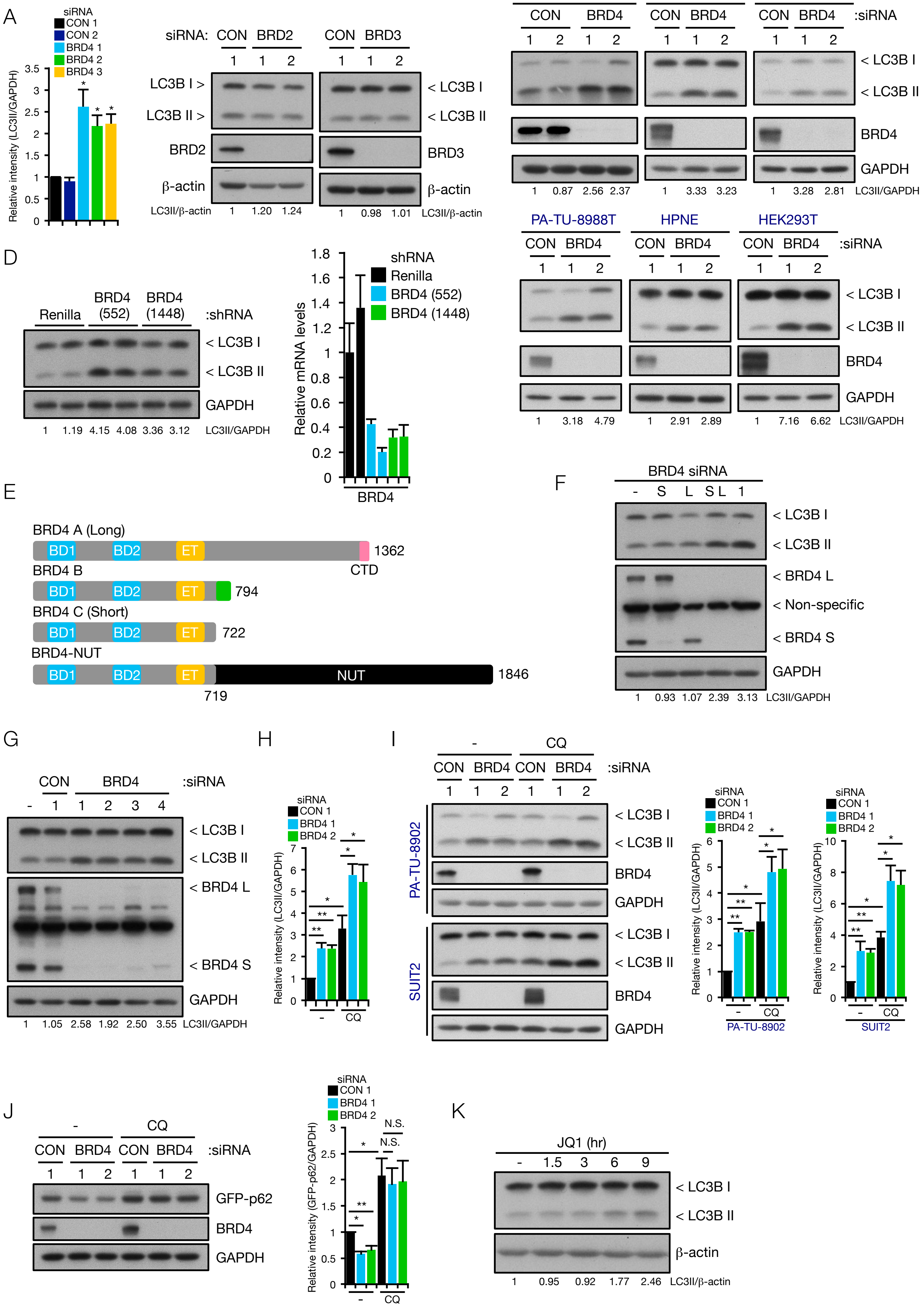

A
B
C
D
E
F
G
H
I
J
K
siRNA
CON 1
CON 2
BRD4 1
BRD4 2
BRD4 3
Relative intensity (LC3II/GAPDH)
siRNA: CON BRD2 CON BRD3
LC3B I
LC3B II
BRD2
BRD3
β-actin
LC3II/β-actin 1 1.20 1.24
1 0.98 1.01 LC3II/β-actin
PA-TU-8902
SUIT2
PK-1
CON BRD4 :siRNA
BRD4
GAPDH
1 0.87 2.56 2.37
1 3.33 3.23
1 3.28 2.81 LC3II/GAPDH
PA-TU-8988T
HPNE
HEK293T
1 3.18 4.79
1 2.91 2.89
1 7.16 6.62 LC3II/GAPDH
Renilla
BRD4 (552)
BRD4 (1448)
:shRNA
1 1.19 4.15 4.08 3.36 3.12 LC3II/GAPDH
shRNA
Relative mRNA levels
BRD4
BRD4 A (Long)
BD1
BD2
ET
1362
CTD
BRD4 B
794
BRD4 C (Short)
722
BRD4-NUT
NUT
1846
719
BRD4 siRNA
- S L S L 1
BRD4 L
Non-specific
BRD4 S
1 0.93 1.07 2.39 3.13 LC3II/GAPDH
CON BRD4 :siRNA
- 1 1 2 3 4
1 1.05 2.58 1.92 2.50 3.55 LC3II/GAPDH
CON 1
BRD4 1
BRD4 2
CQ
GFP-p62
Relative intensity (GFP-p62/GAPDH)
N.S.
JQ1 (hr)
- 1.5 3 6 9
1 0.95 0.92 1.77 2.46 LC3II/β-actin

Figure S1 Continued

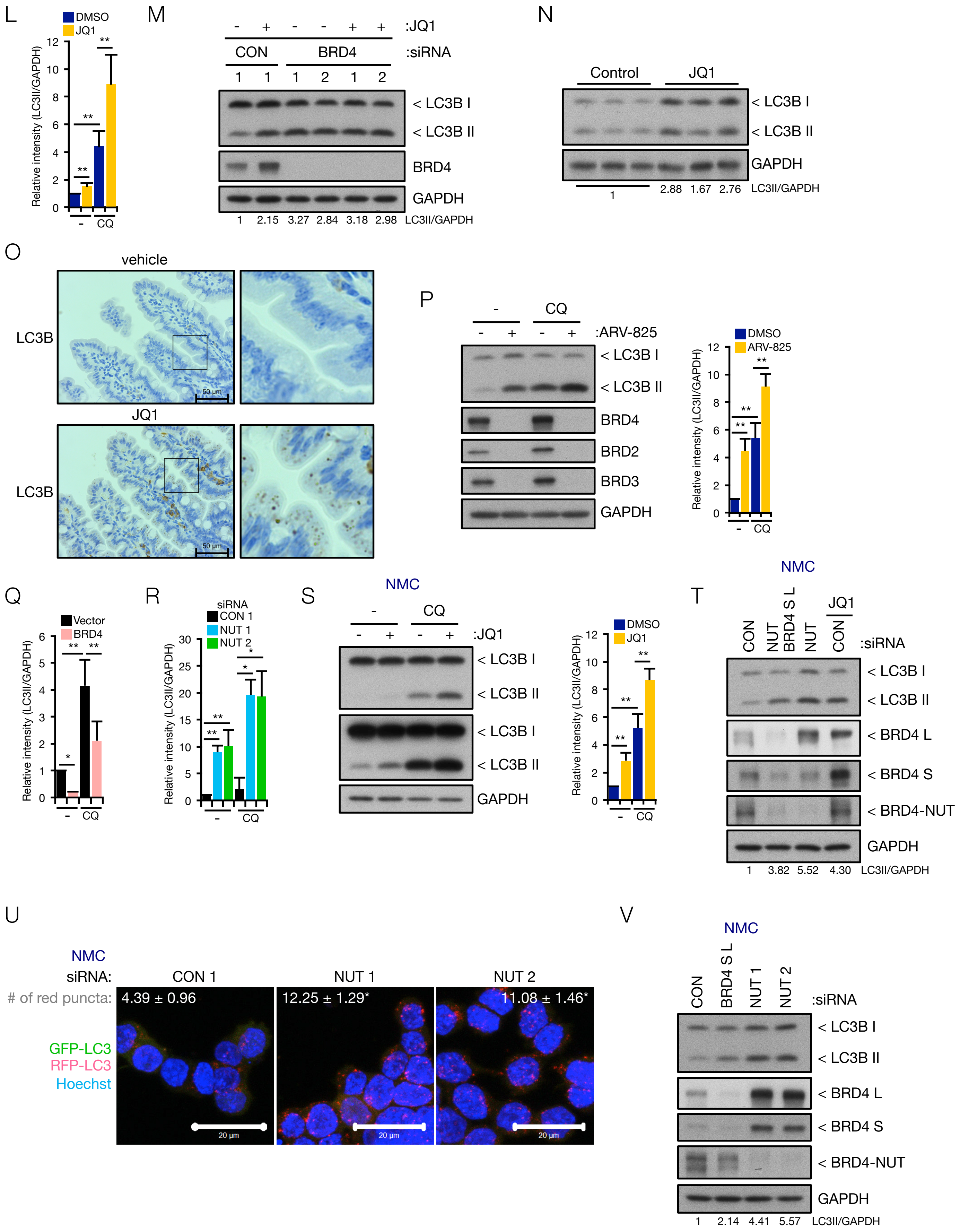

Figure S1, related to Figure 1. BRD4 silencing enhances autophagic flux

(A) Quantification of LC3II signal intensity normalized to GAPDH levels in Fig. 1B.

(B) KP-4 cells were transfected with BRD2 (left) or BRD3 (right) siRNA for 72 hrs.

(C) BRD4 siRNA was transfected into PA-TU-8902, SUIT2, PK-1, PA-TU-8988T, HPNE, and HEK293T cells for 72 hrs.

(D) Proteins (left) and RNA (right) were extracted from FFPE small intestinal tissues of transgenic mice harboring inducible renilla luciferase or BRD4 shRNA. LC3II levels were monitored by western blot analysis (left) and knockdown of BRD4 was confirmed by RT-qPCR (right).

(E) Domain structure of BRD4 isoforms and BRD4-NUT. BD: Bromodomain, ET: Extraterminal domain, CTD: carboxy-terminal domain.

(F, G) KP-4 cells were transfected with siRNA targeting BRD4 short (S) and/or long (L) isoforms for 72 hrs (F). All BRD4 siRNAs used in the experiments target both long and short isoforms (G).

(H) Quantification of LC3II signal intensity normalized to GAPDH levels in Fig. 1E.

(I) PA-TU-8902 (upper left) and SUIT2 (lower left) transfected with BRD4 siRNA were treated with 10 μM CQ for 4 hrs. Quantification of LC3II signal intensity is shown in the middle (PA-TU-8902) and right (SUIT2) panels.

(J) KP-4 cells were transfected with control or BRD4 siRNA. At 24 hrs after siRNA transfection, cells were transfected with GFP-p62 expression vector for 48 hrs. At 16 hrs after GFP-p62 transfection, cells were treated with or without 10 μM CQ for 32 hrs. Quantification of GFP-p62 levels is shown in the right panel.

(K) KP-4 cells were treated with 500 nM of JQ1 and harvested at indicated time points.

(L) Quantification of LC3II signal intensity normalized to GAPDH levels in Fig. 1H.

(M) KP-4 cells transfected with control or BRD4 siRNA were treated with 500 nM JQ1 for 9 hrs.

(N) Proteins were extracted from FFPE small intestinal tissues of control vehicle and JQ1-treated mice.

(O) Immunohistochemical staining for LC3 in small intestinal sections from mice treated with control vehicle or JQ1. Scale bars: 50 μm.

(P) KP-4 cells were treated with 100 nM ARV-825 for 16 hrs in the presence or absence of CQ (10 μM, 4 hrs). Quantification of LC3II levels is shown in the right panel.

(Q, R) Quantification of LC3II signal intensity normalized to GAPDH levels in Fig. 1I (Q) and Fig. 1J (R).

(S) TY-82 cells were treated with 500 nM JQ1 for 9 hrs in the presence or absence of CQ (10 μM, 8 hrs). Quantification of LC3II levels is shown in the right panel.

(T) TY-82 cells were transfected with siRNA against control, NUT alone or NUT together with BRD4 S and L isoforms for 5 days. 500 nM JQ1 was treated for 9 hrs.

(U) TY-82 cells stably expressing RFP-GFP-LC3 were transfected with NUT siRNA for 5 days. The number of $GFP\text{-}LC3^{-}$/$RFP\text{-}LC3^{+}$ puncta normalized to cell number is shown. CON n=133 cells, BRD4 1 n=115 cells, BRD4 2 n=105 cells. Scale bars: 20 μm.

(V) TY-82 cells were transfected with siRNAs targeting BRD4 short and long isoforms or NUT for 5 days.

All data are shown as mean ± SD. A, H-J, L, and P-S: n=3 independent experiments. $^{*}P < 0.01$, $^{**}P < 0.05$, N.S.: no significance.

Figure S2

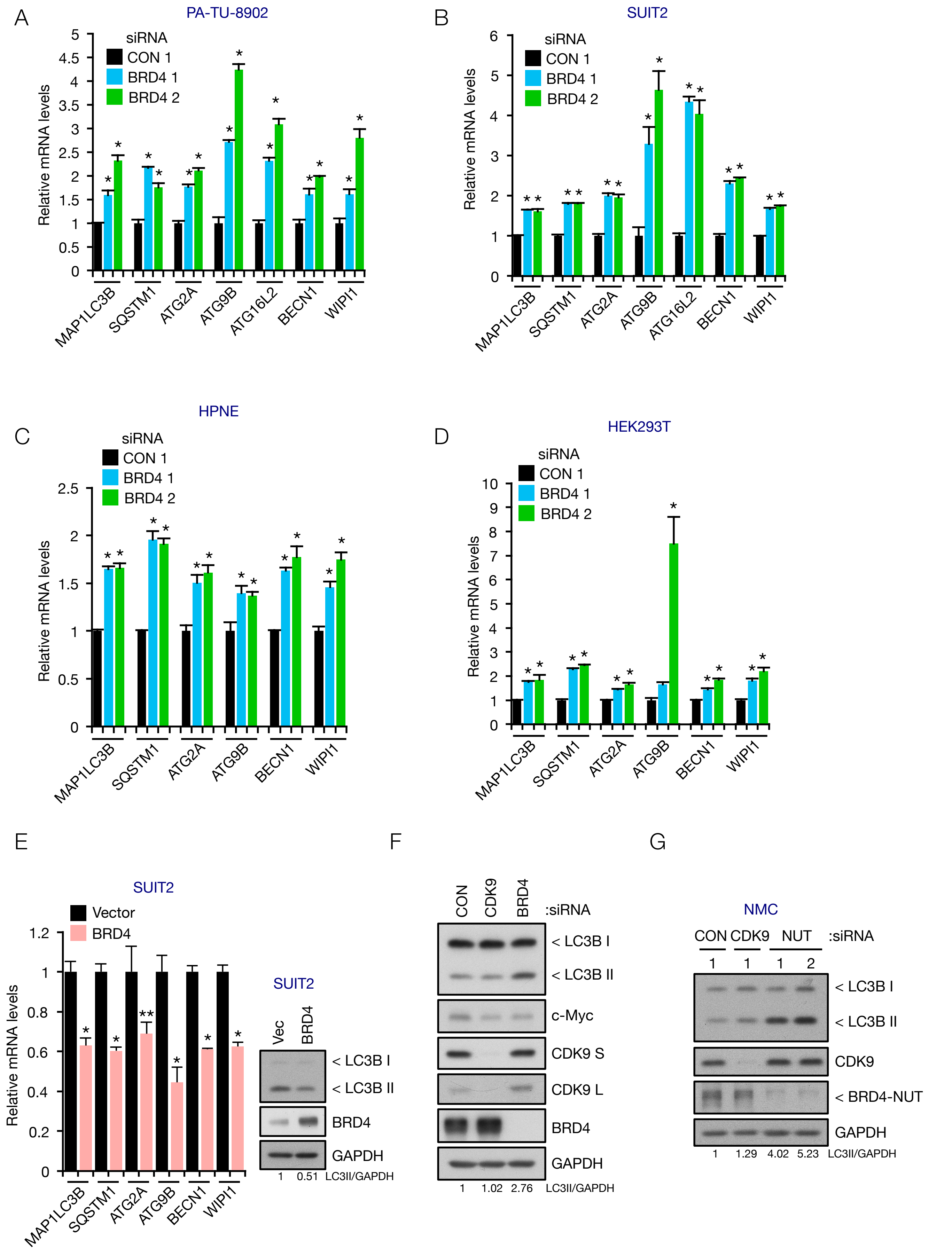
A
PA-TU-8902
siRNA
CON 1
BRD4 1
BRD4 2
Relative mRNA levels
MAP1LC3B
SQSTM1
ATG2A
ATG9B
ATG16L2
BECN1
WIPI1
B
SUIT2
C
HPNE
D
HEK293T
E
SUIT2
Vector
BRD4
F
CON
CDK9
BRD4
:siRNA
< LC3B I
< LC3B II
c-Myc
CDK9 S
CDK9 L
BRD4
GAPDH
1 1.02 2.76 LC3II/GAPDH
SUIT2
Vec
BRD4
< LC3B I
< LC3B II
BRD4
GAPDH
1 0.51 LC3II/GAPDH
G
NMC
CON CDK9 NUT :siRNA
1 1 1 2
< LC3B I
< LC3B II
CDK9
< BRD4-NUT
GAPDH
1 1.29 4.02 5.23 LC3II/GAPDH

Figure S2, related to Figure 2. BRD4 is a negative regulator of autophagy gene expression
(A-D) RT-qPCR analyses of PA-TU-8902 (A), SUIT2 (B), HPNE (C), and HEK293T (D) cells transfected with BRD4 siRNA for 72 hrs.
(E) RT-qPCR analysis of SUIT2 cells stably overexpressing BRD4. Right panel shows autophagy inhibition by BRD4 overexpression.
(F) KP-4 cells were transfected with control, CDK9, or BRD4 siRNA for 72 hrs.
(G) TY-82 cells were transfected with siRNA targeting CDK9 or NUT for 5 days.
All data are shown as mean ± SD. A-E: Data are representative of two independent experiments performed in triplicate. $^*P < 0.01$, $^{**}P < 0.05$.

Figure S3

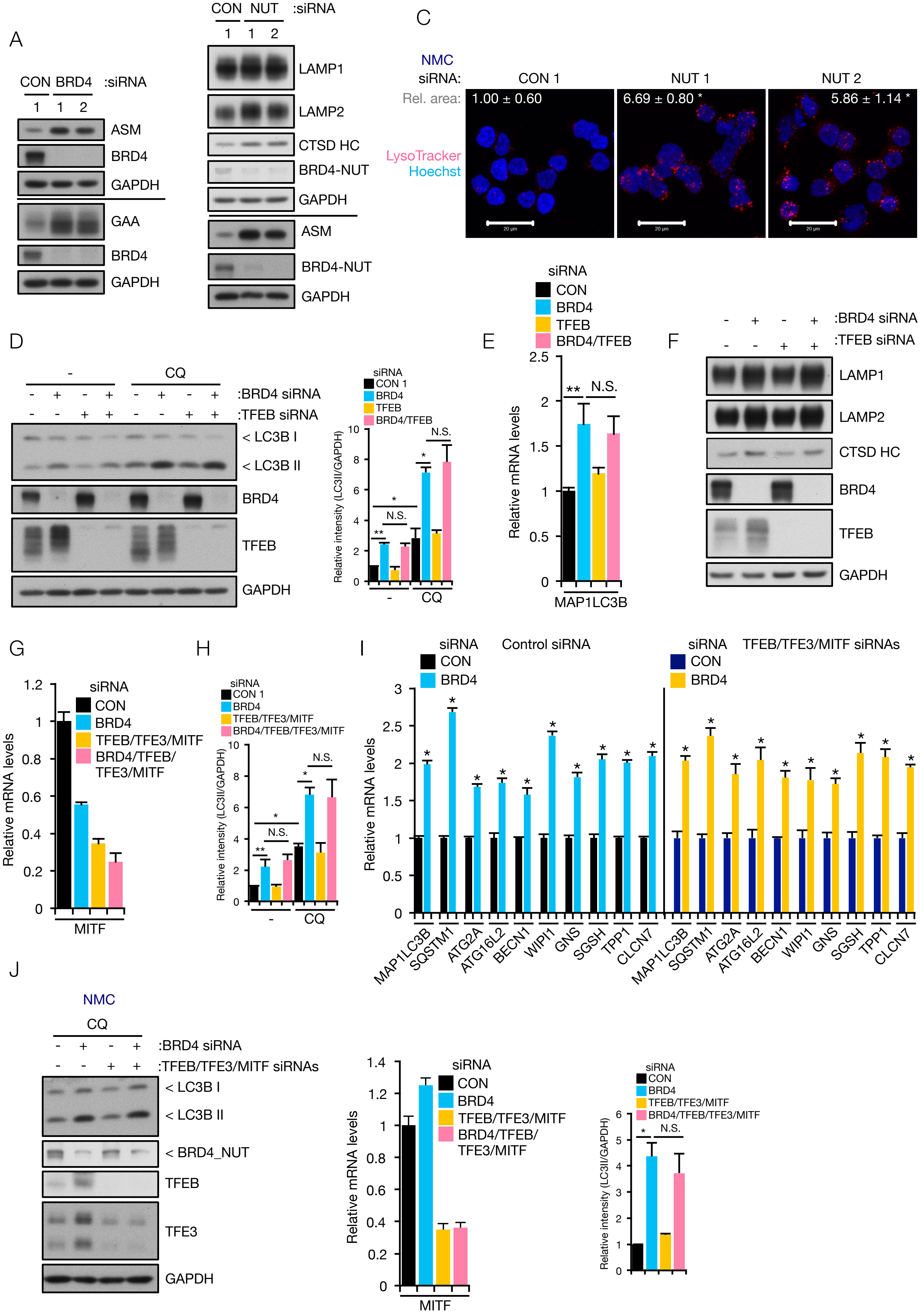
A
CON BRD4 :siRNA
1 1 2
ASM
BRD4
GAPDH
GAA
BRD4
GAPDH
B
NMC
CON NUT :siRNA
1 1 2
LAMP1
LAMP2
CTSD HC
BRD4-NUT
GAPDH
ASM
BRD4-NUT
GAPDH
C
NMC
siRNA:
CON 1
NUT 1
NUT 2
Rel. area:
1.00 ± 0.60
6.69 ± 0.80 *
5.86 ± 1.14 *
LysoTracker
Hoechst
20 µm
D
-
CQ
:BRD4 siRNA
:TFEB siRNA
< LC3B I
< LC3B II
BRD4
TFEB
GAPDH
siRNA
CON 1
BRD4
TFEB
BRD4/TFEB
Relative intensity (LC3II/GAPDH)
N.S.
**
E
siRNA
CON
BRD4
TFEB
BRD4/TFEB
Relative mRNA levels
MAP1LC3B
F
:BRD4 siRNA
:TFEB siRNA
LAMP1
LAMP2
CTSD HC
BRD4
TFEB
GAPDH
G
siRNA
CON
BRD4
TFEB/TFE3/MITF
BRD4/TFEB/
TFE3/MITF
Relative mRNA levels
MITF
H
siRNA
CON 1
BRD4
TFEB/TFE3/MITF
BRD4/TFEB/TFE3/MITF
Relative intensity (LC3II/GAPDH)
CQ
I
siRNA
Control siRNA
CON
BRD4
TFEB/TFE3/MITF siRNAs
Relative mRNA levels
MAP1LC3B
SQSTM1
ATG2A
ATG16L2
BECN1
WIPI1
GNS
SGSH
TPP1
CLCN7
J
NMC
CQ
:BRD4 siRNA
:TFEB/TFE3/MITF siRNAs
< LC3B I
< LC3B II
< BRD4_NUT
TFEB
TFE3
GAPDH
siRNA
CON
BRD4
TFEB/TFE3/MITF
BRD4/TFEB/
TFE3/MITF
Relative mRNA levels
MITF
Relative intensity (LC3II/GAPDH)

Figure S3, related to Figure 3. BRD4 knockdown enhances lysosomal function

(A) KP-4 cells transfected with BRD4 siRNA were subjected to western blot analysis with antibodies against lysosomal proteins.

(B) TY-82 cells transfected with NUT siRNA were subjected to western blot analysis with antibodies against lysosomal proteins.

(C) TY-82 cells transfected with NUT siRNA were stained with LysoTracker Red (100 nM, 4 hrs). Area of $LysoTracker^{+}$ compartments normalized to cell number is shown (CON n=189 cells, NUT 1 n=101 cells, NUT 2 n=101 cells). Scale bars: 20 μm.

(D) BRD4 siRNA was transfected into KP-4 cells together with siRNA against TFEB followed by treatment with 10 μM CQ for 4 hrs. Quantification of LC3II levels is shown in the right.

(E) BRD4 siRNA was transfected into KP-4 cells together with siRNA against TFEB. MAP1LC3B mRNA levels were monitored at 72 hrs after transfection.

(F) BRD4 siRNA was transfected into KP-4 cells together with siRNA against TFEB for 72 hrs followed by western blot analysis with indicated antibodies against lysosomal proteins.

(G) Confirmation of MITF knockdown in Fig. 3H. KP-4 cells transfected with BRD4 and/or MiT/TFE (TFEB, TFE3, MITF) siRNAs were subjected to RT-qPCR analysis.

(H) Quantification of LC3II levels normalized to GAPDH levels in Fig. 3H.

(I) RT-qPCR analysis of KP-4 cells transfected with BRD4 and/or MiT/TFE (TFEB, TFE3, MITF) siRNAs.

(J) TY-82 cells were transfected with NUT and/or MiT/TFE (TFEB, TFE3, MITF) siRNAs and treated with 10 μM CQ for 8 hrs. Quantification of LC3II levels is shown in the middle panel. Knockdown of MITF was confirmed by RT-qPCR (right panel).

All data are shown as mean ± SD. D, E, H, and J: n=3 independent experiments. I: Data are representative of two independent experiments performed in triplicate. $^{*}P < 0.01$, $^{**}P < 0.05$, N.S.: no significance.

Figure S4

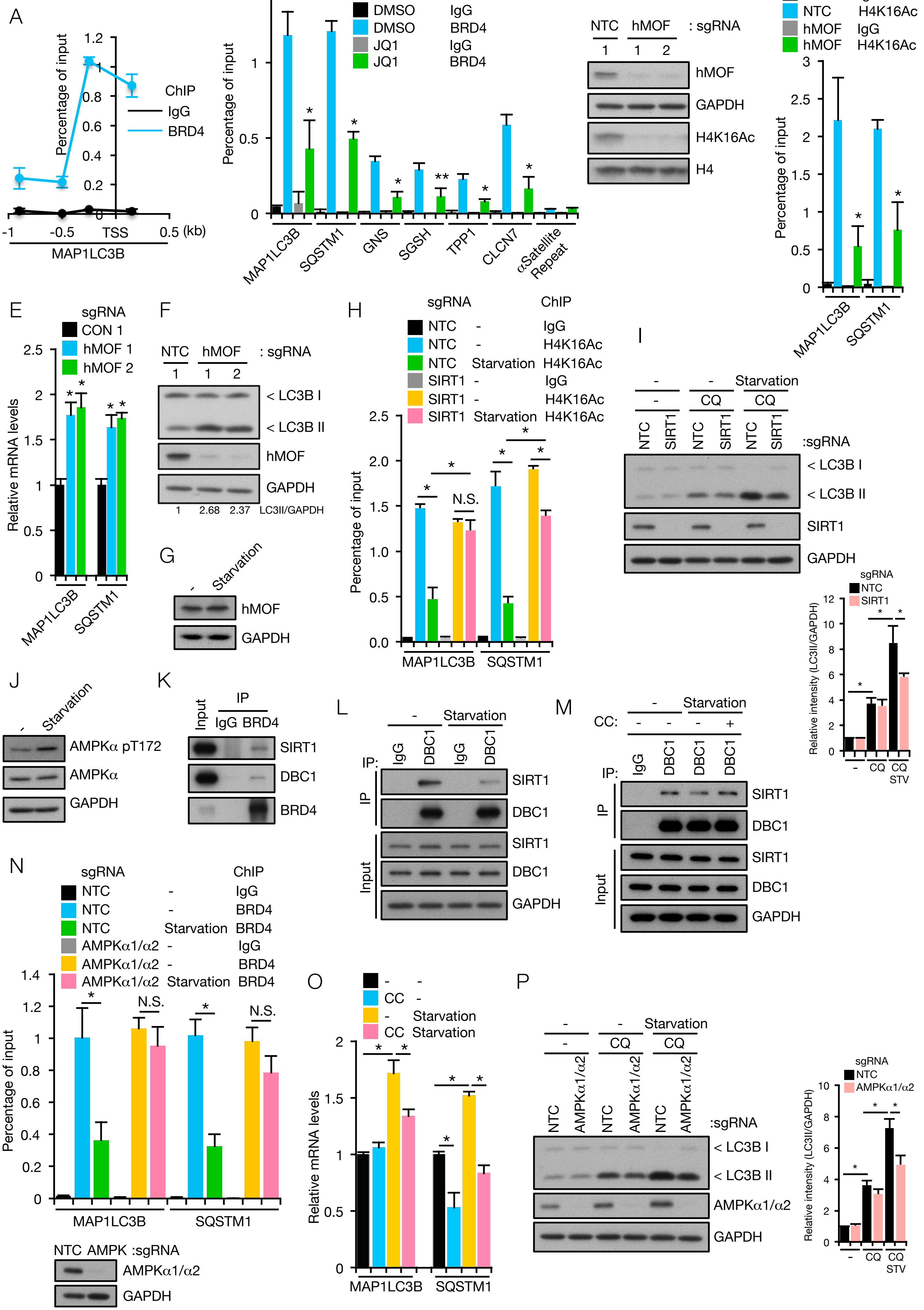
A
Percentage of input
ChIP
IgG
BRD4
-1
-0.5
TSS
0.5 (kb)
MAP1LC3B
B
ChIP
DMSO IgG
DMSO BRD4
JQ1 IgG
JQ1 BRD4
Percentage of input
MAP1LC3B
SQSTM1
GNS
SGSH
TPP1
CLCN7
αSatellite Repeat
C
NTC
hMOF
: sgRNA
hMOF
GAPDH
H4K16Ac
H4
D
sgRNA ChIP
NTC IgG
NTC H4K16Ac
hMOF IgG
hMOF H4K16Ac
Percentage of input
MAP1LC3B
SQSTM1
E
sgRNA
CON 1
hMOF 1
hMOF 2
Relative mRNA levels
MAP1LC3B
SQSTM1
F
NTC
hMOF
: sgRNA
< LC3B I
< LC3B II
hMOF
GAPDH
1 2.68 2.37 LC3II/GAPDH
G
Starvation
hMOF
GAPDH
H
sgRNA ChIP
NTC - IgG
NTC - H4K16Ac
NTC Starvation H4K16Ac
SIRT1 - IgG
SIRT1 - H4K16Ac
SIRT1 Starvation H4K16Ac
Percentage of input
N.S.
MAP1LC3B
SQSTM1
I
Starvation
CQ
CQ
NTC
SIRT1
:sgRNA
< LC3B I
< LC3B II
SIRT1
GAPDH
sgRNA
NTC
SIRT1
Relative intensity (LC3II/GAPDH)
CQ
CQ STV
J
Starvation
AMPKα pT172
AMPKα
GAPDH
K
Input
IP
IgG BRD4
SIRT1
DBC1
BRD4
L
Starvation
IP:
IgG
DBC1
IP
Input
SIRT1
DBC1
SIRT1
DBC1
GAPDH
M
CC:
Starvation
IP:
IgG
DBC1
IP
Input
SIRT1
DBC1
SIRT1
DBC1
GAPDH
N
sgRNA ChIP
NTC - IgG
NTC - BRD4
NTC Starvation BRD4
AMPKα1/α2 - IgG
AMPKα1/α2 - BRD4
AMPKα1/α2 Starvation BRD4
Percentage of input
N.S.
MAP1LC3B
SQSTM1
NTC AMPK :sgRNA
AMPKα1/α2
GAPDH
O
- -
CC -
- Starvation
CC Starvation
Relative mRNA levels
MAP1LC3B
SQSTM1
P
Starvation
CQ
CQ
NTC
AMPKα1/α2
:sgRNA
< LC3B I
< LC3B II
AMPKα1/α2
GAPDH
sgRNA
NTC
AMPKα1/α2
Relative intensity (LC3II/GAPDH)
CQ
CQ STV

Figure S4, related to Figure 4. Starvation leads to BRD4 dissociation from autophagy gene promoters
(A) KP-4 cells were subjected to ChIP assay using control IgG and BRD4 antibody followed by qPCR analysis using primers for different regions of MAP1LC3B gene.
(B) KP-4 cells treated with 500 nM JQ1 for 9 hrs followed by ChIP assay with BRD4 antibody.
(C-F) KP-4 cells infected with Cas9/hMOF sgRNA were subjected to western blot analyses with hMOF and H4K16Ac (C) and LC3 (F) antibodies, ChIP assay with H4K16Ac antibody (D), and RT-qPCR analysis (E).
(G) KP-4 cells were starved for 4 hrs followed by western blot analysis with hMOF antibody.
(H) KP-4 cells infected with Cas9/SIRT1 sgRNA were starved for 4 hrs followed by ChIP assay with H4K16Ac antibody.
(I) KP-4 cells infected with Cas9/SIRT1 sgRNA were treated with 10 μM CQ for 4 hrs. At 2 hrs after CQ treatment, cells were subjected to starvation for 2 hrs in the presence of CQ. Quantification of LC3II levels is shown in the lower right panel.
(J) KP-4 cells were starved for 4 hrs and subjected to western blot analysis.
(K) Cell extracts from KP-4 cells were subjected to immunoprecipitation with BRD4 antibody.
(L) Lysates from KP-4 cells starved for 4 hrs were subjected to immunoprecipitation with DBC1 antibody.
(M) KP-4 cells pre-treated with 10 μM Compound C (CC, 3 hrs) were starved for 4 hrs in the presence of 10 μM CC. Cell lysates were then subjected to immunoprecipitation with DBC1 antibody.
(N) KP-4 cells infected with Cas9/AMPKα1/α2 sgRNAs were starved for 4 hrs followed by ChIP assay with BRD4 antibody. Western blot shows efficient AMPKα1/α2 depletion in Cas9/AMPKα1/α2 sgRNA-infected cells.
(O) KP-4 cells pre-treated with 10 μM Compound C (CC, 3 hrs) were starved for 4 hrs in the presence of 10 μM CC.
(P) KP-4 cells infected with Cas9/AMPKα1/α2 sgRNAs were treated with 10 μM CQ for 4 hrs. At 2 hrs after CQ treatment, cells were subjected to starvation for 2 hrs in the presence of CQ. Quantification of LC3II levels is shown in the right panel.
All data are shown as mean ± SD. B, D, E, I, N-P: n=3 independent experiments. A and H: Data are representative of two independent experiments performed in triplicate. $^{*}P < 0.01$, $^{**}P < 0.05$, N.S.: no significance.

Figure S5

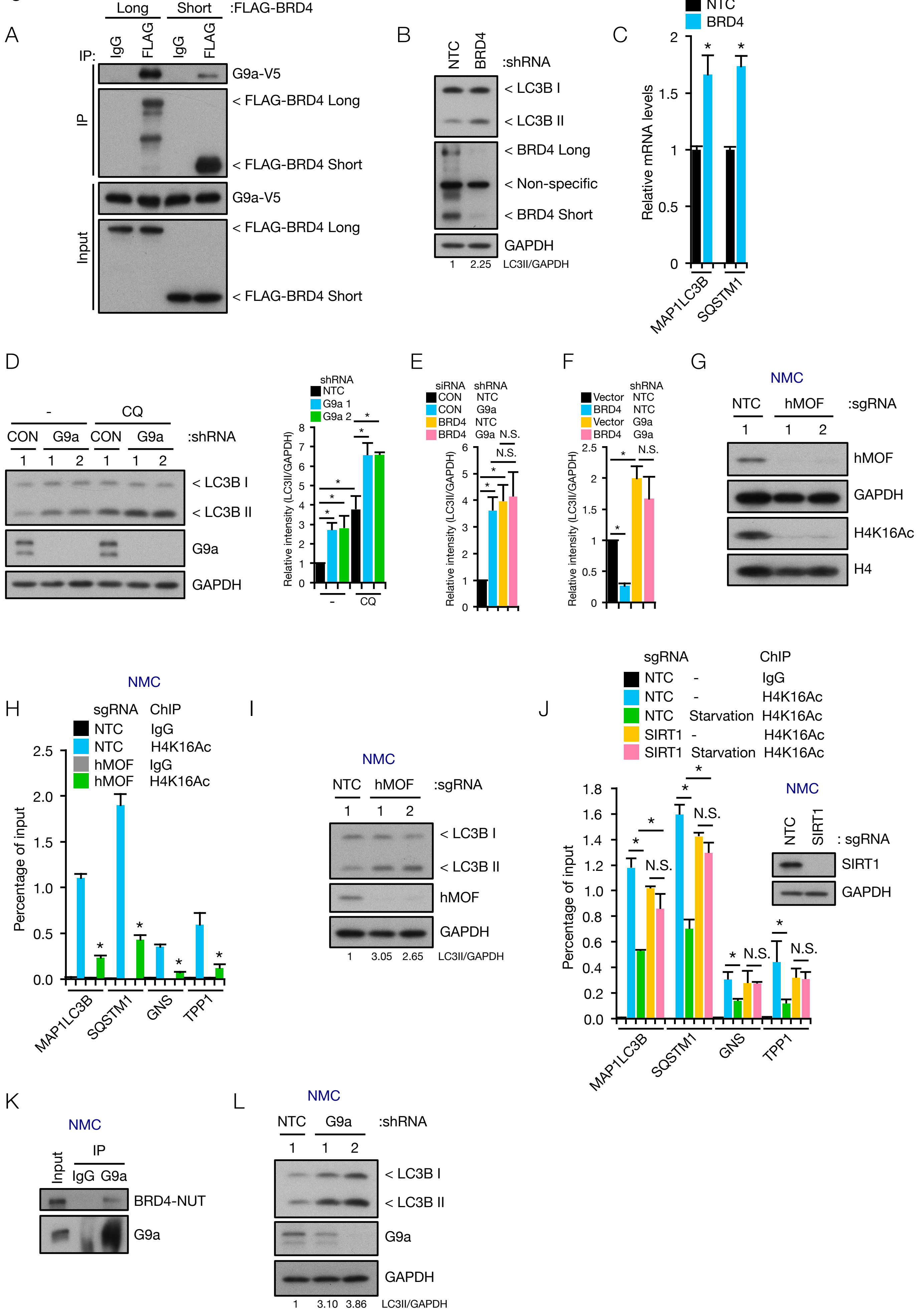

A
Long
Short
:FLAG-BRD4
IP:
IgG
FLAG
IgG
FLAG
IP
Input
G9a-V5
< FLAG-BRD4 Long
< FLAG-BRD4 Short
G9a-V5
< FLAG-BRD4 Long
< FLAG-BRD4 Short
B
NTC
BRD4
:shRNA
< LC3B I
< LC3B II
< BRD4 Long
< Non-specific
< BRD4 Short
GAPDH
1
2.25
LC3II/GAPDH
C
shRNA
NTC
BRD4
Relative mRNA levels
2
1.5
1
0.5
0
*
*
MAP1LC3B
SQSTM1
D
-
CQ
CON
G9a
CON
G9a
:shRNA
1
1
2
1
1
2
< LC3B I
< LC3B II
G9a
GAPDH
shRNA
NTC
G9a 1
G9a 2
Relative intensity (LC3II/GAPDH)
8
7
6
5
4
3
2
1
0
*
*
*
*
*
-
CQ
E
siRNA
shRNA
CON
NTC
CON
G9a
BRD4
NTC
BRD4
G9a
N.S.
Relative intensity (LC3II/GAPDH)
6
5
4
3
2
1
0
N.S.
*
*
F
shRNA
Vector
NTC
BRD4
NTC
Vector
G9a
BRD4
G9a
Relative intensity (LC3II/GAPDH)
2.5
2
1.5
1
0.5
0
*
N.S.
*
G
NMC
NTC
hMOF
:sgRNA
1
1
2
hMOF
GAPDH
H4K16Ac
H4
H
NMC
sgRNA
ChIP
NTC
IgG
NTC
H4K16Ac
hMOF
IgG
hMOF
H4K16Ac
Percentage of input
2.5
2.0
1.5
1.0
0.5
0.0
*
*
*
*
MAP1LC3B
SQSTM1
GNS
TPP1
I
NMC
NTC
hMOF
:sgRNA
1
1
2
< LC3B I
< LC3B II
hMOF
GAPDH
1
3.05
2.65
LC3II/GAPDH
J
sgRNA
ChIP
NTC
-
IgG
NTC
-
H4K16Ac
NTC
Starvation
H4K16Ac
SIRT1
-
H4K16Ac
SIRT1
Starvation
H4K16Ac
Percentage of input
1.8
1.6
1.4
1.2
1.0
0.8
0.6
0.4
0.2
0.0
*
*
N.S.
*
*
N.S.
*
N.S.
*
N.S.
MAP1LC3B
SQSTM1
GNS
TPP1
NMC
NTC
SIRT1
: sgRNA
SIRT1
GAPDH
K
NMC
Input
IP
IgG
G9a
BRD4-NUT
G9a
L
NMC
NTC
G9a
:shRNA
1
1
2
< LC3B I
< LC3B II
G9a
GAPDH
1
3.10
3.86
LC3II/GAPDH

Figure S5, related to Figure 5. BRD4 represses autophagy gene expression through G9a

(A) HEK293T cells transfected with G9a-V5 together with FLAG-BRD4 long or short isoform were subjected to immunoprecipitation with control IgG or FLAG antibody.

(B, C) Validation of inducible BRD4 knockdown cells. KP-4 cells harboring inducible control or BRD4 shRNA were treated with 500 ng/ml doxycycline (DOX) for 4 days and subjected to western blot (B) and RT-qPCR (C) analyses.

(D) KP-4 cells infected with lentivirus expressing G9a shRNA were treated with 10 μM CQ for 4 hrs. Quantification of LC3II levels is shown in the right panel.

(E, F) Quantification of LC3II signal intensity normalized to GAPDH levels in Fig. 5H (E) and Fig. 5I (F).

(G-I) TY-82 cells were infected with Cas9/hMOF sgRNA followed by western blot analysis with hMOF and H4K16Ac (G) and LC3B (I) antibodies and ChIP assay with H4K16Ac antibody (H).

(J) TY-82 cells infected with Cas9/SIRT1 sgRNA were starved for 4 hrs followed by ChIP assay with H4K16Ac antibody. Western blot shows efficient SIRT1 depletion in Cas9/SIRT1 sgRNA-infected cells.

(K) Cell extracts from TY-82 cells were immunoprecipitated with G9a antibody.

(L) TY-82 cells were infected with shRNA targeting G9a followed by western blot analysis.

All data are shown as mean ± SD. C-F: n=3 independent experiments. H and J: Data are representative of two independent experiments performed in triplicate. *$P < 0.01$, **$P < 0.05$, N.S.: no significance.

Figure S6

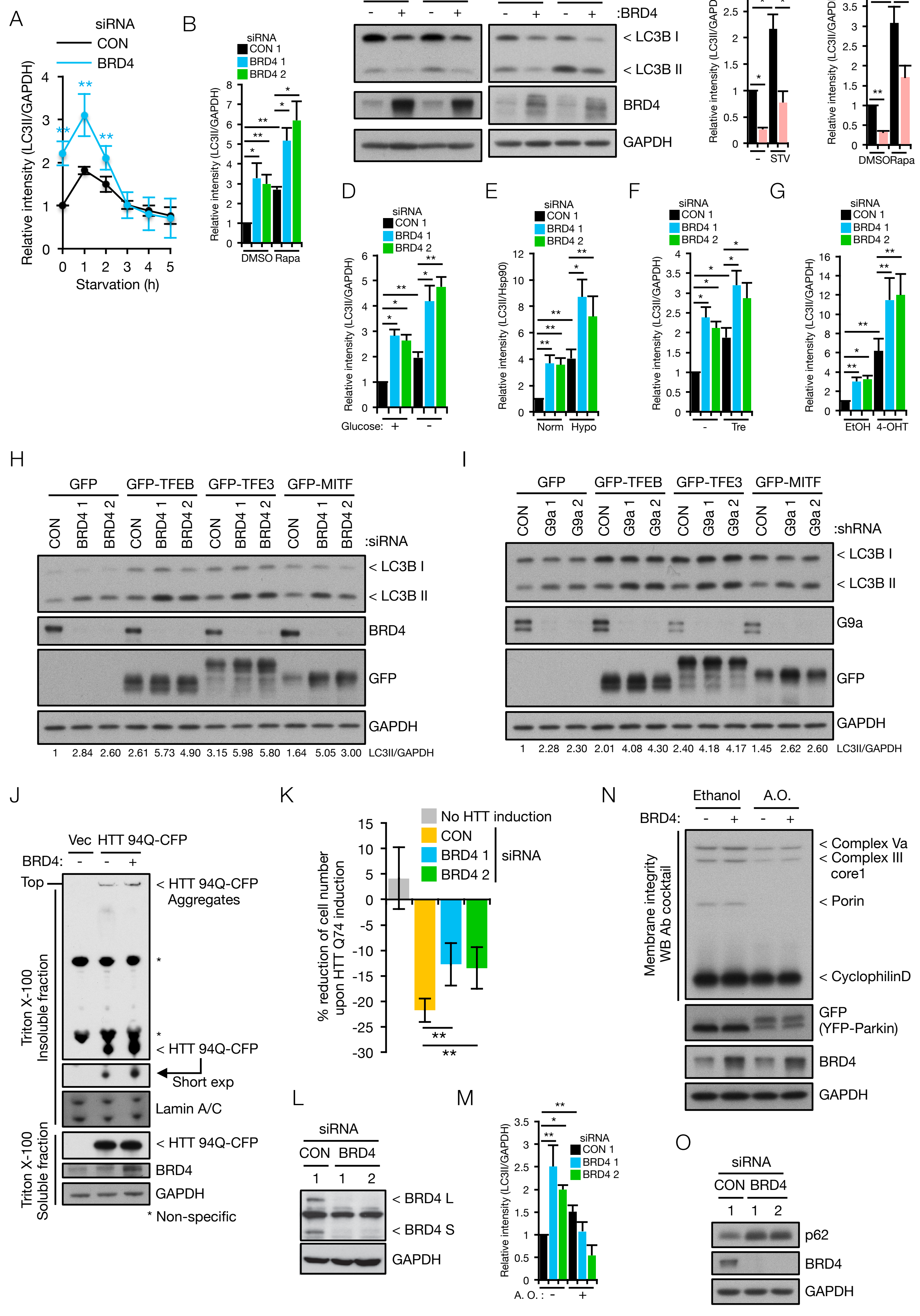

A
siRNA
CON
BRD4
Relative intensity (LC3II/GAPDH)
Starvation (h)
B
DMSO Rapa
C
Starvation
DMSO
Rapa
:BRD4
< LC3B I
< LC3B II
BRD4
GAPDH
Vector
BRD4
STV
D
Glucose:
E
Relative intensity (LC3II/Hsp90)
Norm Hypo
F
Tre
G
EtOH 4-OHT
H
GFP
GFP-TFEB
GFP-TFE3
GFP-MITF
:siRNA
1 2.84 2.60 2.61 5.73 4.90 3.15 5.98 5.80 1.64 5.05 3.00 LC3II/GAPDH
I
:shRNA
G9a
1 2.28 2.30 2.01 4.08 4.30 2.40 4.18 4.17 1.45 2.62 2.60 LC3II/GAPDH
J
Vec HTT 94Q-CFP
Top
< HTT 94Q-CFP Aggregates
Short exp
Lamin A/C
Triton X-100 Insoluble fraction
Triton X-100 Soluble fraction
* Non-specific
K
No HTT induction
% reduction of cell number upon HTT Q74 induction
L
< BRD4 L
< BRD4 S
M
A. O. :
N
Ethanol
A.O.
< Complex Va
< Complex III core1
< Porin
< CyclophilinD
Membrane integrity WB Ab cocktail
GFP (YFP-Parkin)
O
p62

Figure S6, related to Figure 6. Effect of BRD4 silencing on stimulus-dependent and selective autophagy

(A, B) Quantification of LC3II signal intensity normalized to GAPDH levels in Fig. 6A (A) and Fig. 6B (B).

(C) KP-4 cells stably overexpressing BRD4 were starved for 90 min (left western blot) or treated with 500 nM rapamycin for 24 hrs (right western blot). Quantification of LC3II levels is shown.

(D-G) Quantification of LC3II signal intensity normalized to GAPDH or HSP90 levels in Fig. 6C (D), Fig. 6D (E), Fig. 6E (F), and Fig. 6F (G).

(H, I) HEK293T cells were transfected with BRD4 siRNA (H) or infected with G9a shRNA (I) followed by transfection with MiT/TFE expression vectors.

(J) KP-4 cells expressing BRD4, rtTA, and Tre-tight-HTT Q94-CFP were treated with 1 μg/ml DOX for 10 hrs. At 48 hrs after removal of DOX, cells were harvested and separated into TritonX-100 soluble and insoluble fractions.

(K) KP-4 pLVX-GFP-HTT Q74 and control parental cells were transfected with control or BRD4 siRNA for 72 hrs. At 12 hrs after transfection, cells were treated with 100 ng/ml DOX for 60 hrs. Cell number of mutant HTT expressing cells was normalized to that of parental cells and presented as percentage of reduction in cell number upon mutant HTT induction.

(L) Confirmation of efficient BRD4 knockdown in Fig. 6H.

(M) Quantification of LC3II signal intensity normalized to GAPDH levels in Fig. 6I.

(N) KP-4 cells expressing YFP-parkin were infected with control or BRD4 expression vector. Cells were treated with 1 μM Antimycin A and 1 μM Oligomycin for 8 hrs.

(O) KP-4 cells were transfected with BRD4 siRNA for 72hrs.

All data are shown as mean ± SD. A-E, G, and M: n=3 independent experiments. F: n=4 independent experiments. K: n=5 independent experiments. *$P < 0.01$, **$P < 0.05$.

Figure S7

A

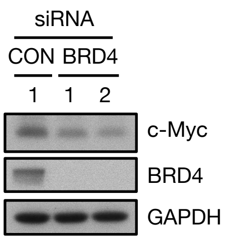

B

Gene Symbol	Fold change BRD4 1 vs Con 1	P value BRD4 1 vs Con 1	Fold change BRD4 2 vs Con 1	P value BRD4 2 vs Con 1
CDK1	-1.669	1.12371E-09	-2.155	1.81448E-19
CDK2	-1.559	1.04614E-09	-1.680	6.13946E-11
CCNA2	-1.292	0.000673956	-1.774	2.91219E-11
CCNB1	-1.756	1.27998E-08	-1.829	1.67277E-09
CCNB2	-2.080	9.82978E-13	-1.879	2.05674E-09
CCND2	-2.709	5.86107E-18	-1.477	0.001657272
CCNE2	-1.412	0.006004696	-1.799	9.32676E-07
CDC25B	-1.326	0.000756346	-1.634	1.39001E-08
CDC25C	-1.829	5.90101E-10	-1.988	8.77715E-12
CDKN1A	1.360	0.001639444	2.967	3.34471E-45
INCA1	1.985	3.1796E-06	2.403	9.04515E-10

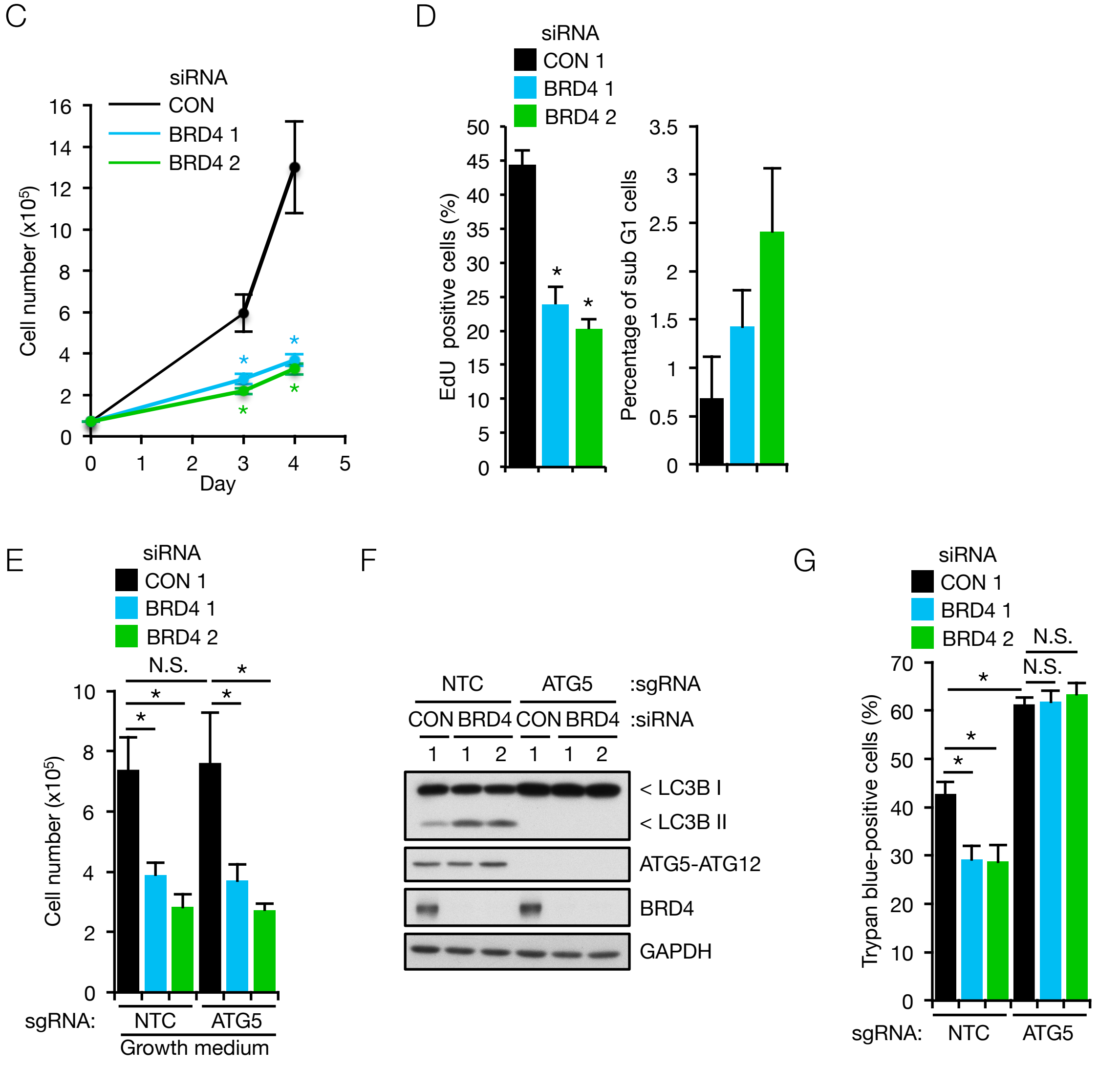

H

Gene Symbol	Fold change BRD4 1 vs Con 1	P value BRD4 1 vs Con 1	Fold change BRD4 2 vs Con 1	P value BRD4 2 vs Con 1	Protein name
SQSTM1	2.499	2.35E-22	3.014	1.77E-32	p62
OPTN	1.827	1.45E-10	1.658	1.55E-07	Optineurin
TAX1BP1	1.220	0.0256	1.261	1.69E-02	TAX1BP1
Calcoco2	-1.180	8.64E-02	-1.038	7.75E-01	NDP52
NBR1	1.215	9.58E-02	1.173	2.08E-01	NBR1
WDFY3	1.192	6.63E-01	1.092	0.838	ALFY

Figure S7, related to Figure 7. BRD4 knockdown sustains mTOR activity during starvation and confers resistance to starvation-induced cell death
(A) KP-4 cells were transfected with BRD4 siRNA for 72hrs.
(B) The results are from the RNA-Seq analysis of KP-4 cells transfected with BRD4.
(C, D) KP-4 cells were transfected with BRD4 siRNA. At 72 and 96 hrs after transfection, cell number was determined (C). At 72 hrs after transfection, EdU incorporation (D, left) assays were conducted. Measurement of subG1 cells (D, right) shows no significant increase in cell death in BRD4 knockdown cells under normal culture conditions.
(E, F) KP-4 cells infected with Cas9/ATG5 sgRNA were transfected with BRD4 siRNA. At 72 hr after transfection, cell number was determined (E). (F) shows efficient depletion of ATG5-ATG12 complex and loss of LC3II in Cas9/ATG5 sgRNA-infected cells.
(G) KP-4 cells infected with Cas9/ATG5 sgRNA were transfected with BRD4 siRNA. Following 48 hr starvation, percentage of dead cells was determined by trypan blue exclusion test.
(H) The results are from the RNA-Seq analysis of KP-4 cells transfected with BRD4 siRNA.
All data are shown as mean ± SD. B-E, G, and H: n=3 independent experiments. *$P < 0.01$, N.S.: no significance.
