# Supplementary material for: Bromodomain Protein BRD4 Is a Transcriptional Repressor of Autophagy and Lysosomal Function
Source: Mol Cell. 2017 May 18;66(4):517–532.e9. doi: 10.1016/j.molcel.2017.04.027 (PMC5446411; doi:10.1016/j.molcel.2017.04.027)
Supplement: Document S2. Article plus Supplemental Information [file mmc4.pdf]

Article

Molecular Cell

Bromodomain Protein BRD4 Is a Transcriptional Repressor of Autophagy and Lysosomal Function

Graphical Abstract

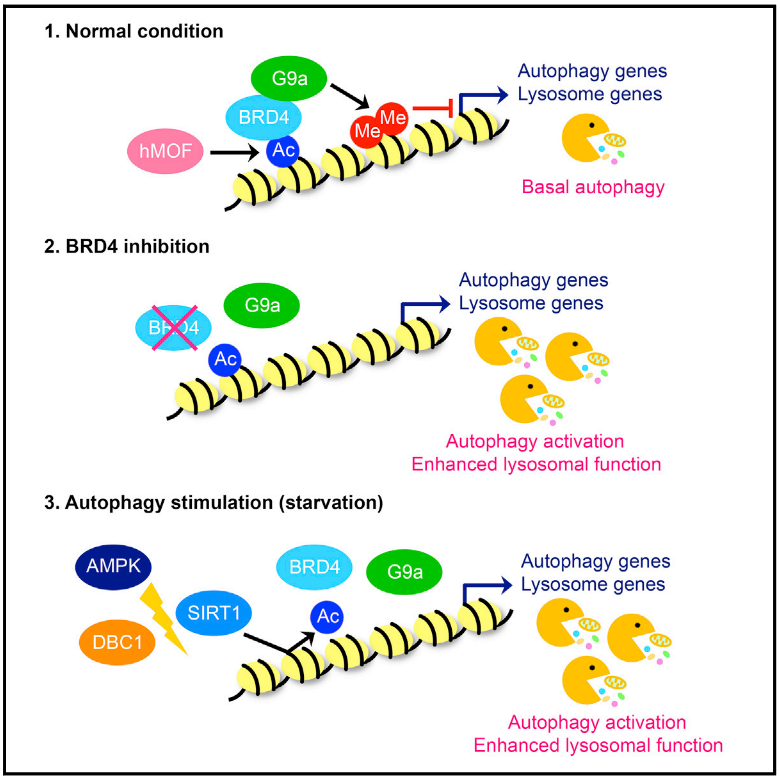

Highlights

- BRD4 represses a program of autophagy and lysosome genes independently of MiT/TFE
- BRD4 de-repression promotes certain types of autophagy, but not others
- Nutrient deprivation de-represses BRD4 via AMPK signaling to promote cell survival
- Oncoprotein BRD4-NUT is a potent repressor of autophagy and lysosome function

Authors

Jun-ichi Sakamaki, Simon Wilkinson, Marcel Hahn, ..., Scott W. Lowe, Ivan Dikic, Kevin M. Ryan

Correspondence

k.ryan@beatson.gla.ac.uk

In Brief

Sakamaki et al. show that BRD4 represses autophagy and lysosome gene expression. This repression is alleviated during nutrient deprivation through AMPK-SIRT1 signaling, allowing autophagy activation. BRD4 inhibition enhances autophagic flux and lysosomal function and promotes the degradation of protein aggregates.

Sakamaki et al., 2017, Molecular Cell *66*, 517–532
May 18, 2017 © 2017 The Authors. Published by Elsevier Inc.
http://dx.doi.org/10.1016/j.molcel.2017.04.027

Molecular Cell
Article

Bromodomain Protein BRD4 Is a Transcriptional Repressor of Autophagy and Lysosomal Function

Jun-ichi Sakamaki,[1] Simon Wilkinson,[1,8] Marcel Hahn,[2] Nilgun Tasdemir,[3] Jim O'Prey,[1] William Clark,[1] Ann Hedley,[1] Colin Nixon,[1] Jaclyn S. Long,[1] Maria New,[4] Tim Van Acker,[4] Sharon A. Tooze,[4] Scott W. Lowe,[3,5] Ivan Dikic,[2,6,7] and Kevin M. Ryan[1,9,*]
[1]Cancer Research UK Beatson Institute, Garscube Estate, Switchback Road, Glasgow G61 1BD, UK
[2]Institute of Biochemistry II, Goethe University School of Medicine, 60590 Frankfurt, Germany
[3]Department of Cancer Biology and Genetics, Memorial Sloan Kettering Cancer Center, New York, NY 10065, USA
[4]Molecular Cell Biology of Autophagy, Francis Crick Institute, London NW1 1AT, UK
[5]Howard Hughes Medical Institute, New York, NY 10065, USA
[6]Buchmann Institute for Molecular Life Sciences, Goethe University, 60438 Frankfurt, Germany
[7]Institute of Immunology, School of Medicine, University of Split, 21 000 Split, Croatia
[8]Present address: Edinburgh Cancer Research UK Centre, MRC Institute of Genetics and Molecular Medicine, University of Edinburgh, Edinburgh EH4 2XR, UK
[9]Lead Contact
*Correspondence: k.ryan@beatson.gla.ac.uk
http://dx.doi.org/10.1016/j.molcel.2017.04.027

SUMMARY

Autophagy is a membrane-trafficking process that directs degradation of cytoplasmic material in lysosomes. The process promotes cellular fidelity, and while the core machinery of autophagy is known, the mechanisms that promote and sustain autophagy are less well defined. Here we report that the epigenetic reader BRD4 and the methyltransferase G9a repress a TFEB/TFE3/MITF-independent transcriptional program that promotes autophagy and lysosome biogenesis. We show that BRD4 knockdown induces autophagy in vitro and in vivo in response to some, but not all, situations. In the case of starvation, a signaling cascade involving AMPK and histone deacetylase SIRT1 displaces chromatin-bound BRD4, instigating autophagy gene activation and cell survival. Importantly, this program is directed independently and also reciprocally to the growth-promoting properties of BRD4 and is potently repressed by BRD4-NUT, a driver of NUT midline carcinoma. These findings therefore identify a distinct and selective mechanism of autophagy regulation.

INTRODUCTION

(Macro) autophagy is a catabolic process that delivers intracellular constituents and organelles to lysosomes for degradation (Mizushima et al., 2008). This process operates at basal levels in virtually all cells and contributes to the preservation of cellular fidelity. Autophagy can also be activated by various stresses and signaling cues to promote the degradation of specific species to bring about selective desired effects within cells (Khaminets et al., 2016; Rabinowitz and White, 2010). The importance of the process is exemplified by the fact that its dysregulation is implicated in various diseases, including neuronal degeneration, immune diseases, and cancer (Mizushima et al., 2008).

Autophagy is initiated by the formation of double-membraned structures called phagophores that originate from endoplasmic reticulum (ER)-derived omegasomes as well as other sources (Ktistakis and Tooze, 2016). As phagophores grow, they form sphere-like structures called autophagosomes that sequester and entrap cytoplasmic components. Autophagosomes can then fuse with other organelles, such as endosomes, but ultimately, fusion occurs with lysosomes forming autolysosomes within which cargo digestion occurs (Ktistakis and Tooze, 2016). Intensive studies have identified the genes involved in the various steps of autophagy, which has led to an established basic machinery for this complicated vesicular trafficking system (Ktistakis and Tooze, 2016; Lamb et al., 2013).

Numerous signaling pathways that regulate autophagy in response to specific stimuli have been identified (Lamb et al., 2013). Recent accumulating evidence has also highlighted the importance of transcriptional regulation of autophagy to sustain prolonged autophagy and/or maintain basal autophagy (Baek and Kim, 2017; Füllgrabe et al., 2014, 2016). The precise control of suppression and de-repression of autophagy is essential as both excess and insufficient autophagy activation has been shown to be deleterious to cells (Mizushima et al., 2008). However, the detailed regulatory mechanisms controlling autophagy in both general and specific contexts remain largely unknown.

Successful completion of autophagy also requires functional lysosomes, acidic organelles that contain various acid hydrolases for the degradation of macromolecules (Shen and Mizushima, 2014). Lysosomal dysfunction impairs the degradation of autophagic cargo, as well as molecules delivered by the

 © 2017 The Authors. Published by Elsevier Inc.
This is an open access article under the CC BY license (http://creativecommons.org/licenses/by/4.0/).

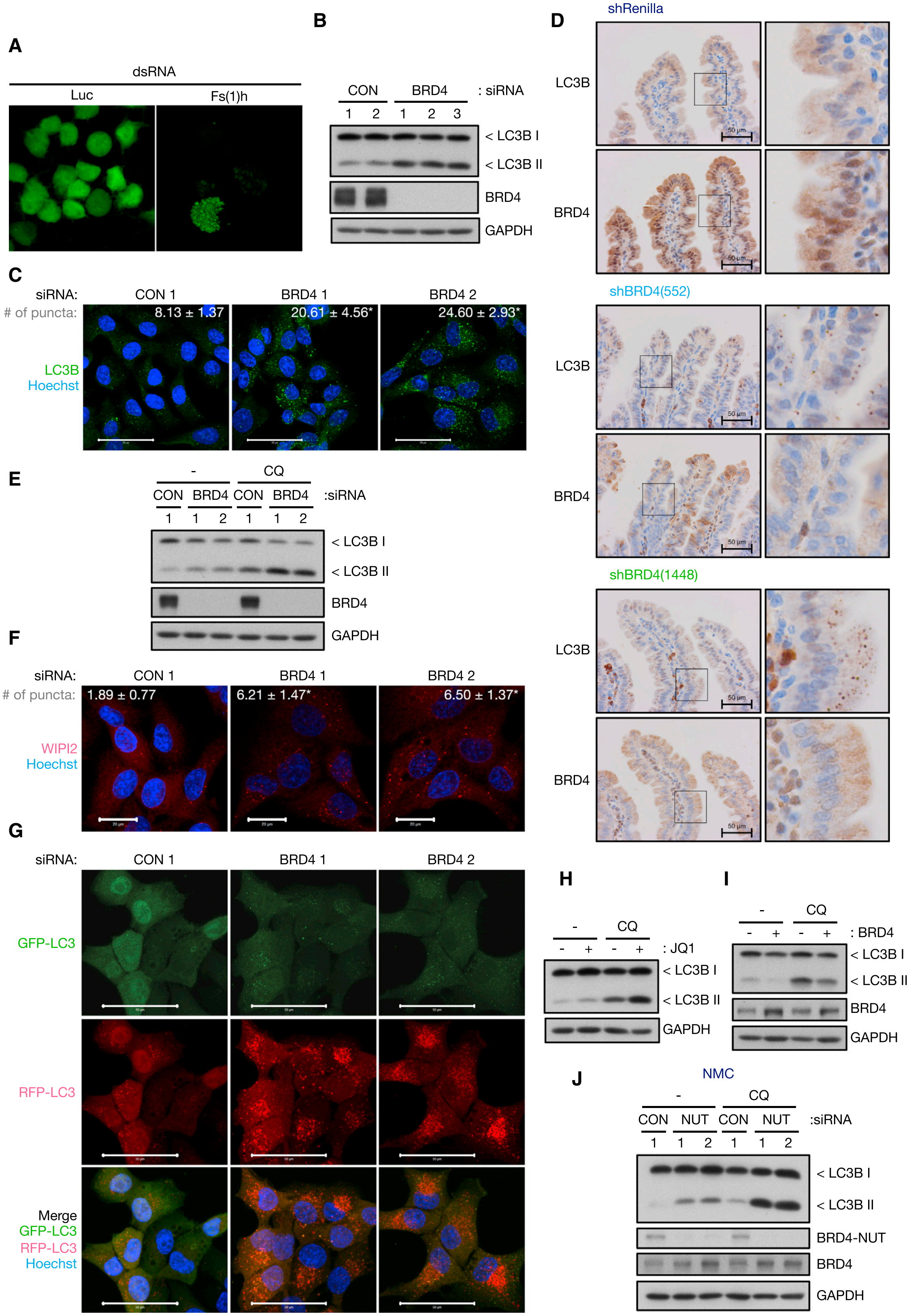

(legend on next page)

endocytic pathway, macropinocytosis, and chaperone-mediated autophagy, which in turn causes diseases, including neuronal degeneration and lysosomal storage disorders (Shen and Mizushima, 2014). In the course of an increased autophagic response, lysosome biogenesis and function must also be enhanced to support increased cargo degradation, yet the mechanism underlying this effect is poorly understood. Recent reports have shown that the coordinated activation of the autophagy-lysosome pathway is governed by several transcription factors, including transcription factor EB (TFEB) (Settembre et al., 2011). Following certain autophagic stimuli, TFEB translocates to the nucleus and activates a subset of autophagy and lysosome genes. This enhances autophagosome formation, their fusion with lysosomes, and lysosome biogenesis and function (Settembre et al., 2011).

Bromodomain-containing protein 4 (BRD4) is a member of the bromodomain and extraterminal (BET) family proteins characterized by two N-terminal bromodomains and an extraterminal (ET) domain (Shi and Vakoc, 2014). BRD4 binds to acetylated histones and transcription factors through bromodomains and recruits transcriptional regulators such as positive transcription elongation factor b (P-TEFb) and Mediator complex (Shi and Vakoc, 2014). BRD4 is involved in the activation of genes involved in cell growth and cell cycle progression (Wang and Filippakopoulos, 2015). As a result, intensive studies have been focused on the role of BRD4 in cancer, and BET inhibitors have been proven to have efficacy against various types of tumors (Wang and Filippakopoulos, 2015). Intriguingly, recent accumulating evidence has shown that BRD4 also plays a role in different biological processes, including memory formation, mitochondrial oxidative phosphorylation, and DNA damage response (Barrow et al., 2016; Floyd et al., 2013; Korb et al., 2015). However, our understanding of the biological role of BRD4 requires further investigation.

Here, by using RNAi screening and transcriptome analysis, we have identified BRD4 as a transcriptional repressor of autophagy and lysosomal function. We show that BRD4 suppresses the expression of a subset of autophagy and lysosome genes by binding to the promoter regions under normal growth conditions and that this repression is alleviated in response to certain autophagic stimuli. Inhibition of BRD4 enhances autophagic flux and lysosomal function, which consequently promotes the degradation of pathogenic protein aggregates and confers the resistance to starvation-induced cell death. These observations therefore provide important insights into a regulatory mechanism controlling autophagy and lysosome function.

RESULTS

BRD4 Is a Repressor of Autophagy

To understand the regulatory mechanisms of autophagy, we conducted an RNAi screen using *Drosophila* $S2R^+$ cells stably expressing GFP-LC3 (Wilkinson et al., 2011). Double-stranded RNA targeting female sterile (1) homeotic (Fs(1)h) was one of the hits that increased GFP-LC3 puncta (Figure 1A). Fs(1)h is a BET protein that functions as a scaffold protein bridging acetylated histones and transcriptional regulators (Kellner et al., 2013). The mammalian BET family consists of four members: ubiquitously expressed BRD2, BRD3, and BRD4 and testis-specific BRDT (Shi and Vakoc, 2014). To validate the screening results, we knocked down the genes encoding BRD2, BRD3, or BRD4 in human pancreatic ductal adenocarcinoma KP-4 cells and determined their effects on autophagy by monitoring the levels of the lipidated form of LC3 (LC3II)—a marker of autophagosome formation/accumulation (Klionsky et al., 2016). This revealed that knockdown of BRD4, but not BRD2 and BRD3, led to an increase in LC3II levels (Figure 1B; Figures S1A and S1B). The generality of this finding was confirmed using a panel of different cell lines (Figure S1C). Consistent with LC3II accumulation, the number of LC3 puncta, an indicator of autophagosome formation (Klionsky et al., 2016), was also increased in BRD4 knockdown cells (Figure 1C). Furthermore, analysis of intestinal sections from mice expressing an inducible BRD4 shRNA revealed that LC3 lipidation and puncta also increased in vivo upon knockdown of BRD4 (Figure 1D; Figure S1D).

There are three BRD4 isoforms reported—isoform A (referred to as long isoform) that possesses a carboxy-terminal domain (CTD) containing the binding site for P-TEFb, isoform B that lacks the CTD and has a unique 77 amino acid extension at its C terminus, and isoform C (referred to as short isoform) that is the shortest isoform lacking the CTD (Figure S1E). Isoform-specific function of BRD4 has been described (Floyd et al., 2013). Knockdown of either the short or the long isoform of BRD4 had no effect on LC3II, while simultaneous depletion of both isoforms promoted LC3 lipidation (Figures S1F and S1G), indicating that BRD4 short and long isoforms are functionally redundant in the regulation of autophagy. Of note, we could not detect BRD4 isoform B in KP-4 cells.

Figure 1. BRD4 Silencing Enhances Autophagic Flux

(A) *Drosophila* $S2R^+$ cells expressing GFP-LC3 were transfected with double-stranded RNA (dsRNA) targeting control luciferase (Luc) or Fs(1)h.

(B and C) KP-4 cells transfected with control or BRD4 siRNA for 72 hr were subjected to western blot analysis (B) and stained for LC3B (C). The number of LC3 puncta normalized to cell number is shown. CON: n = 94 cells, BRD4 1: n = 97 cells, BRD4 2: n = 74 cells. Scale bars, 50 μm.

(D) Immunohistochemistry of small intestinal sections from transgenic mice harboring inducible renilla luciferase or BRD4 shRNA. Sections were stained for LC3 (upper) and BRD4 (lower). Cytoplasmic signal in BRD4 panels is due to non-specific staining. Scale bars, 50 μm.

(E) KP-4 cells transfected with BRD4 siRNA were treated with 10 μM CQ for 4 hr.

(F) KP-4 cells transfected with BRD4 siRNA were stained for WIPI2. The number of WIPI2 puncta normalized to cell number is shown. CON: n = 119 cells, BRD4 1: n = 107 cells, BRD4 2: n = 109 cells. Scale bars, 20 μm.

(G) KP-4 cells stably expressing RFP-GFP-LC3 were transfected with BRD4 siRNA. Scale bars, 50 μm.

(H) KP-4 cells were treated with 500 nM JQ1 for 9 hr in the presence or absence of CQ (10 μM, 4 hr).

(I) KP-4 cells overexpressing BRD4 were treated with 10 μM CQ for 4 hr.

(J) TY-82 cells transfected with NUT siRNA for 5 days were treated with 10 μM CQ for 8 hr. BRD4-NUT was detected using NUT antibody.

All data are shown as mean ± SD. $^*p < 0.01$. See also Figure S1.

As LC3II accumulation is attributed to either increased autophagy induction or impaired autophagosome turnover, the effect of BRD4 knockdown on autophagic flux was examined in the presence of chloroquine (CQ), an inhibitor of lysosomal degradation (Klionsky et al., 2016). As shown in Figures 1E, S1H, and S1I, BRD4 silencing increased LC3II levels in the presence of CQ, suggesting that BRD4 knockdown enhances autophagic flux.

To further examine the stage at which BRD4 affects autophagy, we first examined the recruitment of WD repeat domain phosphoinositide interacting 2 (WIPI2) to phosphatidylinositol 3-phosphate (PI3P)-enriched membrane—an event that precedes LC3 lipidation and which is used as a marker of early stages of autophagy induction (Klionsky et al., 2016). This revealed that an increased number of WIPI2 puncta were also observed in BRD4-silenced cells (Figure 1F). In addition, we performed a detailed examination of LC3 localization by using RFP-GFP-tandem-tagged LC3 (Kimura et al., 2007). Due to the acid lability of GFP in (auto)lysosomes, this revealed an increase in GFP^{-}/RFP^{+} autolysosomes and also, to lesser extent, GFP^{+}/RFP^{+} phagophores/autophagosomes in BRD4 knockdown cells (Figure 1G), suggesting that BRD4 knockdown promotes the formation of autophagosomes and subsequent fusion with lysosomes. Autophagy receptors, such as p62/SQSTM1, are degraded together with cargos and are used as readouts of autophagic degradation (Klionsky et al., 2016). Consistently, BRD4 silencing led to a reduction in exogenously expressed GFP-p62 levels, and this p62 degradation was blocked by CQ (Figure S1J).

BET inhibitors displace BRD proteins from promoter and enhancer regions, thereby interfering with BRD-mediated transcriptional regulation (Shi and Vakoc, 2014). Similar to the results we obtained in BRD4 knockdown cells, BET inhibitor JQ1 (Filippakopoulos et al., 2010) increased LC3II levels (Figure 1H; Figures S1K and S1L). As this did not occur in the absence of BRD4 (Figure S1M), we conclude that autophagy activation by JQ1 is attributed to BRD4 inhibition. In addition, increased LC3 lipidation and puncta formation were observed in mice treated with JQ1 (Figures S1N and S1O). Similarly, we found that the BET degrader ARV-825 (Lu et al., 2015) also activates autophagy (Figure S1P). Conversely, overexpression of BRD4 suppressed autophagic flux (Figure 1I; Figure S1Q). Collectively, these results identify BRD4 as a conserved negative regulator of autophagy.

Chromosomal translocation of *BRD4* to the locus encoding nuclear protein in testis (NUT) causes NUT midline carcinoma (NMC), a rare aggressive subtype of squamous cell carcinoma (French, 2010). The fusion gene product BRD4-NUT possesses two N-terminal bromodomains, an ET domain, and almost the full length of NUT at its C terminus (Figure S1E) (French, 2010). As a result, we were interested to know whether BRD4-NUT also functions as a suppressor of autophagy. By taking advantage of the testis-specific expression of NUT, we used small interfering RNA (siRNA) against NUT to knockdown BRD4-NUT (Schwartz et al., 2011). Inhibition of BRD4-NUT by NUT siRNAs or JQ1 caused accumulation of LC3II in the presence of CQ in the TY-82 NMC cell line (Figure 1J; Figures S1R and S1S), and the effect of JQ1 treatment on LC3 lipidation was comparable to that of BRD4-NUT knockdown (Figure S1T). BRD4-NUT knockdown also increased the formation of GFP^{-}/RFP^{+} LC3 puncta, indicating an accumulation of autolysosomes (Figure S1U). Interestingly, knockdown of BRD4 expressed from the unaffected allele had little effect on autophagy compared to BRD4-NUT knockdown (Figure S1V), suggesting that BRD4-NUT fusion protein is a dominant repressor of autophagy in NMC.

BRD4 Is a Negative Regulator of Autophagy Gene Expression

As BRD4 is a transcriptional regulator, we hypothesized that BRD4 regulates autophagy at the transcriptional level. RNA sequencing (RNA-seq) analysis followed by reverse transcriptase quantitative PCR (RT-qPCR) validation revealed that a significant number of autophagy genes were upregulated upon knockdown of BRD4 (Figures 2A and 2B; Figures S2A–S2D). These include genes that encode proteins involved in autophagosome formation (BECN1, VMP1, PIK3C3, WIPI1, ATG2A, ATG9B, and MAP1LC3B) (Lamb et al., 2013), autophagy cargo recruitment (SQSTM1 and OPTN), autophagosome-lysosome fusion (PLEKHM1, TECPR1, and HOPS complex components) (McEwan et al., 2015a), and maintenance of functional ER exit sites and autophagosome formation (MAP1LC3C, TECPR2, and SEC24D) (Stadel et al., 2015). BET inhibitors also led to upregulation of autophagy genes (Figure 2C). Of note, de-repression of autophagy genes was observed almost immediately after JQ1 addition (Figure 2D), implying that these autophagy genes are directly regulated by BRD4. In addition, we found that overexpression of BRD4 repressed autophagy gene expression (Figure 2E; Figure S2E). As it is well established that BRD4 can form a complex with P-TEFb and facilitate productive elongation at promoter-proximal regions (Shi and Vakoc, 2014), we considered that the effect on autophagy may also be through this mechanism. We found, however, that knockdown of cyclin-dependent kinase 9 (CDK9), a subunit of P-TEFb, had no effect on LC3II levels (Figures S2F and S2G). This rules out the involvement of P-TEFb in this response and indicates that BRD4 modulates autophagy through a distinct pathway.

BRD4 Regulates Lysosome Gene Expression and Lysosomal Function

As significant changes in lysosome gene expression occur upon BRD4 knockdown (Figure 2A), this prompted us to examine whether these alterations enhance lysosomal function and support increased autophagic flux. First, we validated the RNA-seq results by conducting RT-qPCR analyses, which showed that BRD4 knockdown significantly upregulated a number of lysosome genes involved in proteolysis, glycan degradation, and lysosome biogenesis (Figure 3A). Consistent with this, we observed an increase in lysosomal protein levels, including lysosomal-associated membrane protein 1 (LAMP1), LAMP2, acid sphingomyelinase (ASM), α-glucosidase (GAA), and heavy chain of mature cathepsin B (CTSB HC) and cathepsin D (CTSD HC) (Figure 3B; Figure S3A). Staining of lysosomal compartments with anti-LAMP1 antibody and LysoTracker red also revealed an expanded lysosomal area in BRD4 knockdown cells (Figures 3C and 3D, upper panels). To assess the activity of lysosomal enzymes, we employed the use of Magic Red CTSB, a CTSB substrate that produces a cresyl violet fluorophore upon proteolytic

A

GO term	GO ID	P value	Gene #
Lysosome	GO:0005764	3.00E-012	53
Lysosomal lumen	GO:0043202	3.34E-012	22
Endosome	GO:0005768	4.77E-005	53
Autophagy	GO:0006914	2.65E-004	31
Regulation of autophagy	GO:0010506	2.65E-002	22

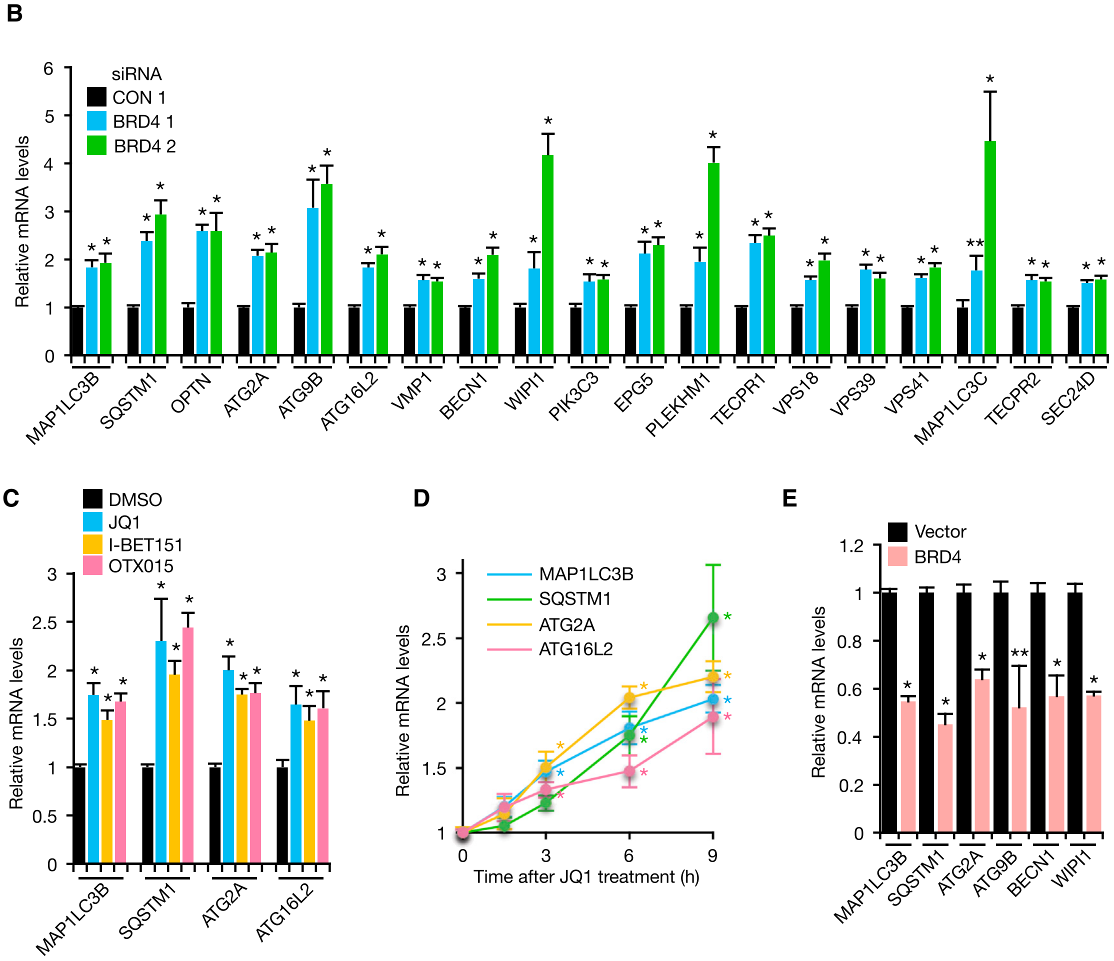

Figure 2. BRD4 Is a Negative Regulator of Autophagy Gene Expression

(A and B) KP-4 cells transfected with control or BRD4 siRNA were subjected to RNA-seq and gene ontology analyses (A) and RT-qPCR analysis (B).

(C) RT-qPCR analysis of KP-4 cells treated with DMSO, 500 nM JQ1, 500 nM I-BET151, or 500 nM OTX015 for 9 hr.

(D) RT-qPCR analysis of KP-4 cells treated with 500 nM JQ1 for the indicated time.

(E) RT-qPCR analysis of KP-4 cells overexpressing BRD4.

All data are shown as mean ± SD. In (A)–(D), n = 3 independent experiments; in (E), data are representative of two independent experiments performed in triplicate. *$p < 0.01$, **$p < 0.05$. See also Figure S2.

cleavage. As shown in Figure 3D's lower panels, a significant increase in CTSB substrate cleavage was seen in BRD4 knockdown cells. In addition, we also observed increased enzymatic activity of β-hexosaminidase, a lysosomal enzyme that catalyzes the hydrolysis of ganglioside monosialic 2, in BRD4 knockdown cells (Figure 3E). These results indicate that not only autophagic flux, but also lysosomal biogenesis and function are enhanced by BRD4 knockdown. Furthermore, we also observed upregulation of autophagy and lysosomal gene expression upon BRD4-NUT inhibition by NUT siRNA and JQ1 and an increase in lysosomal protein levels and LysoTracker^{+} acidic compartments in BRD4-NUT knockdown NMC cells (Figures 3F and 3G; Figures S3B and S3C), suggesting that BRD4-NUT also suppresses the autophagy-lysosome pathway in NMC.

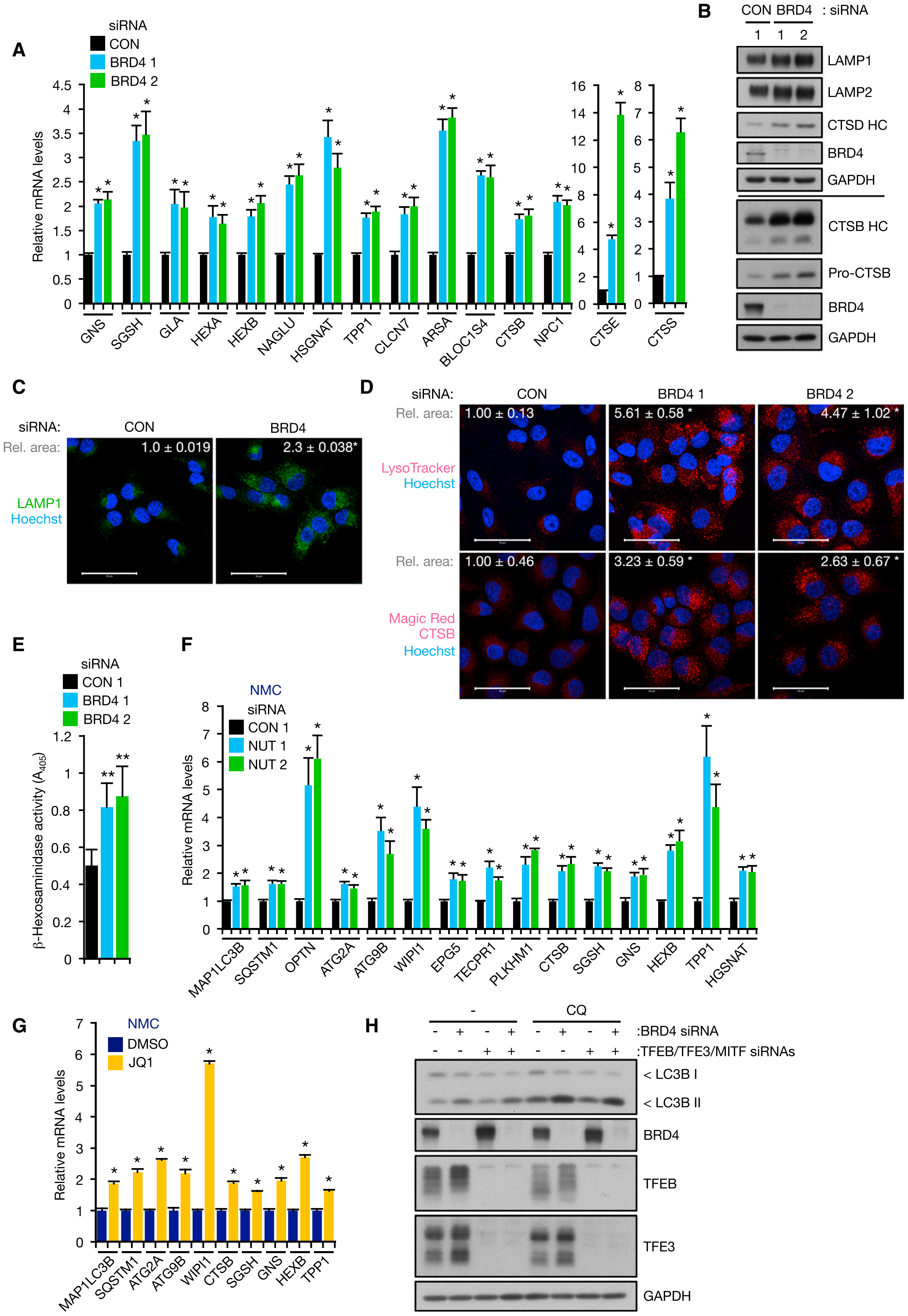

(legend on next page)

Activation of the autophagy-lysosome pathway in BRD4 knockdown cells is reminiscent of the phenotype observed by activation of TFEB (Settembre et al., 2011). We therefore tested the possibility that BRD4 knockdown stimulates autophagy through activation of TFEB and its related members TFE3 and MITF. However, BRD4 knockdown cells were capable of activating autophagy in the absence of TFEB (Figures S3D–S3F). Furthermore, BRD4 knockdown still activated autophagy and lysosome gene transcription and enhanced autophagic flux in cells where all MiT/TFE members (TFEB, TFE3, and MITF) were simultaneously silenced (Figure 3H; Figures S3G–S3J), indicating that BRD4 regulates autophagy independently of MiT/TFE transcription factors.

BRD4 Is Recruited to Autophagy Gene Promoters and Its Dissociation Leads to Transcriptional Activation of Autophagy Genes

We next examined the molecular mechanism by which BRD4 represses autophagy and lysosome gene expression. Given that BET inhibitor dissociates BRD4 from acetylated histones and rapidly upregulates autophagy genes (Figure 2D), we hypothesized that BRD4 binds to acetylated histones at autophagy and lysosome gene promoter regions. Indeed, BRD4 was found at autophagy and lysosome gene promoter regions, and its enrichment was reduced after JQ1 treatment (Figures S4A and S4B). BRD4 occupation at autophagy and lysosome gene promoters was also significantly decreased during starvation, and this BRD4 dissociation was correlated with upregulation of gene expression (Figures 4A and 4B).

Interestingly, histone H4 lysine 16 (H4K16) acetylation that is recognized by BRD4 (Zippo et al., 2009) is downregulated upon autophagic stimulation (Füllgrabe et al., 2013). H4K16 acetylation and its acetyltransferase human males absent on the first (hMOF) have been described as both positive and negative regulators of autophagy (Füllgrabe et al., 2013; Hale et al., 2016). Therefore, we tested whether BRD4 recruitment is regulated by hMOF. CRISPR/CRISPR-associated protein 9 (Cas9)-mediated editing of *hMOF* efficiently reduced hMOF protein levels and H4K16Ac status at autophagy gene promoters (Figures S4C and S4D). Depletion of hMOF also caused BRD4 dissociation from autophagy gene promoters, upregulation of autophagy gene expression, and increased LC3 lipidation (Figure 4C; Figures S4E and S4F). Consistent with the previous study (Füllgrabe et al., 2013), H4K16Ac declined upon starvation (Figure 4D), implying that BRD4 dissociation is attributed to H4K16 deacetylation in response to starvation. However, analysis of hMOF protein levels revealed that they did not change during starvation (Figure S4G). As a result, we hypothesized instead that the decrease in H4K16 acetylation and subsequent BRD4 dissociation during starvation may be driven by the deacetylase Sirtuin 1 (SIRT1) (Vaquero et al., 2007). In line with this hypothesis, H4K16 deacetylation and BRD4 dissociation by starvation were not seen in cells infected with Cas9/single-guide RNA (sgRNA) targeting SIRT1 (Figure 4E; Figure S4H). In addition, SIRT1 depletion suppressed starvation-induced autophagy gene expression and LC3 lipidation (Figure 4F; Figure S4I), underscoring the importance of this enzyme in this response.

It is known that SIRT1 is activated by nutrient deprivation via its dissociation from the inhibitory molecule Deleted in Breast Cancer protein 1 (DBC1) and an increase in nicotinamide adenine dinucleotide (NAD^+) levels in a manner dependent on AMP-activated protein kinase (AMPK) (Cantó et al., 2009; Chang et al., 2015). As expected, AMPK phosphorylation at Thr172, which is required for its activation, increased during nutrient starvation (Figure S4J). Consistent with these observations, we found that BRD4 forms a complex with SIRT1 and DBC1 and that starvation disrupts the SIRT1-DBC1 interaction (Figures S4K and S4L). The dissociation of this interaction was not observed in cells treated with AMPK inhibitor Compound C and infected with Cas9/sgRNAs targeting AMPKα catalytic subunits (Figure 4G; Figure S4M). Moreover, disruption of *AMPK*α genes prevented BRD4 dissociation from autophagy gene promoters in response to starvation (Figure S4N). As a consequence, AMPK inhibition blocked autophagy gene expression and autophagic flux induced by starvation (Figure 4H; Figures S4O and S4P). Collectively, these results detail a signaling cascade from nutrient deprivation to de-repression of autophagy gene transcription, which involves the disruption of SIRT1-DBC1 interaction by AMPK and SIRT1-mediated BRD4 dissociation from autophagy gene promoters.

BRD4 Represses Autophagy Gene Expression through Binding to G9a

In contrast to its well-established role as a positive transcriptional regulator of genes involved in cell growth (Shi and Vakoc, 2014), BRD4 represses expression of a subset of autophagy genes. We therefore investigated the molecular mechanism by which BRD4 suppresses autophagy gene expression. From previous interactome analyses of BRD4 (Dawson et al., 2011), we searched for BRD4 interacting protein(s) that are known to be involved in gene repression. Different from the majority of interacting proteins, histone lysine methyltransferase G9a has been shown to both promote and repress transcription by catalyzing mono- and di-methylation of histone H3 at lysine 9 (H3K9),

Figure 3. BRD4 Knockdown Enhances Lysosomal Function

(A) RT-qPCR analysis of KP-4 cells transfected with control or BRD4 siRNA.

(B–D) KP-4 cells transfected with BRD4 siRNA were subjected to western blot analysis with antibodies against lysosomal proteins (B) and stained with LAMP1 antibody (C), LysoTracker Red (100 nM, 2 hr) (D, upper panels), and Magic Red CTSB (1 hr) (D, lower panels). Area of $LAMP1^+$, $LysoTracker^+$, and Magic Red $CTSB^+$ area normalized to cell number is shown (C, CON: n = 115 cells, BRD4: n = 130 cells; D upper, CON: n = 66 cells, BRD4 1: n = 52 cells, BRD4 2: n = 50 cells; D lower, CON: n = 164 cells, BRD4 1: n = 109 cells, BRD4 2: n = 53 cells). Scale bars, 50 μm.

(E) Hexosaminidase activity was measured using lysates from control and BRD4 knockdown KP-4 cells.

(F and G) RT-qPCR analysis of TY-82 cells transfected with NUT siRNA for 72 hr (F) or treated with 500 nM JQ1 for 9 hr (G).

(H) KP-4 cells were transfected with BRD4 and/or MiT/TFE (TFEB, TFE3, MITF) siRNAs and treated with 10 μM CQ for 4 hr.

All data are shown as mean ± SD. In (A) and (F), n = 3 independent experiments. In (E), n = 4 independent experiments. In (G), data are representative of two independent experiments performed in triplicate. *$p < 0.01$, **$p < 0.05$. See also Figure S3.

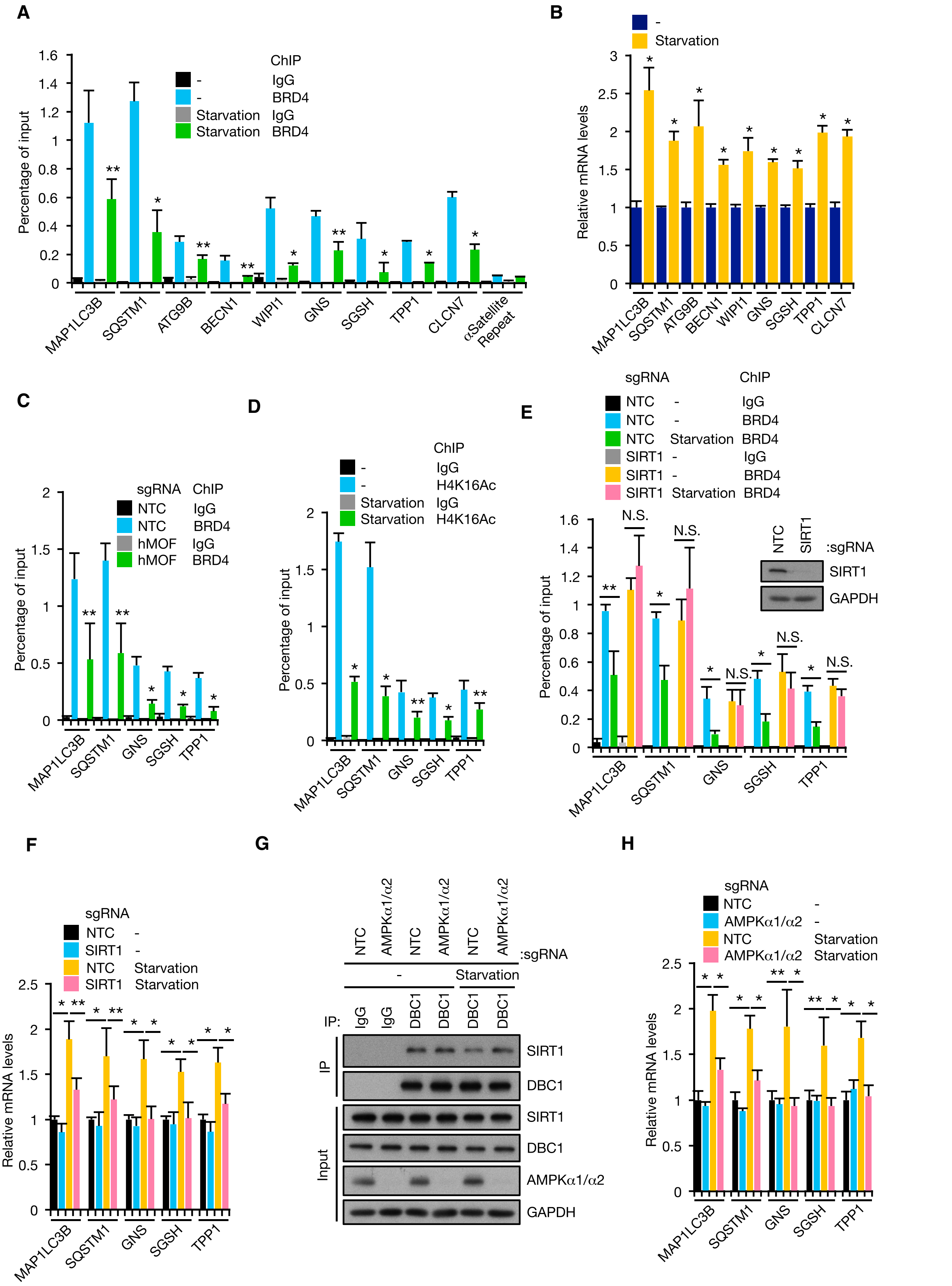

(legend on next page)

respectively (Shinkai and Tachibana, 2011). We first confirmed the interaction between BRD4 and G9a by reciprocal co-immunoprecipitation (Figure 5A). G9a interacted with the long and short isoforms of BRD4 (Figure S5A), which is consistent with our observations that both isoforms contribute to autophagy repression (Figure S1F). Importantly, we observed that the interaction between BRD4 and G9a was disrupted by starvation (Figure 5B). In addition, G9a recruitment to autophagy gene promoters was abolished by JQ1 treatment and BRD4 knockdown (Figures 5C and 5D; Figures S5B and S5C). Consequently, H3K9diMe status was also reduced in these settings (Figures 5E and 5F). Consistent with a functional role in the regulation of autophagy, G9a silencing upregulated autophagy gene expression and enhanced autophagic flux (Figures 5G and 5H; Figures S5D and S5E). Furthermore, simultaneous knockdown of BRD4 and G9a did not cause further accumulation of LC3II and autophagy gene transcripts (Figures 5G and 5H; Figure S5E), and G9a silencing largely abolished autophagy suppression by BRD4 overexpression (Figure 5I; Figure S5F), suggesting that BRD4 and G9a act on the same pathway. In addition, we found that autophagy regulation by H4K16 acetylation and G9a is also conserved in NMC. Depletion of hMOF decreased H4K16 acetylation and increased LC3II levels in TY-82 cells (Figures S5G–S5I). Starvation led to a SIRT1-dependent H4K16 deacetylation at autophagy gene promoters (Figure S5J). BRD4-NUT interacts with G9a, and G9a silencing promoted LC3 lipidation (Figures S5K and S6L), implying that, like BRD4, BRD4-NUT suppresses autophagy through G9a.

BRD4 Knockdown Modulates Specific Types of Autophagy

Bulk degradation of cytoplasmic components by autophagy, termed bulk autophagy, is thought to feed energy sources during nutrient shortage, whereas degradation of specific substrates, such as protein aggregates, damaged mitochondria and pathogens, called selective autophagy, serves as an intracellular quality control mechanism (Khaminets et al., 2016; Rabinowitz and White, 2010). We were therefore interested in understanding the contribution of BRD4 to the control of stimulus-dependent and selective autophagy.

In the first instance, we examined starvation- and rapamycin-induced autophagy and found that they were augmented by BRD4 silencing (Figures 6A and 6B; Figures S6A and S6B). Reduction of both LC3I and II after 2 hr of starvation in BRD4 knockdown cells suggests enhanced LC3I conversion to LC3II and subsequent degradation (Figure 6A; Figure S6A). Conversely, BRD4 overexpression suppressed LC3I conversion to LC3II induced by starvation and rapamycin (Figure S6C). In addition, BRD4 knockdown further activated autophagy induced by glucose starvation, hypoxia, trehalose, and oncogenic Ras mutant (Figures 6C–6F; Figures S6D–S6G). Similarly, TFEB and TFE3-mediated autophagy activation was also augmented by BRD4 and G9a silencing (Figures S6H and S6I). We next determined whether BRD4 knockdown would promote the autophagic degradation of protein aggregates (aggrephagy). To test this, we analyzed aggregates caused by mutant Huntingtin (HTT) and found that BRD4 silencing promoted the degradation of poly-glutamine (Q)-expanded HTT (HTT 94Q) (Figure 6G). Conversely, BRD4 overexpression exacerbated the accumulation of HTT 94Q in the insoluble fraction (Figure S6J). Similar to the previous reports (Williams et al., 2008), induction of polyQ-expanded HTT caused a reduction in cell number, and BRD4 knockdown ameliorated this effect (Figure S6K).

In contrast, modulation of BRD4 did not promote or prevent the clearance of *Salmonella enterica* serovar Typhimurium by xenophagy (Figure 6H; Figure S6L) or mitochondria by mitophagy (Figure 6I; Figures S6M and S6N). In fact, we actually observed an accumulation of mitochondrial proteins in BRD4 knockdown cells, which may be due to transcriptional upregulation of mitochondrial genes as recently described (Barrow et al., 2016). A similar effect was also observed with p62/SQSTM1 mRNA and protein levels upon BRD4 knockdown (Figure 2B; Figure S6O), thereby complicating its utility as a marker of autophagic activity modulated by BRD4. In conclusion, our collective results show that BRD4 knockdown affects some, but not all, types of autophagy, indicating that it is a regulator of this response in specific contexts.

BRD4 Knockdown Sustains mTOR Activity during Starvation and Confers Resistance to Starvation-Induced Cell Death

Autophagic degradation of intracellular proteins produces amino acids, leading to activation of the amino acid sensor mechanistic target of rapamycin complex1 (mTORC1), and these nutrient sources can be used to maintain cell survival during periods of starvation (Perera et al., 2015; Rabinowitz and White, 2010). We observed that BRD4 knockdown sustained the phosphorylation status of S6K, a substrate of mTORC1 and established readout of mTORC1 activity (Perera et al., 2015), during amino acid starvation (Figure 7A). This sustained S6K phosphorylation was abolished by CQ and CRISPR/Cas9-mediated ATG5 gene disruption (Figures 7B and 7C), suggesting that BRD4 knockdown activates mTORC1 through the autophagy-lysosome pathway during amino acid shortage. Therefore, we next examined whether autophagy activation by BRD4 knockdown affects cell growth and cell survival during nutrient deprivation. As reported previously (Shi and Vakoc, 2014; Zuber et al.,

Figure 4. Starvation Leads to BRD4 Dissociation from Autophagy Gene Promoters

(A and B) KP-4 cells were starved for 4 hr followed by chromatin immunoprecipitation (ChIP) assay with BRD4 antibody (A) and RT-qPCR analysis (B).

(C) KP-4 cells infected with Cas9/hMOF sgRNA were subjected to ChIP assay with BRD4 antibody.

(D) KP-4 cells were starved for 4 hr followed by ChIP assay with H4K16Ac antibody.

(E and F) KP-4 cells infected with Cas9/SIRT1 sgRNA were starved for 4 hr followed by ChIP assay with BRD4 antibody (E) and RT-qPCR analysis (F). Western blot shows efficient SIRT1 depletion in Cas9/SIRT1 sgRNA-infected cells.

(G and H) KP-4 cells infected with Cas9/AMPKα1 and α2 sgRNAs were starved for 4 hr followed by immunoprecipitation with DBC1 antibody (G) and RT-qPCR analysis (H).

All data are shown as mean ± SD. In (A)–(F) and (H), n = 3 independent experiments. *$p < 0.01$, **$p < 0.05$, N.S., no significance. See also Figure S4.

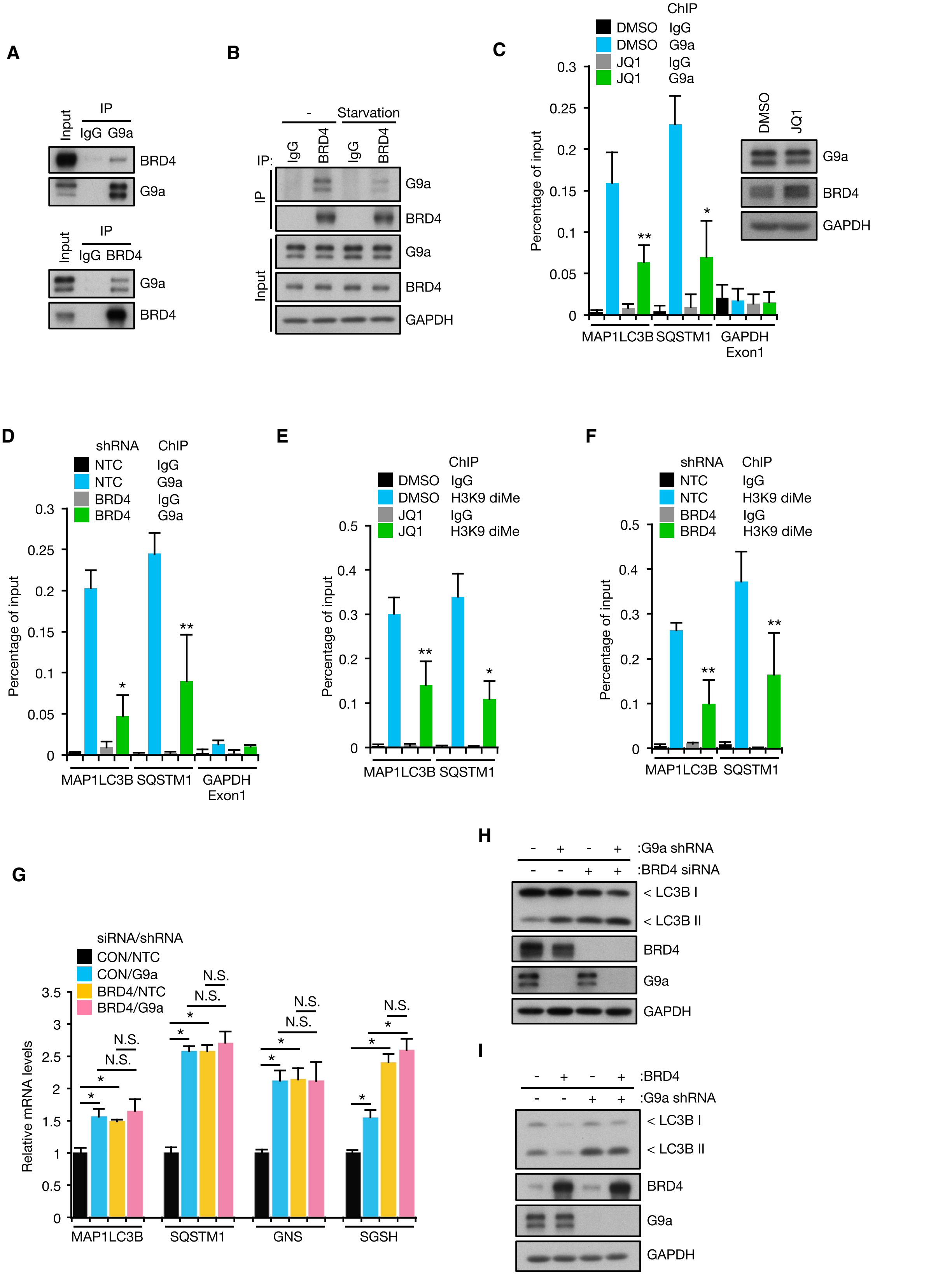

(legend on next page)

2011), BRD4 knockdown caused downregulation of c-Myc, altered cell cycle gene expression, and decreased cell proliferation due to cell cycle retardation under nutrient-replete conditions (Figures S7A–S7D). We found, however, that this growth retardation was caused independently of autophagic activity (Figures S7E and S7F). In contrast to these effects on cell growth, we found that starvation-induced cell death was significantly suppressed in BRD4 knockdown cells, and this protective effect was abolished by ATG5 gene disruption and CQ treatment (Figures 7D–7G; Figure S7G). These data therefore suggest that the modulation of autophagy by BRD4 inhibition maintains cell survival under starvation conditions by providing nutrient source.

DISCUSSION

In this study, we show that a series of autophagic processes, including autophagosome formation, fusion of autophagosomes with lysosomes, and lysosome biogenesis and function, is transcriptionally repressed by BRD4. BRD4 knockdown upregulates a subset of autophagy and lysosome genes, which in turn enhances autophagic flux and lysosome biogenesis and activity. Interestingly, the effect of this transcriptional program only affects certain forms of autophagy. We found that knockdown of BRD4 promoted autophagy induced by stimuli, such as nutrient deprivation, rapamycin, and protein aggregates, but this did not affect the autophagic removal of mitochondria or bacteria. This indicates that the program is selectively engaged, adding another layer of complexity to the control of this ubiquitous and yet multifaceted process.

As different types of selective autophagy utilize their designated receptors to recruit the cargos, we examined whether the aggrephagy receptor(s) are specifically upregulated by BRD4 and found that the expression of p62 and Optineurin increased upon BRD4 knockdown (Figure 2B; Figure S7H). However, these receptors capture ubiquitinated mitochondria and pathogens, as well as protein aggregates (Khaminets et al., 2016); therefore, these alterations do not provide a mechanistic explanation for the autophagy specificity conferred by BRD4 knockdown. Since mechanisms of selective autophagy are not fully elucidated, the future identification of selective autophagy-specific regulators will help solve this question.

In the case of autophagy induced by nutrient availability, it is already clear that the process is orchestrated by different mechanisms. The acute response to nutrient deprivation includes nutrient sensing by mTORC1 and AMPK and activation of the UNC-51-like kinase (ULK) and class III phosphatidylinositol-3 kinase (PI3K) complexes. This subsequently leads to the formation of a PI3P-enriched membrane compartment, recruitment of the ATG12-5-16L1 complex, and LC3 lipidation (Lamb et al., 2013). More recently, it has become clear that the transcriptional activation of autophagy and lysosome genes plays an important role in prolonged autophagy (Füllgrabe et al., 2014). Although the link between nutrient sensing by mTORC1 and TFEB-mediated autophagy/lysosome gene activation is now well recognized, other signaling pathways that mediate the nutrient regulation of autophagy genes are not fully understood. In this regard, we report that a signaling cascade consisting of two nutrient sensor molecules—AMPK and SIRT1—integrate the nutrient status of the cell to autophagy gene regulation via BRD4. Nutrient deprivation causes the dissociation of SIRT1 from its inhibitory molecule DBC1 in an AMPK-dependent manner and may also activate SIRT1 via an increase in NAD^+/NADH ratio (Cantó et al., 2009), which in turn leads to SIRT1-mediated BRD4 dissociation from autophagy gene promoters and de-repression of autophagy gene expression. Our findings suggest that BRD4 suppresses autophagy and lysosome gene expression under nutrient-replete conditions to prevent excess autophagy, and BRD4 dissociation allows cells to maintain autophagic activity during prolonged nutrient shortage. Interestingly, a recent report showing that JQ1 increases LC3 lipidation and autophagosome formation implicates the involvement of BET proteins in autophagy regulation (Jang et al., 2016). Importantly, however, the detailed mechanism by which JQ1 modulates autophagy and the molecule that mediates this effect were not explored.

Currently, TFEB is thought to be a "master" regulator of autophagy and lysosome gene transcription (Settembre et al., 2011). Importantly, BRD4 inhibition activates autophagy in the absence of TFEB and its related molecules TFE3 and MITF. This observation suggests that BRD4 orchestrates a distinct transcriptional program and is therefore another crucial regulator of autophagy and lysosome gene expression. Interestingly, a recent report has shown that AMPK activates TFEB-mediated transcription by inducing the transcriptional coactivator, Coactivator associated arginine (R) methyltransferase 1 (CARM1) (Shin et al., 2016). These findings, taken together, suggest that AMPK activation upon nutrient deprivation stimulates TFEB-mediated transcription and suppresses BRD4 function to cooperatively activate the autophagy-lysosome pathway.

BRD4 has been proposed as a positive regulator of transcription that bridges histone acetylation and transcriptional regulators such as P-TEFb and the Mediator complex (Shi and Vakoc, 2014). We observed, as previously reported, that this positive effect on transcription is particularly important for the regulation of genes involved in the promotion of cell growth (Zuber et al., 2011), and so it is interesting that BRD4 at the same time reciprocally represses genes involved in major catabolic processes at the lysosome and vice versa. Interestingly, although this is not the first report to show that BRD4 functions as a transcriptional

Figure 5. BRD4 Represses Autophagy Gene Expression through G9a

(A) Cell extracts from KP-4 cells were subjected to immunoprecipitation with G9a (upper) and BRD4 (lower) antibodies.

(B) KP-4 cells were starved for 4 hr followed by immunoprecipitation with BRD4 antibody.

(C–F) KP-4 cells were treated with 500 nM JQ1 for 9 hr (C and E). KP-4 cells harboring inducible control or BRD4 shRNA were treated with 500 ng/mL doxycycline (DOX) for 4 days (D and F). ChIP assays were performed using G9a (C and D) and H3K9diMe (E and F) antibodies.

(G and H) KP-4 cells infected with shRNA targeting G9a were transfected with BRD4 siRNA followed by RT-qPCR (G) and western blot (H).

(I) KP-4 cells overexpressing BRD4 were infected with shRNA targeting G9a.

All data are shown as mean ± SD. In (C)–(G), n = 3 independent experiments. *$p < 0.01$, **$p < 0.05$, N.S., no significance. See also Figure S5.

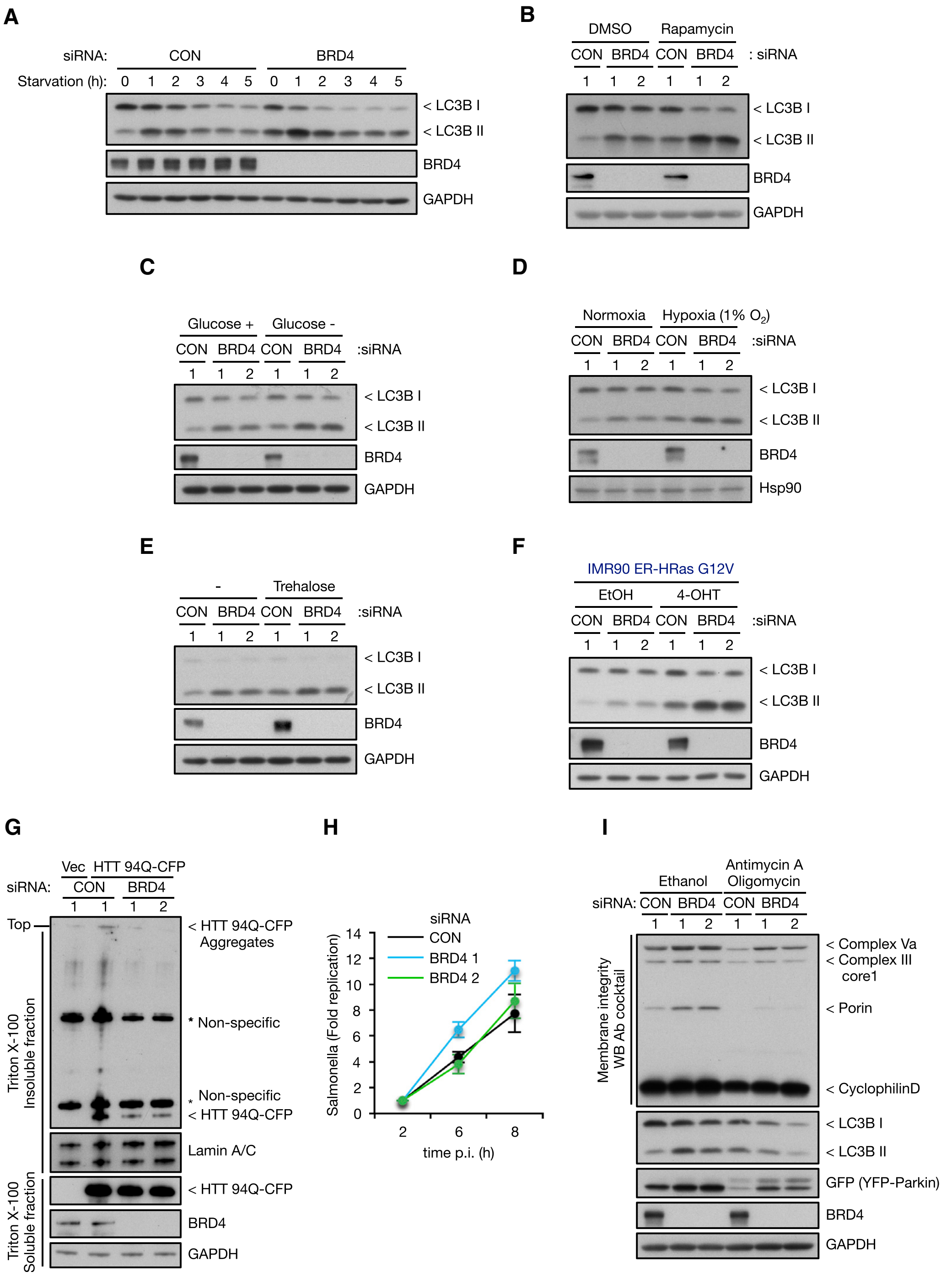

Figure 6. Effect of BRD4 Silencing on Stimulus-Dependent and Selective Autophagy

(A–F) Cells transfected with BRD4 siRNA were starved for 1–5 hr (KP-4 cells, A), treated with 500 nM rapamycin for 24 hr (KP-4 cells, B), starved of glucose for 4 hr (KP-4 cells, C), cultured under hypoxic (1% O_2) conditions for 48 hr (SUIT2 cells, D), treated with 100 mM Trehalose for 4 hr (KP-4 cells, E), or treated with 500 nM 4-Hydroxytamoxifen (4-OHT) for 48 hr (IMR90 ER-HRas G12V cells, F).

(G) KP-4 cells harboring rtTA and Tre-tight-HTT Q94-CFP were transfected with BRD4 siRNA. At 12 hr after transfection, cells were treated with 1 μg/mL DOX for 10 hr. At 48 hr after removal of DOX, cells were separated into Triton X-100 soluble and insoluble fractions.

(H) KP-4 cells transfected with BRD4 siRNA were infected with *Salmonella enterica* serovar Typhimurium. The number of *Salmonella* was determined by performing colony-forming unit assays at 2, 6, and 8 hr after infection and normalized to the numbers at 2 hr. Data are shown as mean ± SEM; n = 4 independent experiments.

(I) KP-4 cells expressing YFP-parkin were transfected with BRD4 siRNA followed by treatment with 1 μM Antimycin A and 1 μM Oligomycin for 8 hr.

See also Figure S6.

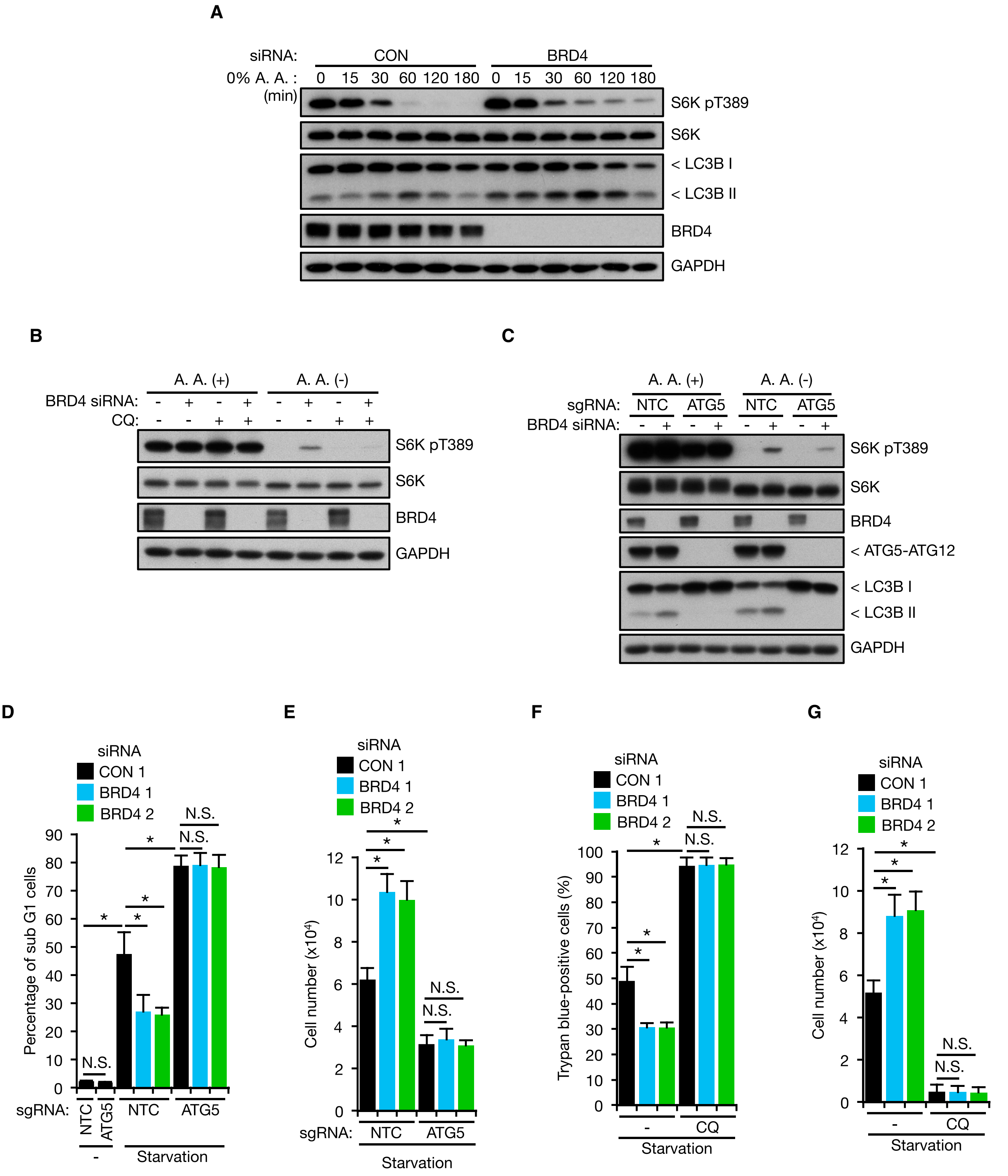

Figure 7. BRD4 Knockdown Sustains mTOR Activity during Starvation and Confers Resistance to Starvation-Induced Cell Death

(A and B) KP-4 cells transfected with BRD4 siRNA were starved of amino acids (A). Cells pre-treated with CQ (10 μM, 4 hr) were subjected to amino acid starvation for 2 hr in the presence of CQ (B).

(C) KP-4 cells infected with Cas9/ATG5 sgRNA were transfected with BRD4 siRNA and subjected to amino acid starvation for 2 hr.

(legend continued on next page)

repressor, the mechanisms behind this effect were largely unknown (Barrow et al., 2016). We show that BRD4 is recruited to autophagy and lysosome gene promoter regions and interacts with G9a that deposits a repressive H3K9diMe mark. However, our finding that G9a knockdown largely, but not completely, abrogates autophagy repression by BRD4 overexpression implies that there are other mechanism(s) that mediate the autophagy suppression by BRD4. Different transcriptional activators (i.e., FOXO family and p53 family), repressors (i.e., ZKSCAN3 and FOXK), and histone modifications (i.e., H3K27triMe and H2BK120Ub) are also involved in the transcriptional regulation of autophagy (Baek and Kim, 2017; Füllgrabe et al., 2016). Therefore, it remains possible that BRD4 affects the recruitment of these transcription factors and histone-modifying enzymes to suppress autophagy gene expression.

Modulation of autophagic activity is thought to be a potential therapeutic strategy for various diseases, including neuronal degeneration, infectious diseases, and cancer (Rubinsztein et al., 2012). In this regard, identification of druggable autophagy regulators would be an attractive strategy to treat these diseases. BET inhibitors exhibit anti-tumor effects in various types of cancers and have been tested in phase one and two clinical trials (Wang and Filippakopoulos, 2015). Our findings also potentially indicate that BET inhibitors may have beneficial effects in diseases, such as neurodegeneration, where promotion of autophagy is being explored for therapy.

There has been much interest in whether autophagy represses or promotes tumor development (Galluzzi et al., 2015) and so the fact that the product of a chromosomal translocation considered to be the driver of NMC is a repressor of autophagy is an interesting discovery. Interestingly, we found that, in NMC cells, BRD4 expressed from the unaffected allele has little contribution to the regulation of autophagy, indicating a dominant role of BRD4-NUT in autophagy repression. How this enhanced repression is achieved and how much, if at all, the repression of autophagy is a contributing factor in the development of NMC are beyond the scope of this current study but undoubtedly worthy of future investigation.

Taken together, the findings we present here detail a mechanism of transcriptional regulation of autophagy and lysosome function. The mechanism facilitates some forms of autophagy, but not others, and this therefore highlights an additional control point of autophagy regulation that may be relevant to various forms of human disease.

STAR★METHODS

Detailed methods are provided in the online version of this paper and include the following:

- KEY RESOURCES TABLE
- CONTACT FOR REAGENT AND RESOURCE SHARING
- EXPERIMENTAL MODEL AND SUBJECT DETAILS
  - Cell Culture
  - Mice
- METHOD DETAILS
  - Reagents
  - Plasmid Transfection
  - siRNA Transfection
  - Western Blotting
  - Plasmids, sgRNAs, and shRNAs
  - Lentivirus and Retrovirus Production and Infection
  - Immunofluorescence and Confocal Imaging
  - Immunohistochemistry
  - RNA Sequencing
  - RNA Isolation, Reverse Transcription, and Quantitative PCR (RT-qPCR)
  - Co-immunoprecipitation
  - Chromatin Immunoprecipitation
  - RNAi in *Drosophila* S2R+ cells
  - β-Hexosaminidase Assay
  - Aggrephagy
  - Mitophagy
  - Xenophagy
  - Cell Proliferation and Cell Death Assays
- QUANTIFICATION AND STATISTICAL ANALYSIS
  - Quantification of Western Blotting
  - Quantification of Microscopic Images
  - Quantification of the qPCR Results
  - Statistical Analyses
- DATA AND SOFTWARE AVAILABILITY

SUPPLEMENTAL INFORMATION

Supplemental Information includes seven figures and two tables and can be found with this article online at http://dx.doi.org/10.1016/j.molcel.2017.04.027.

AUTHOR CONTRIBUTIONS

J.S. designed the project, conducted most of the experiments, analyzed data, and wrote the manuscript. S.W. conducted the RNAi screening in *Drosophila* S2R+ cells. M.H. conducted xenophagy experiments under the supervision of I.D. N.T. generated BRD4 knockdown mice, performed JQ1 treatment in mice, and prepared tissue sections under the supervision of S.W.L. J.O. generated pBabe-mRFP-GFP-LC3 and lentiCRISPR-NTC, ATG5 plasmids, and KP-4 cells expressing Cas9/ATG5 sgRNA and helped with experiments. W.C. performed RNA-seq. A.H. conducted analysis of the RNA-seq data. C.N. performed immunohistochemistry staining. J.S.L. generated KP-4 cells expressing YFP-parkin and helped with experiments. M.N. and T.V.A. conducted the initial evaluation of autophagic flux in cell lines used in this study and provided advice regarding the autophagy experiments under the supervision of S.A.T. K.M.R. conceived and supervised the project and wrote the manuscript.

ACKNOWLEDGMENTS

We thank members of the Ryan Laboratory for helpful discussions. We also thank Direna Alonso Curbelo for advice on BRD4 IHC staining and Peter

(D and E) KP-4 cells infected with Cas9/ATG5 sgRNA were transfected with BRD4 siRNA. Following 48 hr starvation, percentage of subG1 cells (D) and cell number (E) were determined (n = 3 independent experiments).
(F and G) KP-4 cells transfected with BRD4 siRNA were starved for 48 hr in the presence or absence of 10 μM CQ. Percentage of dead (F) and surviving (G) cells was determined by trypan blue exclusion test (n = 4 independent experiments).
All data are shown as mean ± SD. *p < 0.01, N.S., no significance. See also Figure S7.

Adams and David Rubinsztein for advice. Work in the Ryan laboratory is supported by Cancer Research UK (C596/A17196), Astellas Pharma, and Worldwide Cancer Research (16-1194). S.W.L. is a founder and consultant for Mirimus, a company that has commercialized the inducible shRNA transgenic technology used in this study.

Received: November 28, 2016
Revised: March 15, 2017
Accepted: April 28, 2017
Published: May 18, 2017

REFERENCES

Alexeyev, M.F., Fayzulin, R., Shokolenko, I.N., and Pastukh, V. (2010). A retro-lentiviral system for doxycycline-inducible gene expression and gene knockdown in cells with limited proliferative capacity. Mol. Biol. Rep. *37*, 1987–1991.

Baek, S.H., and Kim, K.I. (2017). Epigenetic control of autophagy: nuclear events gain more attention. Mol. Cell *65*, 781–785.

Barrow, J.J., Balsa, E., Verdeguer, F., Tavares, C.D., Soustek, M.S., Hollingsworth, L.R., 4th, Jedrychowski, M., Vogel, R., Paulo, J.A., Smeitink, J., et al. (2016). Bromodomain inhibitors correct bioenergetic deficiency caused by mitochondrial disease complex I mutations. Mol. Cell *64*, 163–175.

Baudot, A.D., Haller, M., Mrschtik, M., Tait, S.W., and Ryan, K.M. (2015). Using enhanced-mitophagy to measure autophagic flux. Methods *75*, 105–111.

Bauer, P.O., Goswami, A., Wong, H.K., Okuno, M., Kurosawa, M., Yamada, M., Miyazaki, H., Matsumoto, G., Kino, Y., Nagai, Y., and Nukina, N. (2010). Harnessing chaperone-mediated autophagy for the selective degradation of mutant huntingtin protein. Nat. Biotechnol. *28*, 256–263.

Bisgrove, D.A., Mahmoudi, T., Henklein, P., and Verdin, E. (2007). Conserved P-TEFb-interacting domain of BRD4 inhibits HIV transcription. Proc. Natl. Acad. Sci. USA *104*, 13690–13695.

Bolden, J.E., Tasdemir, N., Dow, L.E., van Es, J.H., Wilkinson, J.E., Zhao, Z., Clevers, H., and Lowe, S.W. (2014). Inducible in vivo silencing of Brd4 identifies potential toxicities of sustained BET protein inhibition. Cell Rep. *8*, 1919–1929.

Cantó, C., Gerhart-Hines, Z., Feige, J.N., Lagouge, M., Noriega, L., Milne, J.C., Elliott, P.J., Puigserver, P., and Auwerx, J. (2009). AMPK regulates energy expenditure by modulating NAD+ metabolism and SIRT1 activity. Nature *458*, 1056–1060.

Chang, C., Su, H., Zhang, D., Wang, Y., Shen, Q., Liu, B., Huang, R., Zhou, T., Peng, C., Wong, C.C., et al. (2015). AMPK-dependent phosphorylation of GAPDH triggers Sirt1 activation and is necessary for autophagy upon glucose starvation. Mol. Cell *60*, 930–940.

Chauhan, S., Goodwin, J.G., Chauhan, S., Manyam, G., Wang, J., Kamat, A.M., and Boyd, D.D. (2013). ZKSCAN3 is a master transcriptional repressor of autophagy. Mol. Cell *50*, 16–28.

Church, D.M., Schneider, V.A., Graves, T., Auger, K., Cunningham, F., Bouk, N., Chen, H.C., Agarwala, R., McLaren, W.M., Ritchie, G.R., et al. (2011). Modernizing reference genome assemblies. PLoS Biol. *9*, e1001091.

Dawson, M.A., Prinjha, R.K., Dittmann, A., Giotopoulos, G., Bantscheff, M., Chan, W.I., Robson, S.C., Chung, C.W., Hopf, C., Savitski, M.M., et al. (2011). Inhibition of BET recruitment to chromatin as an effective treatment for MLL-fusion leukaemia. Nature *478*, 529–533.

Filippakopoulos, P., Qi, J., Picaud, S., Shen, Y., Smith, W.B., Fedorov, O., Morse, E.M., Keates, T., Hickman, T.T., Felletar, I., et al. (2010). Selective inhibition of BET bromodomains. Nature *468*, 1067–1073.

Fisher, S., Barry, A., Abreu, J., Minie, B., Nolan, J., Delorey, T.M., Young, G., Fennell, T.J., Allen, A., Ambrogio, L., et al. (2011). A scalable, fully automated process for construction of sequence-ready human exome targeted capture libraries. Genome Biol. *12*, R1.

Floyd, S.R., Pacold, M.E., Huang, Q., Clarke, S.M., Lam, F.C., Cannell, I.G., Bryson, B.D., Rameseder, J., Lee, M.J., Blake, E.J., et al. (2013). The bromodomain protein Brd4 insulates chromatin from DNA damage signalling. Nature *498*, 246–250.

French, C.A. (2010). NUT midline carcinoma. Cancer Genet. Cytogenet. *203*, 16–20.

Füllgrabe, J., Lynch-Day, M.A., Heldring, N., Li, W., Struijk, R.B., Ma, Q., Hermanson, O., Rosenfeld, M.G., Klionsky, D.J., and Joseph, B. (2013). The histone H4 lysine 16 acetyltransferase hMOF regulates the outcome of autophagy. Nature *500*, 468–471.

Füllgrabe, J., Klionsky, D.J., and Joseph, B. (2014). The return of the nucleus: transcriptional and epigenetic control of autophagy. Nat. Rev. Mol. Cell Biol. *15*, 65–74.

Füllgrabe, J., Ghislat, G., Cho, D.H., and Rubinsztein, D.C. (2016). Transcriptional regulation of mammalian autophagy at a glance. J. Cell Sci. *129*, 3059–3066.

Galluzzi, L., Pietrocola, F., Bravo-San Pedro, J.M., Amaravadi, R.K., Baehrecke, E.H., Cecconi, F., Codogno, P., Debnath, J., Gewirtz, D.A., Karantza, V., et al. (2015). Autophagy in malignant transformation and cancer progression. EMBO J. *34*, 856–880.

Guan, B., Wang, T.L., and Shih, IeM. (2011). ARID1A, a factor that promotes formation of SWI/SNF-mediated chromatin remodeling, is a tumor suppressor in gynecologic cancers. Cancer Res. *71*, 6718–6727.

Hale, C.M., Cheng, Q., Ortuno, D., Huang, M., Nojima, D., Kassner, P.D., Wang, S., Ollmann, M.M., and Carlisle, H.J. (2016). Identification of modulators of autophagic flux in an image-based high content siRNA screen. Autophagy *12*, 713–726.

Itakura, E., and Mizushima, N. (2011). p62 Targeting to the autophagosome formation site requires self-oligomerization but not LC3 binding. J. Cell Biol. *192*, 17–27.

Jang, J.E., Eom, J.I., Jeung, H.K., Cheong, J.W., Lee, J.Y., Kim, J.S., and Min, Y.H. (2016). AMPK-ULK1-mediated autophagy confers resistance to BET inhibitor JQ1 in acute myeloid leukemia stem cells. Clin. Cancer Res. Published online November 18, 2016. http://dx.doi.org/10.1158/1078-0432.CCR-16-1903.

Kellner, W.A., Van Bortle, K., Li, L., Ramos, E., Takenaka, N., and Corces, V.G. (2013). Distinct isoforms of the Drosophila Brd4 homologue are present at enhancers, promoters and insulator sites. Nucleic Acids Res. *41*, 9274–9283.

Khaminets, A., Behl, C., and Dikic, I. (2016). Ubiquitin-dependent and independent signals in selective autophagy. Trends Cell Biol. *26*, 6–16.

Kim, D., Pertea, G., Trapnell, C., Pimentel, H., Kelley, R., and Salzberg, S.L. (2013). TopHat2: accurate alignment of transcriptomes in the presence of insertions, deletions and gene fusions. Genome Biol. *14*, R36.

Kimura, S., Noda, T., and Yoshimori, T. (2007). Dissection of the autophagosome maturation process by a novel reporter protein, tandem fluorescent-tagged LC3. Autophagy *3*, 452–460.

Klionsky, D.J., Abdelmohsen, K., Abe, A., Abedin, M.J., Abeliovich, H., Acevedo Arozena, A., Adachi, H., Adams, C.M., Adams, P.D., Adeli, K., et al. (2016). Guidelines for the use and interpretation of assays for monitoring autophagy (3rd edition). Autophagy *12*, 1–222.

Korb, E., Herre, M., Zucker-Scharff, I., Darnell, R.B., and Allis, C.D. (2015). BET protein Brd4 activates transcription in neurons and BET inhibitor Jq1 blocks memory in mice. Nat. Neurosci. *18*, 1464–1473.

Ktistakis, N.T., and Tooze, S.A. (2016). Digesting the expanding mechanisms of autophagy. Trends Cell Biol. *26*, 624–635.

Lamb, C.A., Yoshimori, T., and Tooze, S.A. (2013). The autophagosome: origins unknown, biogenesis complex. Nat. Rev. Mol. Cell Biol. *14*, 759–774.

Langmead, B., and Salzberg, S.L. (2012). Fast gapped-read alignment with Bowtie 2. Nat. Methods *9*, 357–359.

Love, M.I., Huber, W., and Anders, S. (2014). Moderated estimation of fold change and dispersion for RNA-seq data with DESeq2. Genome Biol. *15*, 550.

Lu, J., Qian, Y., Altieri, M., Dong, H., Wang, J., Raina, K., Hines, J., Winkler, J.D., Crew, A.P., Coleman, K., and Crews, C.M. (2015). Hijacking the E3 ubiquitin ligase cereblon to efficiently target BRD4. Chem. Biol. *22*, 755–763.

Maynard, C.J., Böttcher, C., Ortega, Z., Smith, R., Florea, B.I., Díaz-Hernández, M., Brundin, P., Overkleeft, H.S., Li, J.Y., Lucas, J.J., and Dantuma, N.P. (2009). Accumulation of ubiquitin conjugates in a polyglutamine

disease model occurs without global ubiquitin/proteasome system impairment. Proc. Natl. Acad. Sci. USA *106*, 13986–13991.

McEwan, D.G., Popovic, D., Gubas, A., Terawaki, S., Suzuki, H., Stadel, D., Coxon, F.P., Miranda de Stegmann, D., Bhogaraju, S., Maddi, K., et al. (2015a). PLEKHM1 regulates autophagosome-lysosome fusion through HOPS complex and LC3/GABARAP proteins. Mol. Cell *57*, 39–54.

McEwan, D.G., Richter, B., Claudi, B., Wigge, C., Wild, P., Farhan, H., McGourty, K., Coxon, F.P., Franz-Wachtel, M., Perdu, B., et al. (2015b). PLEKHM1 regulates Salmonella-containing vacuole biogenesis and infection. Cell Host Microbe *17*, 58–71.

Mizushima, N., Levine, B., Cuervo, A.M., and Klionsky, D.J. (2008). Autophagy fights disease through cellular self-digestion. Nature *451*, 1069–1075.

Morgenstern, J.P., and Land, H. (1990). Advanced mammalian gene transfer: high titre retroviral vectors with multiple drug selection markers and a complementary helper-free packaging cell line. Nucleic Acids Res. *18*, 3587–3596.

Narain, Y., Wyttenbach, A., Rankin, J., Furlong, R.A., and Rubinsztein, D.C. (1999). A molecular investigation of true dominance in Huntington's disease. J. Med. Genet. *36*, 739–746.

Perera, R.M., Stoykova, S., Nicolay, B.N., Ross, K.N., Fitamant, J., Boukhali, M., Lengrand, J., Deshpande, V., Selig, M.K., Ferrone, C.R., et al. (2015). Transcriptional control of autophagy-lysosome function drives pancreatic cancer metabolism. Nature *524*, 361–365.

Premsrirut, P.K., Dow, L.E., Kim, S.Y., Camiolo, M., Malone, C.D., Miething, C., Scuoppo, C., Zuber, J., Dickins, R.A., Kogan, S.C., et al. (2011). A rapid and scalable system for studying gene function in mice using conditional RNA interference. Cell *145*, 145–158.

Rabinowitz, J.D., and White, E. (2010). Autophagy and metabolism. Science *330*, 1344–1348.

Reimand, J., Arak, T., and Vilo, J. (2011). g:Profiler—a web server for functional interpretation of gene lists (2011 update). Nucleic Acids Res. *39*, W307–W315.

Roczniak-Ferguson, A., Petit, C.S., Froehlich, F., Qian, S., Ky, J., Angarola, B., Walther, T.C., and Ferguson, S.M. (2012). The transcription factor TFEB links mTORC1 signaling to transcriptional control of lysosome homeostasis. Sci. Signal. *5*, ra42.

Rubinsztein, D.C., Codogno, P., and Levine, B. (2012). Autophagy modulation as a potential therapeutic target for diverse diseases. Nat. Rev. Drug Discov. *11*, 709–730.

Sanjana, N.E., Shalem, O., and Zhang, F. (2014). Improved vectors and genome-wide libraries for CRISPR screening. Nat. Methods *11*, 783–784.

Schwartz, B.E., Hofer, M.D., Lemieux, M.E., Bauer, D.E., Cameron, M.J., West, N.H., Agoston, E.S., Reynoird, N., Khochbin, S., Ince, T.A., et al. (2011). Differentiation of NUT midline carcinoma by epigenomic reprogramming. Cancer Res. *71*, 2686–2696.

Settembre, C., Di Malta, C., Polito, V.A., Garcia Arencibia, M., Vetrini, F., Erdin, S., Erdin, S.U., Huynh, T., Medina, D., Colella, P., et al. (2011). TFEB links autophagy to lysosomal biogenesis. Science *332*, 1429–1433.

Shen, H.M., and Mizushima, N. (2014). At the end of the autophagic road: an emerging understanding of lysosomal functions in autophagy. Trends Biochem. Sci. *39*, 61–71.

Shi, J., and Vakoc, C.R. (2014). The mechanisms behind the therapeutic activity of BET bromodomain inhibition. Mol. Cell *54*, 728–736.

Shin, H.J., Kim, H., Oh, S., Lee, J.G., Kee, M., Ko, H.J., Kweon, M.N., Won, K.J., and Baek, S.H. (2016). AMPK-SKP2-CARM1 signalling cascade in transcriptional regulation of autophagy. Nature *534*, 553–557.

Shinkai, Y., and Tachibana, M. (2011). H3K9 methyltransferase G9a and the related molecule GLP. Genes Dev. *25*, 781–788.

Stadel, D., Millarte, V., Tillmann, K.D., Huber, J., Tamin-Yecheskel, B.C., Akutsu, M., Demishtein, A., Ben-Zeev, B., Anikster, Y., Perez, F., et al. (2015). TECPR2 cooperates with LC3C to regulate COPII-dependent ER export. Mol. Cell *60*, 89–104.

Vaquero, A., Sternglanz, R., and Reinberg, D. (2007). NAD+-dependent deacetylation of H4 lysine 16 by class III HDACs. Oncogene *26*, 5505–5520.

Wang, C.Y., and Filippakopoulos, P. (2015). Beating the odds: BETs in disease. Trends Biochem. Sci. *40*, 468–479.

Wang, T., Wei, J.J., Sabatini, D.M., and Lander, E.S. (2014). Genetic screens in human cells using the CRISPR-Cas9 system. Science *343*, 80–84.

Wilkinson, S., Croft, D.R., O'Prey, J., Meedendorp, A., O'Prey, M., Dufès, C., and Ryan, K.M. (2011). The cyclin-dependent kinase PITSLRE/CDK11 is required for successful autophagy. Autophagy *7*, 1295–1301.

Williams, A., Sarkar, S., Cuddon, P., Ttofi, E.K., Saiki, S., Siddiqi, F.H., Jahreiss, L., Fleming, A., Pask, D., Goldsmith, P., et al. (2008). Novel targets for Huntington's disease in an mTOR-independent autophagy pathway. Nat. Chem. Biol. *4*, 295–305.

Zippo, A., Serafini, R., Rocchigiani, M., Pennacchini, S., Krepelova, A., and Oliviero, S. (2009). Histone crosstalk between H3S10ph and H4K16ac generates a histone code that mediates transcription elongation. Cell *138*, 1122–1136.

Zuber, J., Shi, J., Wang, E., Rappaport, A.R., Herrmann, H., Sison, E.A., Magoon, D., Qi, J., Blatt, K., Wunderlich, M., et al. (2011). RNAi screen identifies Brd4 as a therapeutic target in acute myeloid leukaemia. Nature *478*, 524–528.

STAR★METHODS

KEY RESOURCES TABLE

REAGENT or RESOURCE	SOURCE	IDENTIFIER
Antibodies		
Rabbit monoclonal anti-BRD4 (clone E2A7X) (human specific) (long isoform)	Cell Signaling Technology	Cat#: 13440S
Rabbit monoclonal anti-NUT (clone C52B1)	Cell Signaling Technology	Cat#: 3625S; RRID: AB_2066833
Rabbit monoclonal anti-G9a (EHMT2/KMT1C) (clone C6H3)	Cell Signaling Technology	Cat#: 3306S; RRID: AB_2097647
Rabbit monoclonal anti-SIRT1 (clone D1D7)	Cell Signaling Technology	Cat#: 9475P; RRID: AB_2617130
Mouse monoclonal anti-DBC1 (clone 3G4)	Cell Signaling Technology	Cat#: 5857S; RRID: AB_10838138
Rabbit monoclonal anti-AMPKα pT172	Cell Signaling Technology	Cat#: 2535S; RRID: AB_331250
Mouse monoclonal anti-AMPKα1/α2	Cell Signaling Technology	Cat#: 2793S; RRID: AB_915794
Rabbit polyclonal anti-LC3B	Cell Signaling Technology	Cat#: 2775S; RRID: AB_915950
Rabbit monoclonal anti-LC3B (clone D11)	Cell Signaling Technology	Cat#: 3868S; RRID: AB_2137707
Rabbit polyclonal anti-TFEB	Cell Signaling Technology	Cat#: 4240S; RRID: AB_11220225
Rabbit monoclonal anti-LAMP1 (clone D2D11)	Cell Signaling Technology	Cat#: 9091P
Rabbit polyclonal anti-Cathepsin D	Cell Signaling Technology	Cat#: 2284S; RRID: AB_10694258
Rabbit polyclonal anti-ASM	Cell Signaling Technology	Cat# 3687S; RRID: AB_1904135
Rabbit monoclonal anti-Cathepsin B (clone D1C7Y)	Cell Signaling Technology	Cat#: 31718S
Rabbit monoclonal anti-ATG5 (clone D5F5U)	Cell Signaling Technology	Cat#: 12994S
Rabbit monoclonal anti-p70 S6K pT389 (clone 108D2)	Cell Signaling Technology	Cat#: 9234S; RRID: AB_2269803
Rabbit monoclonal anti-p70 S6K (clone 49D7)	Cell Signaling Technology	Cat#: 2708S; RRID: AB_390722
Rabbit monoclonal anti-Acetyl-Histone H4 (Lys16) (clone E2B8W)	Cell Signaling Technology	Cat#:13534S
Mouse monoclonal anti-Histone H4 (clone L64C1)	Cell Signaling Technology	Cat#: 2935P; RRID: AB_1147658
Rabbit monoclonal anti-c-Myc (clone D84C12)	Cell Signaling Technology	Cat#: 5605S; RRID: AB_1903938
Rabbit monoclonal anti-CDK9 (clone C12F7)	Cell Signaling Technology	Cat#: 2316T; RRID: AB_2291505
Rabbit monoclonal anti-GFP (clone D5.1)	Cell Signaling Technology	Cat#: 2956S; RRID: AB_1196615
Rabbit polyclonal anti-TFE3	Cell Signaling Technology	Cat#: 14779S
Normal rabbit IgG	Cell Signaling Technology	Cat#: 2729S; RRID: AB_1031062
Mouse IgG1 isotype control G3A1	Cell Signaling Technology	Cat#: 5415S; RRID: AB_10829607
Rabbit monoclonal anti-BRD4 (clone EPR5150(2)) (human and mouse) (long and short isoforms)	Abcam	Cat#: ab128874; RRID: AB_11145462
Mouse monoclonal anti-GAPDH	Abcam	Cat#: ab9484; RRID: AB_307274
Rabbit polyclonal anti-β-actin	Abcam	Cat#: ab8227; RRID: AB_2305186
Rabbit polyclonal anti-G9a (EHMT2/KMT1C)	Abcam	Cat#: ab133482
Mouse monoclonal anti-Histone H3 (di methyl K9)	Abcam	Cat#: ab1220; RRID: AB_449854
Rabbit polyclonal anti-GAA (clone EPR4716(2))	Abcam	Cat#: ab137068
Mouse monoclonal Membrane Integrity WB Antibody Cocktail	Abcam	Cat#: ab110414
Rabbit polyclonal anti-BRD4 (human and mouse) (long isoform)	Bethyl Laboratories	Cat#: A301-985A50; RRID: AB_2631449
Rabbit polyclonal anti-BRD2	Bethyl Laboratories	Cat#: A302-583A; RRID: AB_2034829
Rabbit polyclonal anti-BRD3	Bethyl Laboratories	Cat#: A302-368A; RRID: AB_1907251
Rabbit polyclonal anti-hMOF (MYST1/KAT8)	Bethyl Laboratories	Cat#: A300-992A; RRID: AB_805802
Rabbit polyclonal anti-Acetyl-Histone H4 (Lys16)	Millipore	Cat#: 07-329; RRID: AB_310525
Rabbit polyclonal anti-Acetyl-Histone H4 (Lys16)	Active Motif	Cat#: 39167

(*Continued on next page*)

Continued

REAGENT or RESOURCE	SOURCE	IDENTIFIER
Mouse monoclonal anti-LAMP2/CD107b	BD Biosciences	Cat#: 555803; RRID: AB_396137
Mouse monoclonal anti-p62	BD Biosciences	Cat#: 610833; RRID: AB_398152
Mouse monoclonal anti-GFP	Covance	Cat#: MMS-118P-200; RRID: AB_10063778
Goat polyclonal anti-LaminA/C	Santa Cruz	Cat#: sc-6215; RRID: AB_648152
Goat polyclonal anti-HSP90β	Santa Cruz	Cat#: sc-1057; RRID: AB_2121392
Mouse monoclonal anti-FLAG (clone M2)	Sigma-Aldrich	Cat#: F1804; RRID: AB_262044
Mouse monoclonal anti-V5	Invitrogen	Cat#: 46-0705
Mouse monoclonal anti-WIPI2	Bio-Rad	Cat#: MCA5780GA; RRID: AB_10845951
Mouse monoclonal anti-LC3	NanoTools	Cat#: 0231-100/LC3-5F10
**Bacterial and Virus Strains**		
Salmonella enterica serovar Typhimurium (strain 12023)	David Holden Lab (Imperial College London)	N/A
**Chemicals, Peptides, and Recombinant Proteins**		
Chloroquine	Sigma-Aldrich	Cat#: C6628
Doxycycline	Sigma-Aldrich	Cat#: D9891
Antimycin A	Sigma-Aldrich	Cat#: A8674
Oligomycin	Sigma-Aldrich	Cat#: O4876
D-(+)-Trehalose dihydrate	Sigma-Aldrich	Cat#: T0167
4-Hydroxytamoxifen	Sigma-Aldrich	Cat#: H7904
(+)-JQ1	TOCRIS	Cat#: 4499
I-BET151	TOCRIS	Cat#: 4650
OTX015	Cayman	Cat#: 15947
Compound C	EMD Millipore	Cat#: 171264
Rapamycin	LC Laboratories	Cat#: R-5000
ARV-825	Chemietek	Cat#: CT-ARV825
LysoTracker red DND-99	Thermo Fisher Scientific	Cat# L7528
Magic Red Cathepsin B	ImmunoChemistry Technologies	Cat#: 938
Hoechst33342	Thermo Fisher Scientific	Cat#: H3570
**Critical Commercial Assays**		
TruSeq RNA Sample Prep Kit v2	Illumina	Cat#: RS-122-2001
Click-iT EdU Flow Cytometry Assay Kit	Thermo Fisher Scientific	Cat#: C10633
**Deposited Data**		
Raw and processed data of the RNA-seq	This paper	GEO: GSE90444
Full scans of western blot data and original microscopy images	This paper	Mendeley Data: http://dx.doi.org/10.17632/ksz4pmwkdb.1
**Experimental Models: Cell Lines**		
Human: KP-4	RIKEN	RCB1005
Human: PA-TU-8902	DMSZ	ACC-179
Human: SUIT2	JCRB	JCRB1094
Human: PA-TU-8988T	DMSZ	ACC-162
Human: hTERT-HPNE	ATCC	CRL-4023
Human: IMR90 ER-HRas G12V	Peter D. Adams Lab (Cancer Research UK Beatson Institute)	N/A
Human: HEK293T	ATCC	CRL-3216
Human: PK-1	RIKEN	RCB1972
Human: TY-82	JCRB	JCRB1330
Human: Phoenix-AMPHO	ATCC	CRL-3213

(*Continued on next page*)

Continued

REAGENT or RESOURCE	SOURCE	IDENTIFIER
Experimental Models: Organisms/Strains		
Mouse: CAGs-rtTA3	Scott W. Lowe Lab (Memorial Sloan Kettering Cancer Center)	Premsrirut et al., 2011 (also available from the Jackson Laboratory, Stock #: 016532)
Mouse: TRE-shRen	Scott W. Lowe Lab	N/A
Mouse: TRE-shBRD4	Scott W. Lowe Lab	N/A
Mouse: C57BL/6J	The Jackson Laboratory	Stock #: 000664
Oligonucleotides		
siRNAs, see the Table S1	N/A	N/A
pPCR primers, See the Table S2	N/A	N/A
Recombinant DNA		
pBabe-puro	Morgenstern and Land, 1990	Addgene plasmid # 1764
pRetrox-tight-puro-HA-BRD4	MRC Protein Phosphorylation and Ubiquitylation Unit	Cat#: DU46347
pBabe-puro-human BRD4 long variant	This paper	N/A
pLenti-puro	Guan et al., 2011	Addgene plasmid # 39481
pLenti-puro-human BRD4 long variant	This paper	N/A
pEGFP-C1+mRFP-LC3	Tamotsu Yoshimori Lab (Osaka University)	Kimura et al., 2007
pBabe-puro-mRFP-GFP-LC3	This paper	N/A
pLZRS-YFP-Parkin	Baudot et al., 2015	N/A
pcDNA3	Invitrogen	N/A
pcDNA3-human BRD4 short variant	This paper	N/A
pLenti6-MK1-EHMT2 (G9a)-V5 (human G9a long variant)	Addgene	Addgene plasmid # 31113
pTRE-tight	Clontech	Cat#: 631059
pTRE-tight-Htt94Q-CFP	Maynard et al., 2009	Addgene plasmid #23966
pMA2640	Alexeyev et al., 2010	Addgene plasmid #25434
pFlag-CMV2-Brd4 (1-1362) (human BRD4 long variant)	Bisgrove et al., 2007	Addgene plasmid #22304, discontinued due to reason other than plasmid issue
psPAX2	Addgene	Addgene plasmid #12260
pMD2.G	Addgene	Addgene plasmid #12259
pMXs-puro GFP-p62	Itakura and Mizushima, 2011	Addgene plasmid #38277
pEGFP-N1	Clontech	Cat#: 6085-1
pEGFP-N1-TFEB	Roczniak-Ferguson et al., 2012	Addgene plasmid #38119
pEGFP-N1-TFE3	Roczniak-Ferguson et al., 2012	Addgene plasmid #38120
pEGFP-N1-MITF-A	Roczniak-Ferguson et al., 2012	Addgene plasmid #38132
pEGFP-Q74	Narain et al., 1999	Addgene plasmid # 40262
pLVX-TetOne-Puro	Clontech	Cat#: 631849
pLVX-TetOne-Puro-GFP-HTT exon1 Q74	This paper	N/A
lentiCRISPR v2	Sanjana et al., 2014	Addgene plasmid #52961
lentiCRISPR v2-human hMOF/KAT8 #1	This paper	N/A
lentiCRISPR v2-human hMOF/KAT8 #2	This paper	N/A
lentiCRISPR v2-human SIRT1	This paper	N/A
lentiCRISPR v2-human AMPKα1/PRKAA1	This paper	N/A
lentiCRISPR v2-human AMPKα2/PRKAA2	This paper	N/A
lentiCRISPR v2-human ATG5	This paper	N/A
lentiCRISPR v2-non-targeting control (NTC)	This paper	N/A
pLKO.1-non-targeting control (NTC)	Sigma-Aldrich	Cat#: SHC002
pLKO.1-human G9a shRNA #1	Dharmacon	Cat#: TRCN0000115670

(*Continued on next page*)

Continued

REAGENT or RESOURCE	SOURCE	IDENTIFIER
pLKO.1-human G9a shRNA #2	Dharmacon	Cat#: TRCN0000115668
pTRIPZ-non-targeting control (NTC)	Dharmacon	Cat#: RHS4743
pTRIPZ-human BRD4 shRNA (targeting short and long variants)	Dharmacon	Cat#: V3THS326487
Software and Algorithms		
ImageJ64	NIH	https://imagej.nih.gov/ij/
CellProfiler	Anne Carpenter Lab (Broad Institute)	http://cellprofiler.org
Optimized CRISPR Design	Feng Zhang Lab (MIT)	http://crispr.mit.edu
FastQC	Babraham Bioinformatics	https://www.bioinformatics.babraham.ac.uk/projects/fastqc/
FastQ Screen	Babraham Bioinformatics	http://www.bioinformatics.babraham.ac.uk/projects/fastq_screen/
TopHat2 v.2.0.10	Kim et al., 2013	https://ccb.jhu.edu/software/tophat/index.shtml
Bowtie v.2.1.0	Langmead and Salzberg, 2012	http://bowtie-bio.sourceforge.net/bowtie2/index.shtml
HTSeq v.0.5.4p3	Simon Anders (EMBL Heidelberg)	http://www-huber.embl.de/users/anders/HTSeq/doc/overview.html
DESeq2	Love et al., 2014	N/A
g:Profiler	Reimand et al., 2011	N/A
ZEN 2010 software	Zeiss	N/A
ZEN 2012 software	Zeiss	N/A
StepOne software	Applied Biosystems	N/A
FlowJo software v.7.6.5	FlowJo	N/A
BD CellQuest Pro software	BD Biosciences	N/A
GraphPad Prism 7	GraphPad software	N/A

CONTACT FOR REAGENT AND RESOURCE SHARING

Further information and requests for resources and reagents should be directed to and will be fulfilled by the Lead Contact, Kevin M. Ryan (k.ryan@beatson.gla.ac.uk).

EXPERIMENTAL MODEL AND SUBJECT DETAILS

Cell Culture

KP-4 cells (RIKEN, Cat#: RCB1005) were cultured in IMDM (Thermo Fisher Scientific, Cat#: 21980065) supplemented with 20% FBS (Thermo Fisher Scientific, Cat#: 10270106) and antibiotics (Thermo Fisher Scientific, Cat#: 15140122) in a humidified atmosphere with 5% CO_2. PA-TU-8902 (DMSZ, Cat#: ACC-179), SUIT2 (JCRB, Cat#: JCRB1094), PA-TU-8988T (DMSZ, Cat#: ACC-162), hTERT-HPNE (ATCC, Cat#: CRL-4023), IMR90 ER-HRas G12V (a gift from Peter D. Adams, Cancer Research UK Beatson Institute, UK), and HEK293T (ATCC, Cat#: CRL-3216) cells were maintained in DMEM (Thermo Fisher Scientific, Cat#: 21969035) supplemented with 10% FBS, 2 mM L-Glutamine (Thermo Fisher Scientific, Cat#: 25030032), and antibiotics. PK-1 (RIKEN, Cat#: RCB1972) and TY-82 (JCRB, Cat#: JCRB1330) were maintained in RPMI-1640 (Thermo Fisher Scientific, Cat#: 31870074) supplemented with 10% FBS, 2 mM L-Glutamine, and antibiotics. For starvation experiments, cells were cultured in EBSS (Sigma-Aldrich, Cat#: E2888). Since amino acid-free and glucose-free IMDM media are not commercially available, we used amino acid-free and glucose-free DMEM media supplemented with 20% FBS. For amino acid starvation, DMEM low glucose amino acid free (USBiological, Cat#: D9800-13) was supplemented with Glucose (Thermo Fisher Scientific, Cat#: 15023021) to 25 mM, 20% dialized FBS (Thermo Fisher Scientific, Cat#: 26400044), 1 mM Sodium pyruvate (Sigma-Aldrich, Cat#: S8636), 3.7 g/L Sodium bicarbonate (Sigma-Aldrich, Cat#: S5761), and antibiotics. For glucose starvation, DMEM no glucose (Thermo Fisher Scientific, Cat#: 11966-025) was supplemented with 20% dialized FBS, 1 mM Sodium pyruvate, and antibiotics. DMEM supplemented with 20% dialized FBS, 2 mM L-Glutamine, and antibiotics was used as a control for amino acid and glucose starvation.

Mice

6-8 week old TRE-shRen/CAG-rtTA3 and TRE-shBRD4/CAG-rtTA3 mice were fed with 625 mg/kg doxycycline-containing food pellets (Harlan Teklad) for 2 weeks. Tissues were harvested and fixed overnight in 10% neutral buffered formalin, followed by paraffin embedding and sectioning as described previously (Bolden et al., 2014). All experimental procedures were approved by, and adhered to guidelines of, the Memorial Sloan Kettering Cancer Center institutional animal care and use committee.

METHOD DETAILS

Reagents

Chloroquine (Cat#: C6628), Doxycycline (Cat#: D9891), Antimycin A (Cat#: A8674), Oligomycin (Cat#: O4876), D-(+)-Trehalose dihydrate (Cat#: T0167), and 4-Hydroxytamoxifen (Cat#: H7904) were from Sigma-Aldrich. (+)-JQ1 (Cat#: 4499) and I-BET151 (Cat#: 4650) were from TOCRIS. OTX015 (Cat#: 15947) was from Cayman. Compound C (Cat#: 171264) was from EMD Millipore. Rapamycin (Cat#: R-5000) was from LC Laboratories. ARV-825 was from Chemietek (Cat#: CT-ARV825).

Plasmid Transfection

Plasmids were transfected into HEK293T cells using Lipofectamine2000 (Thermo Fisher Scientific, Cat#: 11668027) or GeneJuice (EMD Millipore, Cat#: 70967). KP-4 cells were transfected with plasmids using Lipofectamine3000 (Thermo Fisher Scientific, Cat#: L3000015).

siRNA Transfection

Cells were reverse-transfected with 20 nM of siRNAs using Lipofectamine RNAiMAX reagent (Thermo Fisher Scientific, Cat#: 13778150) for 72 hr. siRNAs are listed in Table S1. BRD4 #1-#4 were used to knockdown both short and long isoforms. Since NUT is a testis-specific gene, NUT siRNAs were used to knockdown BRD4-NUT fusion gene (Schwartz et al., 2011).

Western Blotting

Cells were lysed with cell lysis buffer (20 mM HEPES-KOH pH7.5, 150 mM NaCl, 2 mM EDTA, 1% Triton X-100) containing Halt protease inhibitor cocktail (Thermo Scientific, Cat#: 78430). Total protein concentration was determined by BCA assay using Copper (II) sulfate solution (Sigma-Aldrich, Cat#: C2284) and Bicinchoninic Acid solution (Sigma-Aldrich, Cat#: B9643). The cell extracts were mixed with 6x SDS-PAGE sample buffer (0.3 M Tris-HCl (pH6.8), 0.12 g/ml SDS, 0.1 M Dithiothreitol, 60% Glycerol, 0.6 mg/ml Bromophenol blue) and heated at 99°C for 5 min. The same amount of protein (10-30 μg) was loaded and run on SDS-PAGE. The following antibodies were used. BRD4 E2A7X (long isoform) (Cat#: 13440S, WB 1/1000, IP, ChIP), NUT (Cat#: 3625S, WB 1/1000), G9a (Cat#: 3306S, WB 1/1000), SIRT1 (Cat#: 9475P, WB 1/1000), DBC1 (Cat#: 5857S, WB 1/1000, IP), AMPKα pT172 (Cat#: 2535S, WB 1/1000), AMPKα1/α2 (Cat#: 2793S, WB 1/1000), LC3B (Cat#: 2775S, WB 1/1500), LC3B D11 (Cat#: 3868S, IF 1/200), TFEB (Cat#: 4240S, WB 1/1000), LAMP1 (Cat#: 9091P, WB 1/1000, IF 1/200), CTSD (Cat#: 2284S, WB 1/1000), ASM (Cat#: 3687S, WB 1/1000), CTSB (Cat#: 31718S, WB 1/1000), ATG5 (Cat#: 12994S, WB 1/1500), p70 S6K pT389 (Cat#: 9234S, WB 1/1000), p70 S6K (Cat#: 2708S, WB 1/1500), Histone H4K16Ac (Cat#: 13534S, WB 1/1000), Histone H4 (Cat#: 2935P, WB 1/1000), c-Myc (Cat#: 5605S, WB 1/1000), CDK9 (Cat#: 2316T, WB 1/1000), GFP (Cat#: 2956S, WB 1/2000), TFE3 (Cat#: 14779S, WB 1/1000), Normal rabbit IgG (Cat#: 2729S, IP, ChIP), Mouse IgG1 isotype control (Cat#: 5415S, IP), Anti-rabbit IgG HRP-linked Antibody (Cat#: 7074S, WB 1/5000), and Anti-mouse IgG HRP-linked Antibody (Cat#: #7076S, WB 1/5000) were from Cell Signaling Technology. BRD4 NT (short and long isoforms) (Cat#: ab128874, WB 1/1000), GAPDH (Cat#: ab9484, WB 1/2000), β-Actin (Cat#: ab8227, WB 1/2000), G9a (Cat#: ab133482, WB 1/1000, IP, ChIP), H3K9diMe (Cat#: ab1220, ChIP), GAA (Cat#: ab137068, WB 1/1000), Membrane Integrity WB Antibody Cocktail (Cat#: ab110414 (MS620), WB 1/5000), and Anti-Goat IgG H&L (HRP) (Cat#: ab6741, WB 1/5000) were from Abcam. BRD4 (long isoform) (Cat#: A301-985A50, ChIP, IHC 1/2000), BRD2 (Cat#: A302-583A, WB 1/5000), BRD3 (Cat#: A302-368A, WB 1/5000), and hMOF (Cat#: A300-992A, WB 1/1000) were from Bethyl Laboratories. H4K16Ac (Cat#: 07-329, WB 1/2000, ChIP) was from Millipore. H4K16Ac (Cat#: 39167, ChIP) was from Active motif. LAMP2/CD107b (Cat#: 555803, WB 1/2000) and p62 (Cat#: 610833, WB 1/5000) were from BD. GFP (Cat#: MMS-118P, WB 1/5000) was from Covance. LaminA/C (Cat#: sc-6215, WB 1/2000) and Hsp90β (Cat#: sc-1057, WB 1/2000) were from Santa Cruz. FLAG (Cat#: F1804, WB 1/2000) was Sigma-Aldrich. V5 (Cat#: 46-0705, WB 1/2000) was from Invitrogen. WIPI2 (Cat#: MCA5780GA, IF 1/200) was from Bio-Rad. LC3B (Cat#: 0231-100/LC3-5F10, IHC: 1/50) was from NanoTools. Proteins were detected using Pierce ECL Western Blotting Substrate (Thermo Fisher Scientific, Cat#: 32106) or SuperSignal West Femto Maximum Sensitivity Substrate (Thermo Fisher Scientific, Cat#: 34095). Signal intensity was measured using ImageJ64 software (https://imagej.nih.gov/ij/).

Plasmids, sgRNAs, and shRNAs

cDNA encoding human BRD4 transcript variant long was excised from pRetrox-tight-puro-HA-BRD4 (obtained from MRC Protein Phosphorylation and Ubiquitylation Unit, Cat#: DU46347) and inserted into pBabe-puro (a gift from Hartmut Land & Jay Morgenstern & Bob Weinberg, Addgene plasmid # 1764) (Morgenstern and Land, 1990) and pLenti-puro (a gift from Ie-Ming Shih,

Addgene plasmid # 39481) (Guan et al., 2011) vectors. Human BRD4 transcript variant short was cloned using pRetrox-tight-puro-HA-BRD4 as a template with BRD4 Short Fw (CGCGATATCACCATGGACTACAAAGACGATGACGACAAGATGTCTGCGGAG AGCGG) and BRD4 Short Rv (CGCGTCGACTTAGGCAGGACCTGTTTCGGAGTCTTCGCTG) primers. The fragment was digested with EcoRV and SalI and inserted into pcDNA3 vector (Invitrogen) (EcoRV and XhoI sites). The sequence was confirmed to be identical to BRD4 transcript variant short (RefSeq: NM_014299.2). mRFP-GFP-LC3 cDNA was excised from pEGFP-C1+mRFP-LC3 (a kind gift from Tamotsu Yoshimori, Osaka University, Japan) (Kimura et al., 2007) and inserted into pBabe-puro vector. pLZRS-YFP-Parkin was described in (Baudot et al., 2015). pLenti6-MK1-EHMT2 (G9a)-V5 was a gift from Bernard Futscher (Addgene plasmid # 31113). pTreTight-Htt94Q-CFP was a gift from Nico Dantuma (Addgene plasmid #23966) (Maynard et al., 2009). pMA2640 was a gift from Mikhail Alexeyev (Addgene plasmid #25434) (Alexeyev et al., 2010). pFlag-CMV2-Brd4 (1-1362) was a gift from Eric Verdin (Addgene plasmid #22304) (Bisgrove et al., 2007). psPAX2 and pMD2.G were gifts from Didier Trono (Addgene plasmid #12260 and #12259). pMXs-puro GFP-p62 was a gift from Noboru Mizushima (Addgene plasmid #38277) (Itakura and Mizushima, 2011). pEGFP-N1-TFEB, pEGFP-N1-TFE3 and pEGFP-N1-MITF-A were gifts from Shawn Ferguson (Addgene plasmid #38119, #38120 and #38132) (Roczniak-Ferguson et al., 2012). GFP-HTT exon1 Q74 cDNA was excised from pEGFP-Q74 (a gift from David Rubinsztein, Addgene plasmid #40262) (Narain et al., 1999) and inserted into pLVX-TetOne-puro vector (Clontech, Cat#: 631849). pTRE-tight and pEGFP-N1 vectors were from Clontech (Cat#: 631059 and 6085-1). lentiCRISPR v2 was a gift from Feng Zhang (Addgene plasmid #52961) (Sanjana et al., 2014).

The following sgRNA sequences were used in the experiments.

Human hMOF/KAT8 #1: CCTTCCCGCGATGGCGGCAC (Wang et al., 2014);
Human hMOF/KAT8 #2: GGCGGCACAGGGAGCTGCTG (Wang et al., 2014);
Human SIRT1: AGAGATGGCTGGAATTGTCC (Wang et al., 2014);
Human AMPKα1/PRKAA1: AAGATCGGCCACTACATTCT (Wang et al., 2014);
Human AMPKα2/PRKAA2: GCTGAGAAGCAGAAGCACGA (Wang et al., 2014);
Human ATG5: AAGAGTAAGTTATTTGACGT (Designed using Optimized CRISPR Design (http://crispr.mit.edu);
Non-targeting control (NTC): GTAGCGAACGTGTCCGGCGT (Wang et al., 2014).

pLKO.1-non-targeting control (NTC) (Sigma-Aldrich, Cat#: SHC002) and pLKO.1-shG9a #1 and #2 (Dharmacon, Cat#: TRCN0000115670 (#1) and TRCN0000115668 (#2)) were purchased from Sigma-Aldrich and Dharmacon, respectively. pTRIPZ-non-targeting control (NTC) (Cat#: RHS4743) and shBRD4 (Cat#: V3THS326487) were purchased from Dharmacon.

Lentivirus and Retrovirus Production and Infection

Lentiviral plasmids were transfected into HEK293T cells together with packaging and envelope plasmids (psPAX2 and pMD2.G) using Lipofectamine2000 (Thermo Fisher Scientific, Cat#: 11668027) or Genejuice (EMD Millipore, Cat# 70967). At 2 days after transfection, the medium was passed through a 0.45 μm pore filter and mixed with Polybrene (Sigma-Aldrich, Cat#: H9268). The medium containing lentiviruses was transferred to the recipient cells. HEK293T cells were further cultured in fresh medium for 24 hr. After 6 hr of infection, medium was changed. Next day, infection was repeated as above. After lentivirus infection, cells were selected with 1 μg/ml of Puromycin (Sigma-Aldrich, Cat#: P9620) or 5 μg/ml of Blasticidin (Thermo Fisher Scientific, Cat# R21001) for 5-10 days. For retrovirus production, retroviral plasmids were transfected into Phoenix-AMPHO cells (ATCC, Cat#: CRL-3213) using Lipofectamine2000 or Genejuice. Retrovirus infection was carried out as described above.

Immunofluorescence and Confocal Imaging

Cells seeded on coverslips (VWR 16mm, Thickness No.1, Cat# 631-0152) were fixed with 4% PFA/PBS (Electron microscopy science, Cat#: 1570) at RT for 15 min followed by permeabilization with 0.1% Triton X-100/PBS. For LC3 and WIPI2 staining, cells were fixed and permeabilized in 100% ice-cold methanol at -20°C for 15min. After incubation in blocking solution (3% BSA/PBS) at room temperature for 1hr, cells were incubated with primary antibody at 4°C overnight. Cells were washed in PBS three times and stained with 2 μg/ml Hoechst33342 (Thermo Fisher Scientific, Cat#: H3570) for 15min at room temperature, followed by wash in PBS three times. Cells were then incubated with Anti-rabbit IgG-Alexa 488 (Thermo Fisher Scientific, Cat#: A11008, 1/1000) or Anti-mouse IgG-Alexa 568 (Thermo Fisher Scientific, Cat#: A11031, 1/1000) for 1hr at room temperature. After cells were washed with PBS four times, the coverslips were mounted onto glass slides using Fluorescent Mounting Medium (DAKO, Cat#: S3023). To visualize acidic lysosome compartments, cells were stained with LysoTracker Red DND-99 (Thermo Fisher Scientific, Cat# L7528, 100 nM, 2 hr). To measure lysosomal Cathepsin B activity, cells were incubated with Magic Red CathepsinB (ImmunoChemistry Technologies, Cat#: 938) for 1 hr according to the manufacturer's instructions. After incubation with LysoTracker Red or Magic Red CathepsinB, cells were fixed in 4% PFA/PBS for 15 min at room temperature. Cells were then washed in PBS five times followed by staining with 2 μg/ml Hoechst33342 for 15 min at room temperature. Cells were washed with PBS four times and the coverslips were mounted onto glass slides using Fluorescent Mounting Medium. All confocal images were acquired and processed using a Zeiss 710 confocal microscope (Zeiss) and Zen2010 software (Zeiss). The number of LC3 and WIPI2 puncta were counted using

CellProfiler software (http://cellprofiler.org) and normalized to the number of nuclei. The area of LAMP-1-, LysoTracker Red-, and Magic Red CathepsinB-positive compartments was measured using ImageJ64 software (https://imagej.nih.gov/ij/) and normalized to the number of nuclei.

Immunohistochemistry

Transgenic mice harboring inducible Renilla luciferase and BRD4 shRNAs whose expression is under the control of TRE promoter were generated as described previously using the same shRNA sequences (Bolden et al., 2014). Briefly ESCs containing a homing cassette at the Col1a1 locus were targeted with TRE-driven single-copy shRNAs and mice were generated by blastocytst injections. Resulting F1 mice were crossed to the CAG-rtTA3 strain (Premsrirut et al., 2011). Doxycycline was administered to 6-8 week old TRE-shRen/CAG-rtTA3 or TRE-shBRD4/CAG-rtTA3 mice via 625 mg/kg doxycycline-containing food pellets (Harlan Teklad) for 2 weeks. JQ1 preparation and administration were performed as described previously (Bolden et al., 2014). JQ1 powder was dissolved in DMSO to generate a concentrated 50 mg/mL stock solution. For administration to animals, a working solution was generated by diluting 1 part of the concentrated JQ1stock drop-wise into 9 parts 10% 2-(Hydroxypropyl)-β-cyclodextrin (Sigma-Aldrich, Cat#: C0926). C57BL/6 mice received once daily intraperitoneal injections of 100 mg/kg JQ1 for 2 weeks. Tissues were harvested and fixed overnight in 10% neutral buffered formalin, followed by paraffin embedding and sectioning as described previously (Bolden et al., 2014). All experimental procedures were approved by, and adhered to guidelines of, the Memorial Sloan Kettering Cancer Center institutional animal care and use committee.

Paraffin embedded sections were placed in Xylene for 5 min, 100% Ethanol for 1 min twice, 70% Ethanol for 1 min, and deionized water for 5 min. Antigen retrieval was performed in PT Module using Sodium citrate retrieval buffer pH 6 (Thermo Scientific, Cat#: TA-250-PM1X) at 98°C for 25 min followed by wash with Tris buffered saline and tween 20 (TBST) (Thermo Scientific, Cat#: TA-999-TT). The sections were then blocked for endogenous peroxidase using Peroxidase-blocking solution (Dako, Cat#: S2023) for 5 min followed by wash with TBST. The sections were incubated with LC3B (NanoTools, Cat#: 0231-100/LC3-5F10, 1/50) or BRD4 (Bethyl Laboratories, Cat#: A301-985A50, 1/2000) antibody diluted in Antibody diluent (Dako, Cat#: S2022) for 35 min. After wash with TBST twice, the sections were incubated with EnVision+ HRP, Mouse or Rabbit (Dako, Cat#: K4001 and K4003) for 30 min followed by wash with TBST twice. The sections were incubated with 3,3′-Diaminobenzidine tetrahydrochloride (DAB) (Dako, Cat#: K3468) for 10 min, washed with deionized water for 1 min, stained with Haematoxylin Z (CellPath, Cat#: RBA-4201-00A) for 7 min, washed with deionized water for 1min, differentiated in 1% Acid alcohol, washed with deionized water for 30 s, incubated in Scott's tap water substitute for 1 min, and washed with deionized water for 1 min. The sections were dehydrated, cleaned, and mounted with DPX. Images were acquired using a Zeiss Axio Scope.A1 microscope (Zeiss) and ZEN 2012 software (Zeiss).

To measure LC3 lipidation levels in BRD4 knockdown and JQ1-treated mice, proteins were extracted from formalin-fixed paraffin-embedded (FFPE) tissues using Qproteome FFPE Tissue Kit (QIAGEN, Cat#: 37623). Total protein concentration was determined by Bradford assay (Bio-Rad, Cat#: 500-0201). The cell extracts were mixed with 6x SDS-PAGE sample buffer and heated at 99°C for 5 min. The same amount of protein (20-30 μg) was loaded and run on SDS-PAGE. To confirm BRD4 knockdown, total RNA was isolated from FFPE tissues using RNeasy FFPE kit (QIAGEN, Cat#: 73504) followed by RT-qPCR analysis described below.

RNA Sequencing

KP-4 cells were transfected with Control #1, BRD4 #1, or BRD4 #2 siRNA for 72 hr. At 72 hr after transfection, total RNA was isolated and purified using RNeasy mini kit (QIAGEN, Cat#: 74104). Quality of the purified RNA was assessed using an Agilent RNA 6000 Nano kit and 2100 Bioanalyzer. Libraries for cluster generation and DNA sequencing were prepared following an adapted method from (Fisher et al., 2011) using TruSeq RNA Sample Prep Kit v2 (Illumina, Cat#: RS-122-2001). Quality and quantity of the DNA libraries were assessed on an Agilent 2100 Bioanalyser and Qubit (Thermo Fisher Scientific), respectively. The libraries were run on the Illumina Next Seq 500 using the High Output 75 cycles kit (2x36 cycles, paired end reads, single index). The results were then analyzed as follows. Quality checks on the raw RNA-Seq data files were conducted using fastqc (https://www.bioinformatics.babraham.ac.uk/projects/fastqc/) and fastq_screen (http://www.bioinformatics.babraham.ac.uk/projects/fastq_screen/). RNA-Seq reads were aligned to the GRCh37 (Church et al., 2011) version of the human genome using tophat2 version 2.0.10 (Kim et al., 2013) with Bowtie version 2.1.0 (Langmead and Salzberg, 2012). Expression levels were determined and statistically analyzed by a combination of HTSeq version 0.5.4p3 (http://www-huber.embl.de/users/anders/HTSeq/doc/overview.html), the R 3.1.1 environment, utilizing packages from the Bioconductor data analysis suite and differential gene expression analysis based on a generalized linear model using the DESeq2 (Love et al., 2014). Enrichment analysis for Gene Ontology terms within this gene set was performed using g:Profiler (Reimand et al., 2011).

RNA Isolation, Reverse Transcription, and Quantitative PCR (RT-qPCR)

The total RNA was isolated and purified using the RNeasy mini kit (QIAGEN, Cat#: 74104). 1 μg of total RNA was reverse-transcribed using the High-Capacity RNA-to-cDNA Kit (Applied Biosystems, Cat# 4387406) at 37°C for 1 hr. 1 μL of cDNA from 20 μL reaction volume was used for qPCR. qPCR was run on a StepOnePlus (Applied Biosystems) using Fast SYBR Green Master Mix (Applied Aiosystems, Cat#: 4385617). mRNA levels were determined by the relative standard curve method, normalized to 18S, GAPDH, or HPRT levels, and presented as relative mRNA levels. qPCR analyses were done in triplicate. Experiments were repeated at least twice. Primers are listed in Table S2.

Co-immunoprecipitation

Cells were lysed in cell lysis buffer (20 mM HEPES-KOH pH 7.5, 150 mM NaCl, 2 mM EDTA, 1% Triton X-100) containing Halt protease inhibitor cocktail (Thermo Fisher Scientific, Cat#: 78430). Lysates were incubated with 1 μg of antibody or control rabbit or mouse IgG (Cell Signaling Technology, Cat#: 2729S and 5415S) at 4°C overnight followed by incubation with 50 μL of Dynabeads Protein G (Thermo Fisher Scientific, Cat# 10004D) for 3 hr. After washing 3 times with cell lysis buffer containing 0.05% NP-40, immunoprecipitates were resuspended in 1x SDS-PAGE sample buffer and resolved by SDS-PAGE followed by western blot analysis.

Chromatin Immunoprecipitation

Approximately 7×10^6 cells were fixed with 1% Formaldehyde (Sigma-Aldrich, Cat#: F8775) at room temperature for 10 min and quenched by adding Glycine at a final concentration of 125 mM. Cells were then harvested and lysed in 500 μL of ChIP lysis Buffer (50 mM Tris-HCl pH8.0, 5 mM EDTA, 150 mM NaCl, 0.5% Triton X-100, 0.5% SDS, 0.5% NP-40, 1 mM Sodium butylate) containing Halt protease inhibitor cocktail. The lysates were subjected to sonication to shear DNA to the length of approximately between 150 and 900 bp using a Bioruptor (Diagenode). 300 μL of the lysate were then diluted in 1.2 mL of ChIP dilution buffer (50 mM Tris-HCl pH8.0, 5 mM EDTA, 150 mM NaCl, 0.5% Triton X-100, 0.5% NP-40, 1 mM Sodium butylate) containing Halt protease inhibitor cocktail, and incubated with control IgG (Cell Signaling Technology, Cat#: 2729S) or primary antibody together with 50 μL of Dynabeads protein G (Thermo Fisher Scientific, Cat# 10004D) at 4°C overnight. The beads were washed sequentially with the following buffers: ChIP dilution buffer, high salt wash buffer twice (50 mM Tris-HCl pH8.0, 5 mM EDTA, 600 mM NaCl, 0.5% Triton X-100, 0.5% NP-40), LiCl wash buffer twice (10 mM Tris-HCl pH 8.0, 250 mM LiCl, 1 mM EDTA, 0.5% NP-40, 0.5% sodium deoxycholate), ChIP dilution buffer, and wash buffer 4 (10 mM Tris-HCl, pH 8.0, 0.1% NP-40). The immunocomplexes were eluted with 75 μL of elution buffer (1% SDS, 0.1 M $NaHCO_3$) twice at 65°C for 30 min. After elution, the cross-link was reversed by adding NaCl to a final concentration of 200 mM and incubated together with Proteinase K (Thermo Fisher Scientific, Cat#: EO0491) for 6 hr at 65°C. 3M Sodium acetate solution pH5.2 (Thermo Fisher Scientific, Cat#: R1181) was added to the eluate to lower pH. DNA fragments were purified with the QIAquick PCR purification kit (QIAGEN, Cat#: 28104). The purified DNA was analyzed on a StepOnePlus using Fast SYBR Green Master Mix. The results are presented as percentage of input. qPCR analyses were done in triplicate. Experiments were repeated at least twice. Primers are listed in Table S2.

RNAi in *Drosophila* S2R$^+$ cells

Culture of *Drosophila* S2R$^+$ cells, generation of S2R$^+$ cells stably expressing GFP-LC3, delivery of control luciferase and Fs(1)h dsRNAs into GFP-LC3 expressing S2R$^+$ cells, and subsequent confocal microscopic analysis were described previously (Wilkinson et al., 2011).

β-Hexosaminidase Assay

Lysosomal β-Hexosaminidase activity was measured as described in (Chauhan et al., 2013). An equal number of KP-4 cells (3×10^5 cells) were lysed in 50 μL of 0.1% Triton X-100 containing Halt protease inhibitor cocktail. 20 μL of the cell extracts were then incubated with 1 mM 4-Nitrophenyl N-acetyl-β-D-glucosaminide (p-NAG) (Sigma-Aldrich, Cat#: N9376) at 37°C for 1.5 hr. The reaction was stopped by adding 0.1 M Carbonate/bicarbonate (Sigma-Aldrich, Cat#: C3041). The amount of the reaction product was measured by reading the absorbance at 405nm.

Aggrephagy

Effects of BRD4 knockdown and overexpression on aggrephagy were examined as described in (Bauer et al., 2010). KP-4 cells harboring rtTA and Tre-tight-HTT Q94-CFP were treated with 1 μg/ml of Doxycycline (DOX) for 10 hr. Cells were then washed with PBS three times and cultured in fresh medium. At 48 hr after removal of DOX, cells were lysed in cell lysis buffer and separated into Triton X-100 soluble and insoluble fractions. Triton X-100 insoluble fraction was washed with lysis buffer three times. Triton X-100 soluble fraction was mixed with 6x SDS-PAGE sample buffer and Triton X-100 insoluble fraction was resuspended in 1x SDS-PAGE sample buffer followed by SDS-PAGE and western blot. To determine the effect of mutant HTT on cell number, HTT exon1 Q74 was overexpressed in cells as described in (Williams et al., 2008). KP-4 cells infected with pLVX-TetOne-GFP-HTT Q74 and control parental cells were treated with 100 ng/ml DOX for 60 hr. Cell number of mutant HTT expressing cells was normalized to that of parental cells and presented as percentage of reduction in cell number upon mutant HTT induction. Cell number of KP-4 pLVX-GFP-HTT Q74 and parental control cells was comparable in the absence of DOX (data is shown in Figure S6K first lane).

Mitophagy

Effects of BRD4 knockdown and overexpression on mitophagy were examined as described in (Baudot et al., 2015). KP-4 cells stably expressing YFP-parkin were treated with 1 μM of Antimycin A and Oligomycin for 8 hr. Degradation of mitochondrial proteins was monitored as a readout for mitophagy.

Xenophagy

KP-4 cells were plated in triplicates at 8×10^4 cells in 6-well plates and reverse-transfected with 20 nM of siRNAs using Lipofectamine RNAiMAX. Infection with *Salmonella enterica* serovar Typhimurium (strain 12023) was performed 48 hr after siRNA transfection as

described previously (McEwan et al., 2015b). Therefore, an overnight (stationary) culture of *Salmonella* was diluted 1:33 and incubated for 3 hr at 37°C prior to infection. The culture was diluted 1:250 to infect cells and *Salmonella* were allowed to invade cells for 15 min. Afterward, cells were washed with EBSS and incubated for 1 hr in media containing 100 μg/ml gentamycin. Media was replaced with 20 μg/ml gentamycin thereafter. To enumerate intracellular *Salmonella*, cells were lysed 2, 6 or 8 hr post infection in PBS with 0.1% Triton X-100. Lysates were serial diluted and plated in duplicates on Agar plates.

Cell Proliferation and Cell Death Assays

For EdU staining, KP-4 cells were treated with 10 μM EdU for 2 hr before fixation. Cells were then subjected to EdU staining using Click-iT EdU Flow Cytometry Assay Kit (Thermo Fisher Scientific, Cat#: C10633). Samples were then stained with FxCycle PI/RNAase staining solution (Thermo Fisher Scientific, Cat#: F10797) and analyzed on a FACSCalibur (BD Biosciences). Percentage of EdU positive cells was calculated using FlowJo software. Cell number was determined by using a CASY cell counter (Roche Innovatis) or by Trypan blue exclusion test using Trypan blue solution (Sigma-Aldrich, Cat#: T8154). To determine the sub G1 population, cells were fixed with 10% methanol followed by staining with 50 μg/ml Propidium iodide (Sigma-Aldrich, Cat#: P4170) containing 50 μg/ml RNase A (QIAGEN, Cat#: 19101). Cells were then analyzed on a FACSCalibur using BD CellQuest Pro software (BD Biosciences).

QUANTIFICATION AND STATISTICAL ANALYSIS

Quantification of Western Blotting

Quantification of western blotting was performed using ImageJ64 software (https://imagej.nih.gov/ij/) using Gel analyzer script. Signal intensity of the protein of interest was normalized to that of loading control (GAPDH, Hsp90, or β-actin).

Quantification of Microscopic Images

The number of LC3 and WIPI2 puncta were counted using CellProfiler software (http://cellprofiler.org) and normalized to the number of nuclei. The area of LAMP-1-, LysoTracker Red-, and Magic Red CathepsinB-positive compartments was measured using ImageJ64 software (https://imagej.nih.gov/ij/) and normalized to the number of nuclei.

Quantification of the qPCR Results

Target mRNA levels were determined by relative standard curve method, normalized to 18S, GAPDH, or HPRT levels, and presented as relative mRNA levels compared to control. StepOne software (Applied Biosystems) was used to analyze the data.

Statistical Analyses

All studies were performed on at least three independent occasions. Results are shown as mean ± SD unless mentioned otherwise. Statistical significance was determined by two-tailed unpaired Student's t test for two group comparison and one-way ANOVA with Tukey or Dunnett for multiple group comparison using GraphPad Prism 7 (GraphPad software). P values < 0.05 were considered significant. Significance in all figures is indicated as follows: * $p < 0.01$, ** $p < 0.05$, N.S.: no significance.

DATA AND SOFTWARE AVAILABILITY

The raw and processed data of the RNA-Seq have been deposited in Gene Expression Omnibus under GEO: GSE90444. Full scans of western blot data and original microscopy images have been deposited in Mendeley Data (http://dx.doi.org/10.17632/ksz4pmwkdb.1).

Molecular Cell, Volume *66*

Supplemental Information

Bromodomain Protein BRD4

Is a Transcriptional Repressor

of Autophagy and Lysosomal Function

Jun-ichi Sakamaki, Simon Wilkinson, Marcel Hahn, Nilgun Tasdemir, Jim O'Prey, William Clark, Ann Hedley, Colin Nixon, Jaclyn S. Long, Maria New, Tim Van Acker, Sharon A. Tooze, Scott W. Lowe, Ivan Dikic, and Kevin M. Ryan

Figure S1

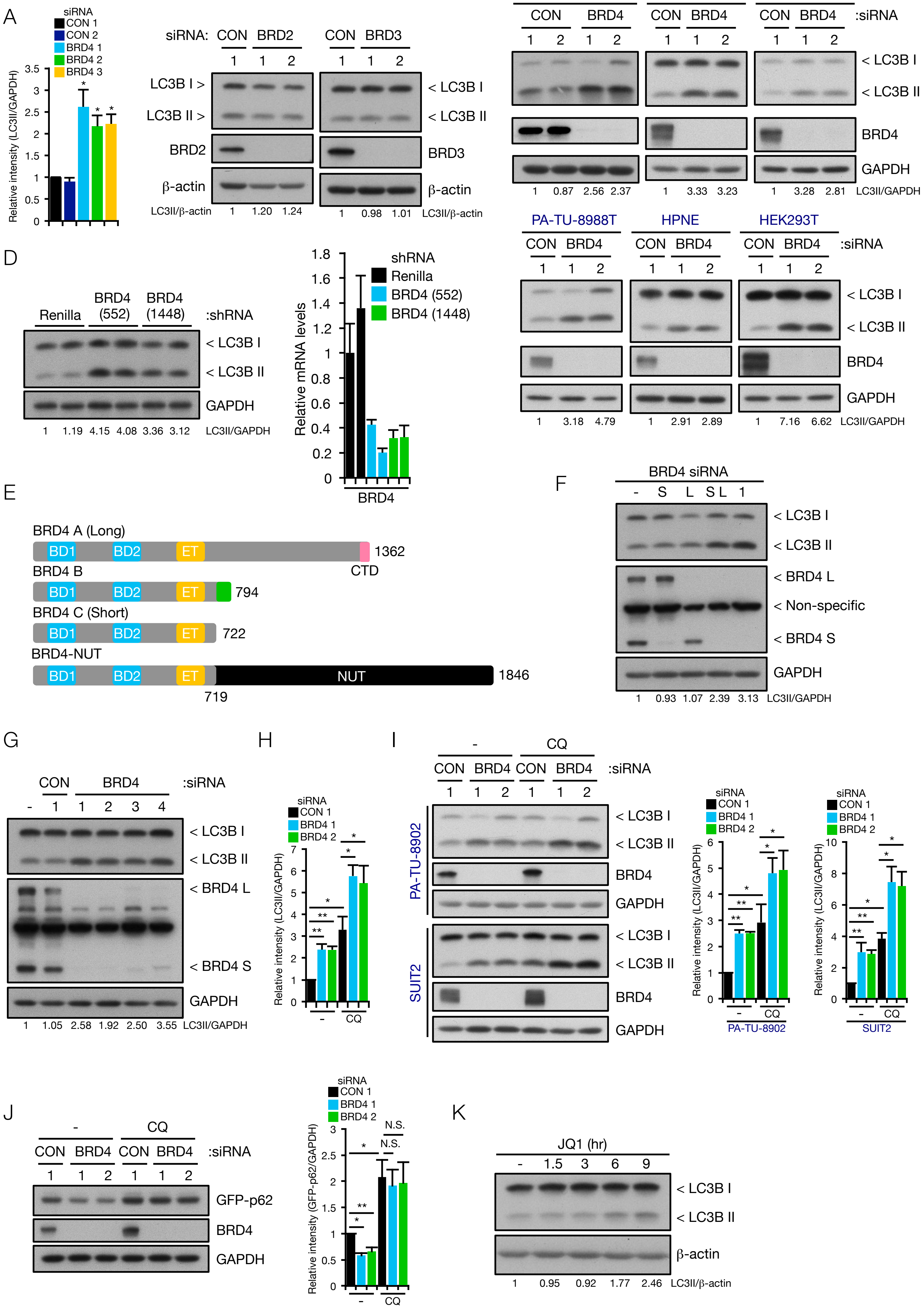

A
siRNA
CON 1
CON 2
BRD4 1
BRD4 2
BRD4 3
Relative intensity (LC3II/GAPDH)
B
siRNA: CON BRD2 CON BRD3
LC3B I >
LC3B II >
BRD2
β-actin
< LC3B I
< LC3B II
BRD3
β-actin
LC3II/β-actin 1 1.20 1.24
1 0.98 1.01 LC3II/β-actin
C
PA-TU-8902
SUIT2
PK-1
CON BRD4 CON BRD4 CON BRD4 :siRNA
< LC3B I
< LC3B II
BRD4
GAPDH
1 0.87 2.56 2.37 1 3.33 3.23 1 3.28 2.81 LC3II/GAPDH
PA-TU-8988T
HPNE
HEK293T
CON BRD4 CON BRD4 CON BRD4 :siRNA
1 3.18 4.79 1 2.91 2.89 1 7.16 6.62 LC3II/GAPDH
D
Renilla
BRD4 (552)
BRD4 (1448)
:shRNA
< LC3B I
< LC3B II
GAPDH
1 1.19 4.15 4.08 3.36 3.12 LC3II/GAPDH
shRNA
Renilla
BRD4 (552)
BRD4 (1448)
Relative mRNA levels
BRD4
E
BRD4 A (Long)
BD1 BD2 ET
1362
CTD
BRD4 B
794
BRD4 C (Short)
722
BRD4-NUT
NUT
1846
719
F
BRD4 siRNA
- S L S L 1
< LC3B I
< LC3B II
< BRD4 L
< Non-specific
< BRD4 S
GAPDH
1 0.93 1.07 2.39 3.13 LC3II/GAPDH
G
CON BRD4 :siRNA
- 1 1 2 3 4
< LC3B I
< LC3B II
< BRD4 L
< BRD4 S
GAPDH
1 1.05 2.58 1.92 2.50 3.55 LC3II/GAPDH
H
siRNA
CON 1
BRD4 1
BRD4 2
Relative intensity (LC3II/GAPDH)
- CQ
I
- CQ
CON BRD4 CON BRD4 :siRNA
PA-TU-8902
SUIT2
< LC3B I
< LC3B II
BRD4
GAPDH
siRNA
CON 1
BRD4 1
BRD4 2
Relative intensity (LC3II/GAPDH)
PA-TU-8902
SUIT2
J
- CQ
CON BRD4 CON BRD4 :siRNA
GFP-p62
BRD4
GAPDH
siRNA
CON 1
BRD4 1
BRD4 2
Relative intensity (GFP-p62/GAPDH)
N.S.
K
JQ1 (hr)
- 1.5 3 6 9
< LC3B I
< LC3B II
β-actin
1 0.95 0.92 1.77 2.46 LC3II/β-actin

Figure S1 Continued

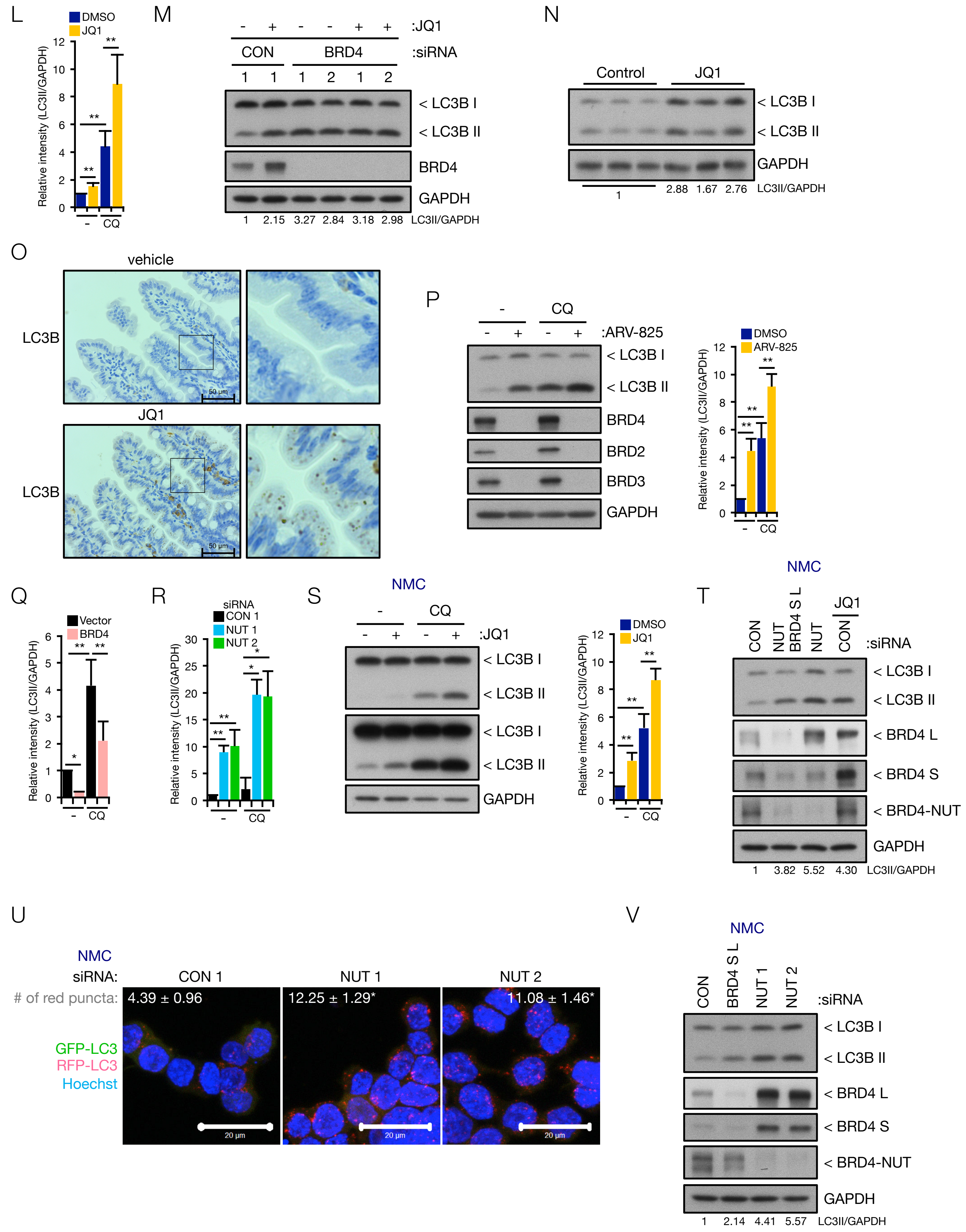

Figure S1, related to Figure 1. BRD4 silencing enhances autophagic flux
(A) Quantification of LC3II signal intensity normalized to GAPDH levels in Fig. 1B.
(B) KP-4 cells were transfected with BRD2 (left) or BRD3 (right) siRNA for 72 hrs.
(C) BRD4 siRNA was transfected into PA-TU-8902, SUIT2, PK-1, PA-TU-8988T, HPNE, and HEK293T cells for 72 hrs.
(D) Proteins (left) and RNA (right) were extracted from FFPE small intestinal tissues of transgenic mice harboring inducible renilla luciferase or BRD4 shRNA. LC3II levels were monitored by western blot analysis (left) and knockdown of BRD4 was confirmed by RT-qPCR (right).
(E) Domain structure of BRD4 isoforms and BRD4-NUT. BD: Bromodomain, ET: Extraterminal domain, CTD: carboxy-terminal domain.
(F, G) KP-4 cells were transfected with siRNA targeting BRD4 short (S) and/or long (L) isoforms for 72 hrs (F). All BRD4 siRNAs used in the experiments target both long and short isoforms (G).
(H) Quantification of LC3II signal intensity normalized to GAPDH levels in Fig. 1E.
(I) PA-TU-8902 (upper left) and SUIT2 (lower left) transfected with BRD4 siRNA were treated with 10 μM CQ for 4 hrs. Quantification of LC3II signal intensity is shown in the middle (PA-TU-8902) and right (SUIT2) panels.
(J) KP-4 cells were transfected with control or BRD4 siRNA. At 24 hrs after siRNA transfection, cells were transfected with GFP-p62 expression vector for 48 hrs. At 16 hrs after GFP-p62 transfection, cells were treated with or without 10 μM CQ for 32 hrs. Quantification of GFP-p62 levels is shown in the right panel.
(K) KP-4 cells were treated with 500 nM of JQ1 and harvested at indicated time points.
(L) Quantification of LC3II signal intensity normalized to GAPDH levels in Fig. 1H.
(M) KP-4 cells transfected with control or BRD4 siRNA were treated with 500 nM JQ1 for 9 hrs.
(N) Proteins were extracted from FFPE small intestinal tissues of control vehicle and JQ1-treated mice.
(O) Immunohistochemical staining for LC3 in small intestinal sections from mice treated with control vehicle or JQ1. Scale bars: 50 μm.
(P) KP-4 cells were treated with 100 nM ARV-825 for 16 hrs in the presence or absence of CQ (10 μM, 4 hrs). Quantification of LC3II levels is shown in the right panel.
(Q, R) Quantification of LC3II signal intensity normalized to GAPDH levels in Fig. 1I (Q) and Fig. 1J (R).
(S) TY-82 cells were treated with 500 nM JQ1 for 9 hrs in the presence or absence of CQ (10 μM, 8 hrs). Quantification of LC3II levels is shown in the right panel.
(T) TY-82 cells were transfected with siRNA against control, NUT alone or NUT together with BRD4 S and L isoforms for 5 days. 500 nM JQ1 was treated for 9 hrs.
(U) TY-82 cells stably expressing RFP-GFP-LC3 were transfected with NUT siRNA for 5 days. The number of GFP-LC3$^-$/RFP-LC3$^+$ puncta normalized to cell number is shown. CON n=133 cells, BRD4 1 n=115 cells, BRD4 2 n=105 cells. Scale bars: 20 μm.
(V) TY-82 cells were transfected with siRNAs targeting BRD4 short and long isoforms or NUT for 5 days.
All data are shown as mean ± SD. A, H-J, L, and P-S: n=3 independent experiments. *$P < 0.01$, **$P < 0.05$, N.S.: no significance.

Figure S2

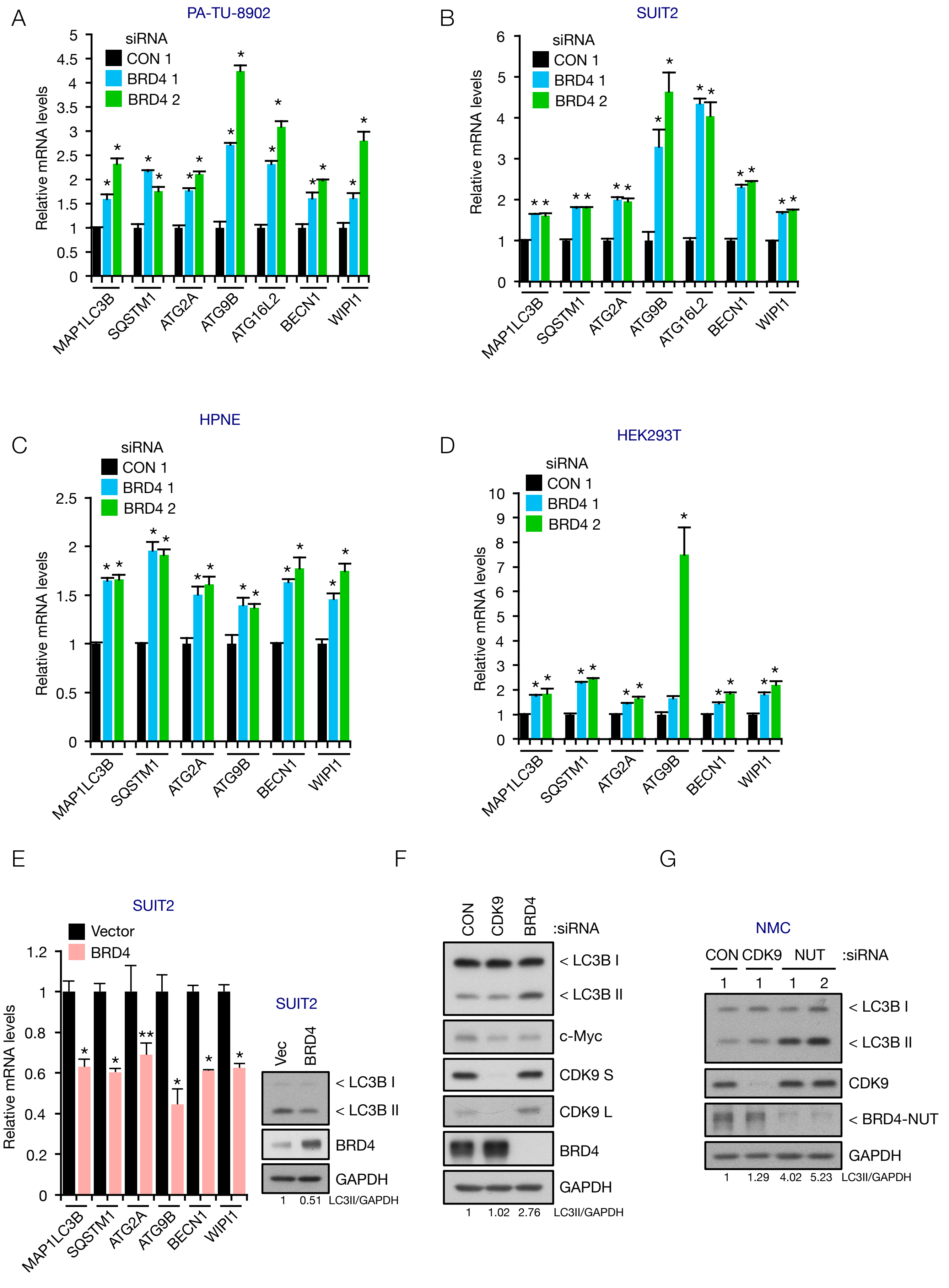

A
PA-TU-8902
siRNA
CON 1
BRD4 1
BRD4 2
Relative mRNA levels
MAP1LC3B
SQSTM1
ATG2A
ATG9B
ATG16L2
BECN1
WIPI1
B
SUIT2
siRNA
CON 1
BRD4 1
BRD4 2
Relative mRNA levels
MAP1LC3B
SQSTM1
ATG2A
ATG9B
ATG16L2
BECN1
WIPI1
C
HPNE
siRNA
CON 1
BRD4 1
BRD4 2
Relative mRNA levels
MAP1LC3B
SQSTM1
ATG2A
ATG9B
BECN1
WIPI1
D
HEK293T
siRNA
CON 1
BRD4 1
BRD4 2
Relative mRNA levels
MAP1LC3B
SQSTM1
ATG2A
ATG9B
BECN1
WIPI1
E
SUIT2
Vector
BRD4
Relative mRNA levels
MAP1LC3B
SQSTM1
ATG2A
ATG9B
BECN1
WIPI1
SUIT2
Vec
BRD4
< LC3B I
< LC3B II
BRD4
GAPDH
1 0.51 LC3II/GAPDH
F
CON
CDK9
BRD4
:siRNA
< LC3B I
< LC3B II
c-Myc
CDK9 S
CDK9 L
BRD4
GAPDH
1 1.02 2.76 LC3II/GAPDH
G
NMC
CON CDK9 NUT :siRNA
1 1 1 2
< LC3B I
< LC3B II
CDK9
< BRD4-NUT
GAPDH
1 1.29 4.02 5.23 LC3II/GAPDH

Figure S2, related to Figure 2. BRD4 is a negative regulator of autophagy gene expression

(A-D) RT-qPCR analyses of PA-TU-8902 (A), SUIT2 (B), HPNE (C), and HEK293T (D) cells transfected with BRD4 siRNA for 72 hrs.

(E) RT-qPCR analysis of SUIT2 cells stably overexpressing BRD4. Right panel shows autophagy inhibition by BRD4 overexpression.

(F) KP-4 cells were transfected with control, CDK9, or BRD4 siRNA for 72 hrs.

(G) TY-82 cells were transfected with siRNA targeting CDK9 or NUT for 5 days.

All data are shown as mean ± SD. A-E: Data are representative of two independent experiments performed in triplicate. $*P < 0.01$, $**P < 0.05$.

Figure S3

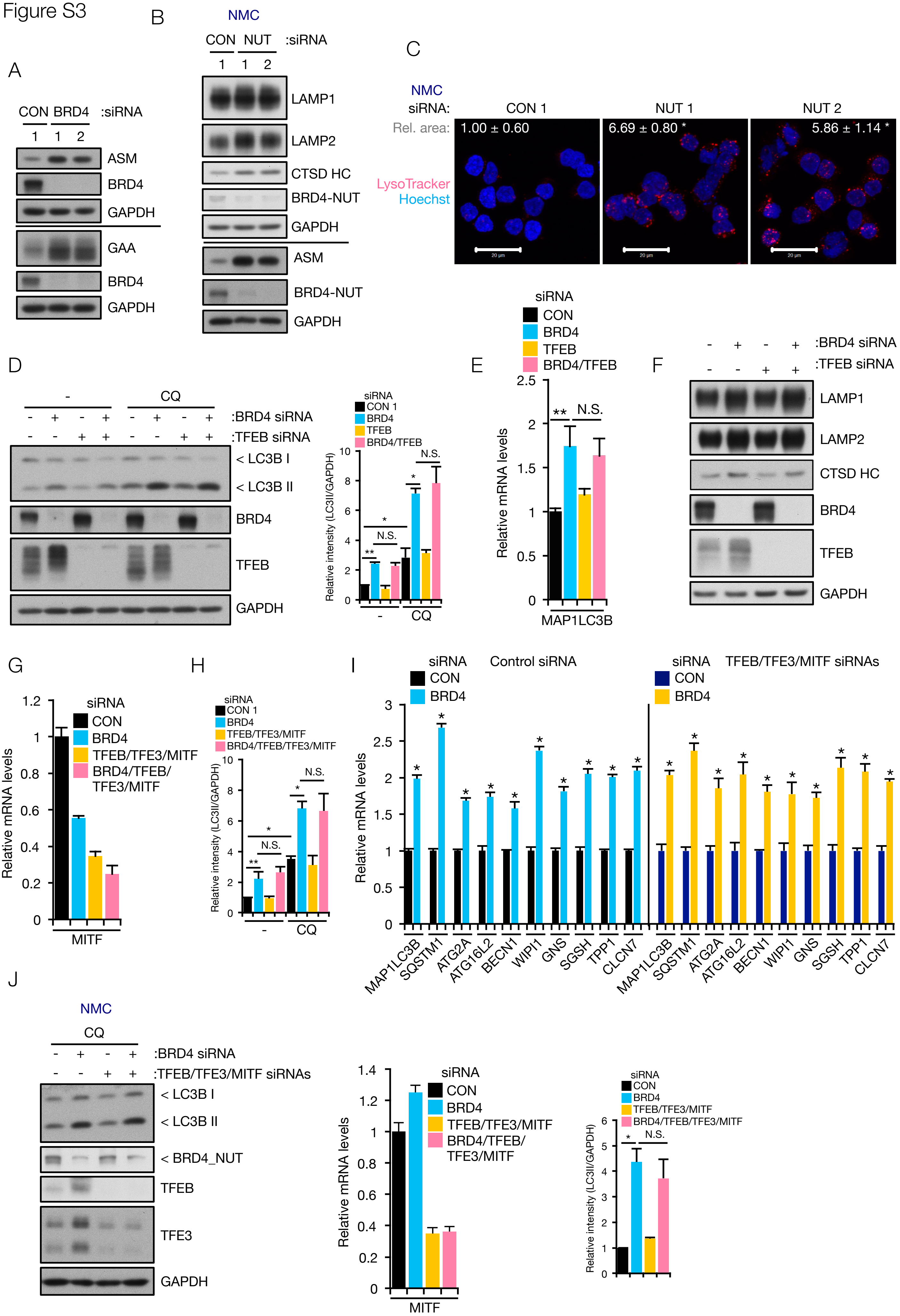
A
CON BRD4 :siRNA
1 1 2
ASM
BRD4
GAPDH
GAA
BRD4
GAPDH
B
NMC
CON NUT :siRNA
1 1 2
LAMP1
LAMP2
CTSD HC
BRD4-NUT
GAPDH
ASM
BRD4-NUT
GAPDH
C
NMC
siRNA:
CON 1
NUT 1
NUT 2
Rel. area:
1.00 ± 0.60
6.69 ± 0.80 *
5.86 ± 1.14 *
LysoTracker
Hoechst
20 μm
D
-
CQ
:BRD4 siRNA
:TFEB siRNA
< LC3B I
< LC3B II
BRD4
TFEB
GAPDH
siRNA
CON 1
BRD4
TFEB
BRD4/TFEB
Relative intensity (LC3II/GAPDH)
N.S.
E
siRNA
CON
BRD4
TFEB
BRD4/TFEB
Relative mRNA levels
MAP1LC3B
F
:BRD4 siRNA
:TFEB siRNA
LAMP1
LAMP2
CTSD HC
BRD4
TFEB
GAPDH
G
siRNA
CON
BRD4
TFEB/TFE3/MITF
BRD4/TFEB/
TFE3/MITF
Relative mRNA levels
MITF
H
siRNA
CON 1
BRD4
TFEB/TFE3/MITF
BRD4/TFEB/TFE3/MITF
Relative intensity (LC3II/GAPDH)
CQ
I
siRNA Control siRNA
CON
BRD4
siRNA TFEB/TFE3/MITF siRNAs
CON
BRD4
Relative mRNA levels
MAP1LC3B
SQSTM1
ATG2A
ATG16L2
BECN1
WIPI1
GNS
SGSH
TPP1
CLCN7
J
NMC
CQ
:BRD4 siRNA
:TFEB/TFE3/MITF siRNAs
< LC3B I
< LC3B II
< BRD4_NUT
TFEB
TFE3
GAPDH
siRNA
CON
BRD4
TFEB/TFE3/MITF
BRD4/TFEB/
TFE3/MITF
Relative mRNA levels
MITF
siRNA
CON
BRD4
TFEB/TFE3/MITF
BRD4/TFEB/TFE3/MITF
Relative intensity (LC3II/GAPDH)
N.S.

Figure S3, related to Figure 3. BRD4 knockdown enhances lysosomal function

(A) KP-4 cells transfected with BRD4 siRNA were subjected to western blot analysis with antibodies against lysosomal proteins.

(B) TY-82 cells transfected with NUT siRNA were subjected to western blot analysis with antibodies against lysosomal proteins.

(C) TY-82 cells transfected with NUT siRNA were stained with LysoTracker Red (100 nM, 4 hrs). Area of LysoTracker$^+$ compartments normalized to cell number is shown (CON n=189 cells, NUT 1 n=101 cells, NUT 2 n=101 cells). Scale bars: 20 μm.

(D) BRD4 siRNA was transfected into KP-4 cells together with siRNA against TFEB followed by treatment with 10 μM CQ for 4 hrs. Quantification of LC3II levels is shown in the right.

(E) BRD4 siRNA was transfected into KP-4 cells together with siRNA against TFEB. MAP1LC3B mRNA levels were monitored at 72 hrs after transfection.

(F) BRD4 siRNA was transfected into KP-4 cells together with siRNA against TFEB for 72 hrs followed by western blot analysis with indicated antibodies against lysosomal proteins.

(G) Confirmation of MITF knockdown in Fig. 3H. KP-4 cells transfected with BRD4 and/or MiT/TFE (TFEB, TFE3, MITF) siRNAs were subjected to RT-qPCR analysis.

(H) Quantification of LC3II levels normalized to GAPDH levels in Fig. 3H.

(I) RT-qPCR analysis of KP-4 cells transfected with BRD4 and/or MiT/TFE (TFEB, TFE3, MITF) siRNAs.

(J) TY-82 cells were transfected with NUT and/or MiT/TFE (TFEB, TFE3, MITF) siRNAs and treated with 10 μM CQ for 8 hrs. Quantification of LC3II levels is shown in the middle panel. Knockdown of MITF was confirmed by RT-qPCR (right panel).

All data are shown as mean ± SD. D, E, H, and J: n=3 independent experiments. I: Data are representative of two independent experiments performed in triplicate. *$P < 0.01$, **$P < 0.05$, N.S.: no significance.

Figure S4

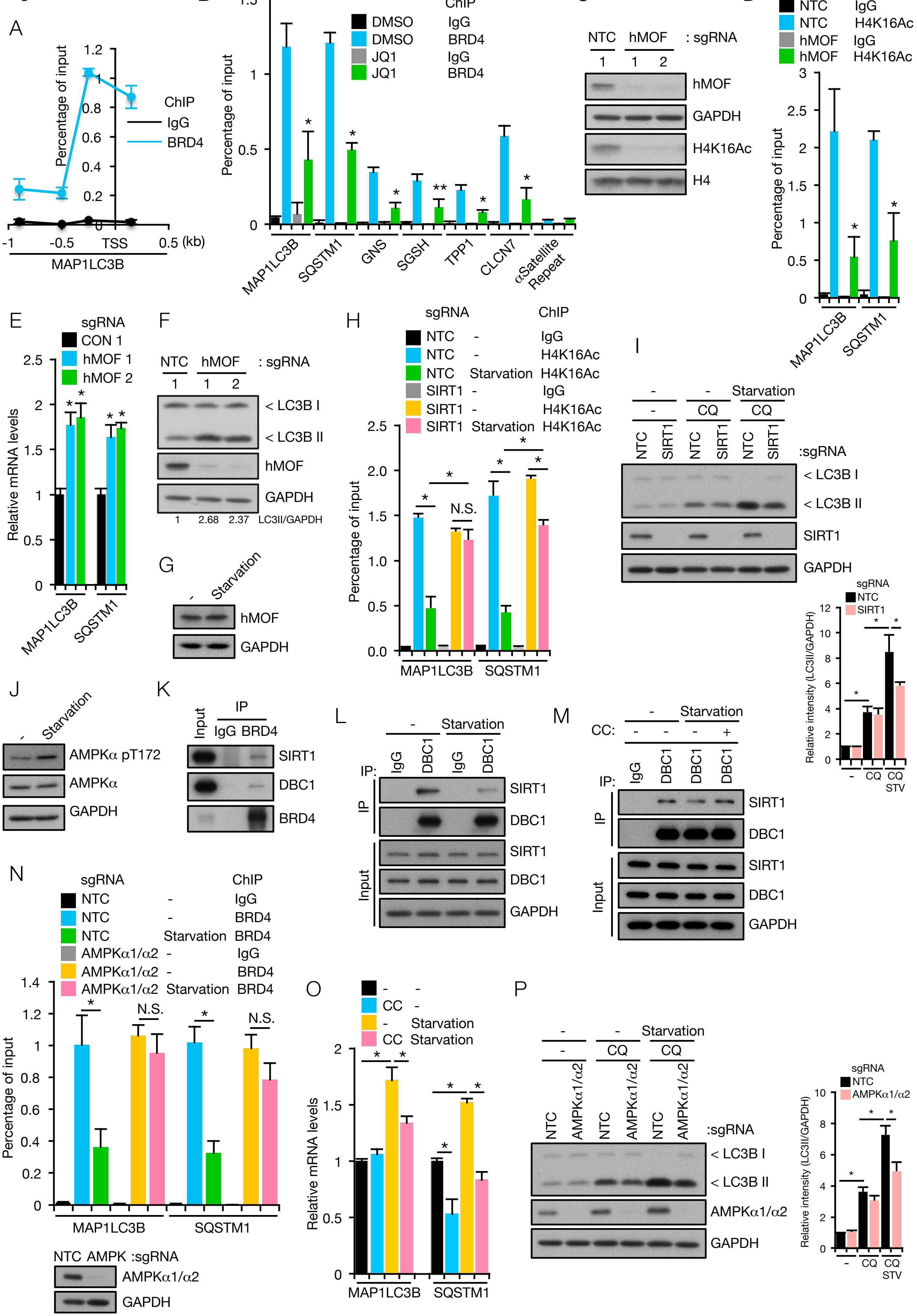
A
B
C
D
E
F
G
H
I
J
K
L
M
N
O
P
ChIP
IgG
BRD4
MAP1LC3B
TSS
Percentage of input
DMSO
JQ1
SQSTM1
GNS
SGSH
TPP1
CLCN7
αSatellite Repeat
NTC
hMOF
: sgRNA
hMOF
GAPDH
H4K16Ac
H4
sgRNA
CON 1
hMOF 1
hMOF 2
Relative mRNA levels
< LC3B I
< LC3B II
1 2.68 2.37 LC3II/GAPDH
Starvation
SIRT1
CQ
:sgRNA
Relative intensity (LC3II/GAPDH)
STV
AMPKα pT172
AMPKα
Input
IP
DBC1
IP:
CC:
AMPKα1/α2
NTC AMPK :sgRNA
CC
N.S.

Figure S4, related to Figure 4. Starvation leads to BRD4 dissociation from autophagy gene promoters
(A) KP-4 cells were subjected to ChIP assay using control IgG and BRD4 antibody followed by qPCR analysis using primers for different regions of MAP1LC3B gene.
(B) KP-4 cells treated with 500 nM JQ1 for 9 hrs followed by ChIP assay with BRD4 antibody.
(C-F) KP-4 cells infected with Cas9/hMOF sgRNA were subjected to western blot analyses with hMOF and H4K16Ac (C) and LC3 (F) antibodies, ChIP assay with H4K16Ac antibody (D), and RT-qPCR analysis (E).
(G) KP-4 cells were starved for 4 hrs followed by western blot analysis with hMOF antibody.
(H) KP-4 cells infected with Cas9/SIRT1 sgRNA were starved for 4 hrs followed by ChIP assay with H4K16Ac antibody.
(I) KP-4 cells infected with Cas9/SIRT1 sgRNA were treated with 10 μM CQ for 4 hrs. At 2 hrs after CQ treatment, cells were subjected to starvation for 2 hrs in the presence of CQ. Quantification of LC3II levels is shown in the lower right panel.
(J) KP-4 cells were starved for 4 hrs and subjected to western blot analysis.
(K) Cell extracts from KP-4 cells were subjected to immunoprecipitation with BRD4 antibody.
(L) Lysates from KP-4 cells starved for 4 hrs were subjected to immunoprecipitation with DBC1 antibody.
(M) KP-4 cells pre-treated with 10 μM Compound C (CC, 3 hrs) were starved for 4 hrs in the presence of 10 μM CC. Cell lysates were then subjected to immunoprecipitation with DBC1 antibody.
(N) KP-4 cells infected with Cas9/AMPKα1/α2 sgRNAs were starved for 4 hrs followed by ChIP assay with BRD4 antibody. Western blot shows efficient AMPKα1/α2 depletion in Cas9/AMPKα1/α2 sgRNA-infected cells.
(O) KP-4 cells pre-treated with 10 μM Compound C (CC, 3 hrs) were starved for 4 hrs in the presence of 10 μM CC.
(P) KP-4 cells infected with Cas9/AMPKα1/α2 sgRNAs were treated with 10 μM CQ for 4 hrs. At 2 hrs after CQ treatment, cells were subjected to starvation for 2 hrs in the presence of CQ. Quantification of LC3II levels is shown in the right panel.
All data are shown as mean ± SD. B, D, E, I, N-P: n=3 independent experiments. A and H: Data are representative of two independent experiments performed in triplicate. *P < 0.01, **P < 0.05, N.S.: no significance.

Figure S5

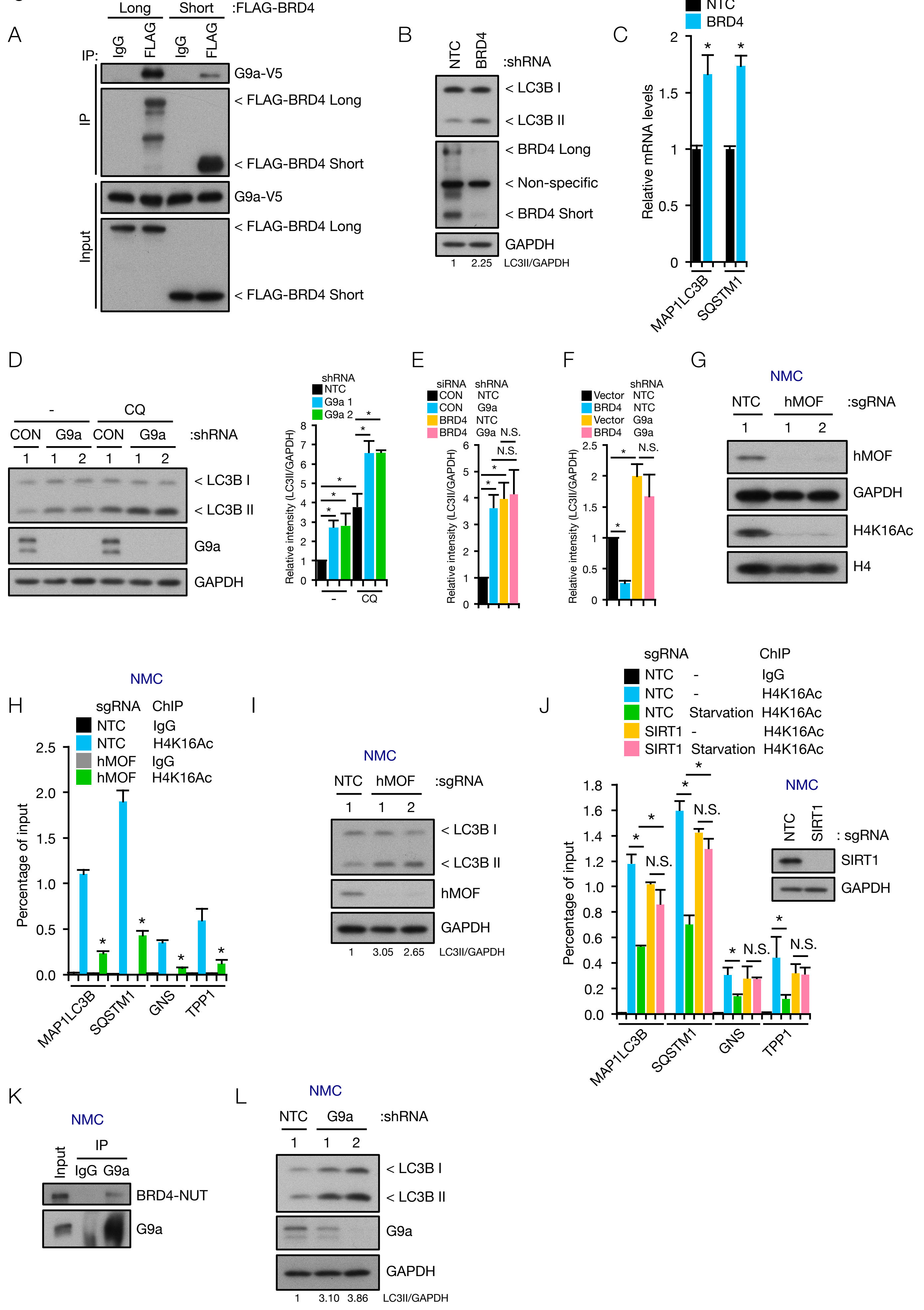
A
Long
Short
:FLAG-BRD4
IP:
IgG
FLAG
IP
Input
G9a-V5
< FLAG-BRD4 Long
< FLAG-BRD4 Short
B
NTC
BRD4
:shRNA
< LC3B I
< LC3B II
< BRD4 Long
< Non-specific
< BRD4 Short
GAPDH
1
2.25
LC3II/GAPDH
C
shRNA
NTC
BRD4
Relative mRNA levels
MAP1LC3B
SQSTM1
D
-
CQ
CON
G9a
:shRNA
< LC3B I
< LC3B II
G9a
GAPDH
shRNA
NTC
G9a 1
G9a 2
Relative intensity (LC3II/GAPDH)
E
siRNA
shRNA
CON
BRD4
NTC
G9a
N.S.
F
Vector
BRD4
G
NMC
NTC
hMOF
:sgRNA
hMOF
GAPDH
H4K16Ac
H4
H
sgRNA
ChIP
IgG
H4K16Ac
Percentage of input
MAP1LC3B
SQSTM1
GNS
TPP1
I
< LC3B I
< LC3B II
1
3.05
2.65
LC3II/GAPDH
J
Starvation
SIRT1
: sgRNA
K
Input
IP
IgG
G9a
BRD4-NUT
L
3.10
3.86

Figure S5, related to Figure 5. BRD4 represses autophagy gene expression through G9a
(A) HEK293T cells transfected with G9a-V5 together with FLAG-BRD4 long or short isoform were subjected to immunoprecipitation with control IgG or FLAG antibody.
(B, C) Validation of inducible BRD4 knockdown cells. KP-4 cells harboring inducible control or BRD4 shRNA were treated with 500 ng/ml doxycycline (DOX) for 4 days and subjected to western blot (B) and RT-qPCR (C) analyses.
(D) KP-4 cells infected with lentivirus expressing G9a shRNA were treated with 10 μM CQ for 4 hrs. Quantification of LC3II levels is shown in the right panel.
(E, F) Quantification of LC3II signal intensity normalized to GAPDH levels in Fig. 5H (E) and Fig. 5I (F).
(G-I) TY-82 cells were infected with Cas9/hMOF sgRNA followed by western blot analysis with hMOF and H4K16Ac (G) and LC3B (I) antibodies and ChIP assay with H4K16Ac antibody (H).
(J) TY-82 cells infected with Cas9/SIRT1 sgRNA were starved for 4 hrs followed by ChIP assay with H4K16Ac antibody. Western blot shows efficient SIRT1 depletion in Cas9/SIRT1 sgRNA-infected cells.
(K) Cell extracts from TY-82 cells were immunoprecipitated with G9a antibody.
(L) TY-82 cells were infected with shRNA targeting G9a followed by western blot analysis.
All data are shown as mean ± SD. C-F: n=3 independent experiments. H and J: Data are representative of two independent experiments performed in triplicate. $^{*}P < 0.01$, $^{**}P < 0.05$, N.S.: no significance.

Figure S6

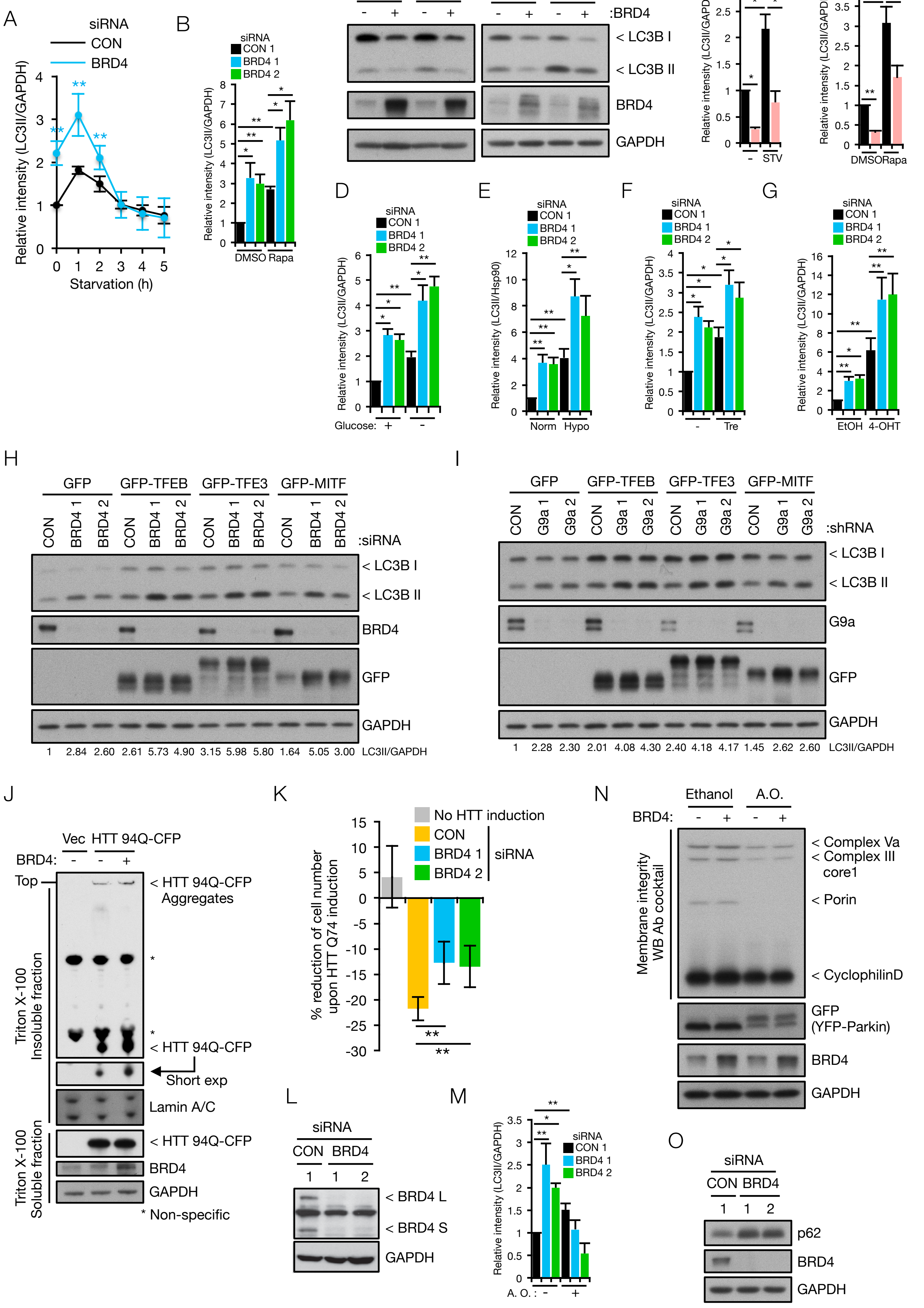

A
siRNA
CON
BRD4
Relative intensity (LC3II/GAPDH)
Starvation (h)
B
siRNA
CON 1
BRD4 1
BRD4 2
Relative intensity (LC3II/GAPDH)
DMSO Rapa
C
- Starvation DMSO Rapa
:BRD4
< LC3B I
< LC3B II
BRD4
GAPDH
Vector
BRD4
Relative intensity (LC3II/GAPDH)
STV
DMSO Rapa
D
siRNA
CON 1
BRD4 1
BRD4 2
Relative intensity (LC3II/GAPDH)
Glucose: + -
E
siRNA
CON 1
BRD4 1
BRD4 2
Relative intensity (LC3II/Hsp90)
Norm Hypo
F
siRNA
CON 1
BRD4 1
BRD4 2
Tre
G
siRNA
CON 1
BRD4 1
BRD4 2
Relative intensity (LC3II/GAPDH)
EtOH 4-OHT
H
GFP GFP-TFEB GFP-TFE3 GFP-MITF
CON BRD4 1 BRD4 2
:siRNA
< LC3B I
< LC3B II
BRD4
GFP
GAPDH
1 2.84 2.60 2.61 5.73 4.90 3.15 5.98 5.80 1.64 5.05 3.00 LC3II/GAPDH
I
GFP GFP-TFEB GFP-TFE3 GFP-MITF
CON G9a 1 G9a 2
:shRNA
< LC3B I
< LC3B II
G9a
GFP
GAPDH
1 2.28 2.30 2.01 4.08 4.30 2.40 4.18 4.17 1.45 2.62 2.60 LC3II/GAPDH
J
Vec HTT 94Q-CFP
BRD4: - - +
Top
< HTT 94Q-CFP Aggregates
< HTT 94Q-CFP
Short exp
Lamin A/C
< HTT 94Q-CFP
BRD4
GAPDH
Triton X-100 Insoluble fraction
Triton X-100 Soluble fraction
* Non-specific
K
No HTT induction
CON
BRD4 1
BRD4 2
siRNA
% reduction of cell number upon HTT Q74 induction
L
siRNA
CON BRD4
1 1 2
< BRD4 L
< BRD4 S
GAPDH
M
siRNA
CON 1
BRD4 1
BRD4 2
Relative intensity (LC3II/GAPDH)
A. O. : - +
N
Ethanol A.O.
BRD4: - + - +
Membrane integrity WB Ab cocktail
< Complex Va
< Complex III core1
< Porin
< CyclophilinD
GFP (YFP-Parkin)
BRD4
GAPDH
O
siRNA
CON BRD4
1 1 2
p62
BRD4
GAPDH

Figure S6, related to Figure 6. Effect of BRD4 silencing on stimulus-dependent and selective autophagy

(A, B) Quantification of LC3II signal intensity normalized to GAPDH levels in Fig. 6A (A) and Fig. 6B (B).

(C) KP-4 cells stably overexpressing BRD4 were starved for 90 min (left western blot) or treated with 500 nM rapamycin for 24 hrs (right western blot). Quantification of LC3II levels is shown.

(D-G) Quantification of LC3II signal intensity normalized to GAPDH or HSP90 levels in Fig. 6C (D), Fig. 6D (E), Fig. 6E (F), and Fig. 6F (G).

(H, I) HEK293T cells were transfected with BRD4 siRNA (H) or infected with G9a shRNA (I) followed by transfection with MiT/TFE expression vectors.

(J) KP-4 cells expressing BRD4, rtTA, and Tre-tight-HTT Q94-CFP were treated with 1 μg/ml DOX for 10 hrs. At 48 hrs after removal of DOX, cells were harvested and separated into TritonX-100 soluble and insoluble fractions.

(K) KP-4 pLVX-GFP-HTT Q74 and control parental cells were transfected with control or BRD4 siRNA for 72 hrs. At 12 hrs after transfection, cells were treated with 100 ng/ml DOX for 60 hrs. Cell number of mutant HTT expressing cells was normalized to that of parental cells and presented as percentage of reduction in cell number upon mutant HTT induction.

(L) Confirmation of efficient BRD4 knockdown in Fig. 6H.

(M) Quantification of LC3II signal intensity normalized to GAPDH levels in Fig. 6I.

(N) KP-4 cells expressing YFP-parkin were infected with control or BRD4 expression vector. Cells were treated with 1 μM Antimycin A and 1 μM Oligomycin for 8 hrs.

(O) KP-4 cells were transfected with BRD4 siRNA for 72hrs.

All data are shown as mean ± SD. A-E, G, and M: n=3 independent experiments. F: n=4 independent experiments. K: n=5 independent experiments. $^*P < 0.01$, $^{**}P < 0.05$.

Figure S7

A

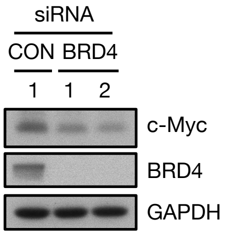

B

Gene Symbol	Fold change BRD4 1 vs Con 1	P value BRD4 1 vs Con 1	Fold change BRD4 2 vs Con 1	P value BRD4 2 vs Con 1
CDK1	-1.669	1.12371E-09	-2.155	1.81448E-19
CDK2	-1.559	1.04614E-09	-1.680	6.13946E-11
CCNA2	-1.292	0.000673956	-1.774	2.91219E-11
CCNB1	-1.756	1.27998E-08	-1.829	1.67277E-09
CCNB2	-2.080	9.82978E-13	-1.879	2.05674E-09
CCND2	-2.709	5.86107E-18	-1.477	0.001657272
CCNE2	-1.412	0.006004696	-1.799	9.32676E-07
CDC25B	-1.326	0.000756346	-1.634	1.39001E-08
CDC25C	-1.829	5.90101E-10	-1.988	8.77715E-12
CDKN1A	1.360	0.001639444	2.967	3.34471E-45
INCA1	1.985	3.1796E-06	2.403	9.04515E-10

C

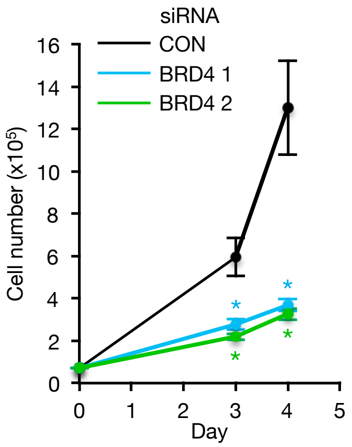

D

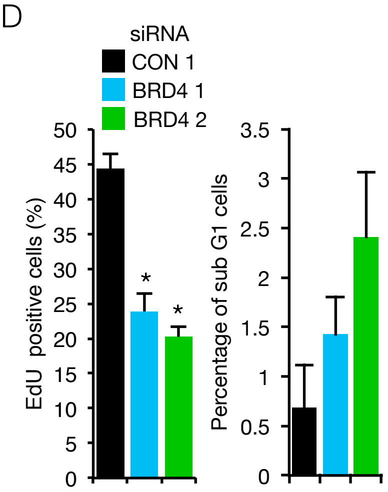

E

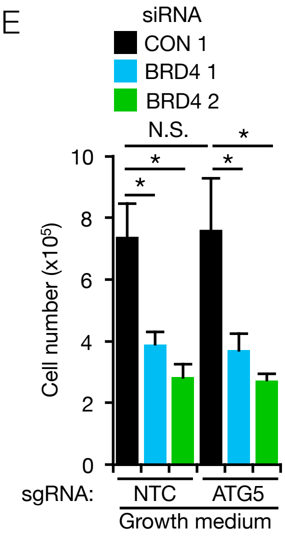

F

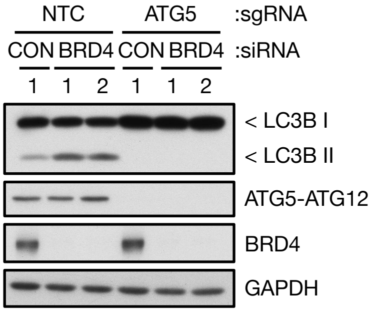

G

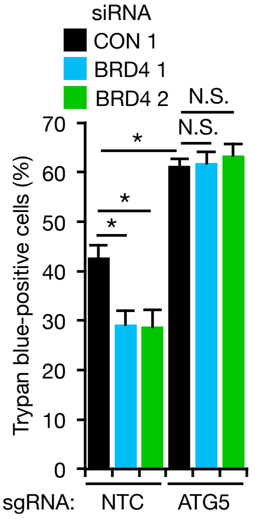

H

Gene Symbol	Fold change BRD4 1 vs Con 1	P value BRD4 1 vs Con 1	Fold change BRD4 2 vs Con 1	P value BRD4 2 vs Con 1	Protein name
SQSTM1	2.499	2.35E-22	3.014	1.77E-32	p62
OPTN	1.827	1.45E-10	1.658	1.55E-07	Optineurin
TAX1BP1	1.220	0.0256	1.261	1.69E-02	TAX1BP1
Calcoco2	-1.180	8.64E-02	-1.038	7.75E-01	NDP52
NBR1	1.215	9.58E-02	1.173	2.08E-01	NBR1
WDFY3	1.192	6.63E-01	1.092	0.838	ALFY

Figure S7, related to Figure 7. BRD4 knockdown sustains mTOR activity during starvation and confers resistance to starvation-induced cell death

(A) KP-4 cells were transfected with BRD4 siRNA for 72hrs.
(B) The results are from the RNA-Seq analysis of KP-4 cells transfected with BRD4.
(C, D) KP-4 cells were transfected with BRD4 siRNA. At 72 and 96 hrs after transfection, cell number was determined (C). At 72 hrs after transfection, EdU incorporation (D, left) assays were conducted. Measurement of subG1 cells (D, right) shows no significant increase in cell death in BRD4 knockdown cells under normal culture conditions.
(E, F) KP-4 cells infected with Cas9/ATG5 sgRNA were transfected with BRD4 siRNA. At 72 hr after transfection, cell number was determined (E). (F) shows efficient depletion of ATG5-ATG12 complex and loss of LC3II in Cas9/ATG5 sgRNA-infected cells.
(G) KP-4 cells infected with Cas9/ATG5 sgRNA were transfected with BRD4 siRNA. Following 48 hr starvation, percentage of dead cells was determined by trypan blue exclusion test.
(H) The results are from the RNA-Seq analysis of KP-4 cells transfected with BRD4 siRNA.
All data are shown as mean ± SD. B-E, G, and H: n=3 independent experiments. *$P < 0.01$, N.S.: no significance.
